# Trend Dynamics of Rheumatic Heart Disease Burden, 1990–2019: Insights From Age-Period-Cohort Modeling and Projections

**DOI:** 10.31083/RCM45318

**Published:** 2026-01-20

**Authors:** Zizheng Liu, Zeye Liu, Ziping Li, Fengwen Zhang, Wenbin Ouyang, Shouzheng Wang, Shenqi Jing, Xiangbin Pan

**Affiliations:** ^1^Department of Structural Heart Disease, National Center for Cardiovascular Disease, China & Fuwai Hospital, Chinese Academy of Medical Sciences & Peking Union Medical College, 100037 Beijing, China; ^2^National Health Commission Key Laboratory of Cardiovascular Regeneration Medicine, 100037 Beijing, China; ^3^Key Laboratory of Innovative Cardiovascular Devices, Chinese Academy of Medical Sciences, 100037 Beijing, China; ^4^National Clinical Research Center for Cardiovascular Diseases, Fuwai Hospital, Chinese Academy of Medical Sciences, 100037 Beijing, China; ^5^State Key Laboratory of Cardiovascular Disease, Fuwai Hospital, National Center for Cardiovascular Diseases, Fuwai Hospital, Chinese Academy of Medical Sciences, and Peking Union Medical College, 100037 Beijing, China; ^6^Department of Cardiac Surgery, Peking University People's Hospital, Peking University, 100037 Beijing, China; ^7^Jiangsu Clinical Medicine Research Institute, The First Affiliated Hospital with Nanjing Medical University, 210029 Nanjing, Jiangsu, China; ^8^Department of Medical Informatics, School of Biomedical Engineering and Informatics, Nanjing Medical University, 210029 Nanjing, Jiangsu, China; ^9^Institute of Medical Informatics and Management, Nanjing Medical University, 210029 Nanjing, Jiangsu, China; ^10^Jiangsu Province Engineering Research Center for Chronic Disease Big Data Application and Intelligent Health Service, 210029 Nanjing, Jiangsu, China

**Keywords:** rheumatic heart disease, Global Burden of Disease Study, mortality, disability-adjusted life years, age–period–cohort analysis

## Abstract

**Background:**

Rheumatic heart disease (RHD) is a global autoimmune disease that contributes significantly to cardiovascular mortality. However, a comprehensive investigation into age-specific mortality patterns across diverse regions remains limited. To address this issue, this study aimed to investigate alterations in RHD mortality and disease burden measured by disability-adjusted life years (DALY), and modifiable risk factors across 204 countries and regions during the preceding three decades. Additionally, this study endeavored to forecast the trends for RHD in the coming decade and to explore the associations with the age, period, and birth cohort by analyzing data from the Global Burden of Disease (GBD) 2019.

**Methods:**

We present up-to-date mortality and DALY data for RHD sourced from the GBD 2019 data. We employed the age–period–cohort (APC) model to assess local and net drift, as well as the influences of age, period, and birth cohort. Additionally, we examine modifiable risk factors and provide projections for RHD mortality trends in the coming decade.

**Results:**

Age-standardized mortality rates for RHD exhibited a net drift ranging from –5.59 (95% confidence interval (CI): –5.84 to –5.34) in high–middle sociodemographic index (SDI) regions, to –2.34 (95% CI: –2.42 to –2.25) in low SDI regions. Comparable trends were observed with DALY. High systolic blood pressure was the major metabolic risk factor in both 1990 and 2019. Projections indicate a global reduction in RHD mortality rates over the coming decade. Nevertheless, individuals in low-SDI regions are projected to bear a substantial mortality burden in both 2019 and 2029, accentuating a widening sex disparity.

**Conclusions:**

In summary, this study found that age, period, and birth cohort effects for RHD were positive globally, except for low SDI regions. The widening health disparities between regions indicate an imminent threat of significant disease burden. Thus, this study underscores the imperative requirement for targeted interventions, enhanced healthcare accessibility, and sex-sensitive strategies to alleviate the burden of death and disability associated with RHD, particularly in low SDI regions.

## 1. Introduction 

Rheumatic heart disease (RHD) stands as an autoimmune disease with deleterious effects on the heart [1], especially affecting individuals aged <25 years [2]. Globally, RHD afflicts an estimated 33 million people, with over 95% of cases occurring in countries with moderate to low income levels. RHD remains a primary contributor to cardiovascular morbidity and mortality, with India, China, Pakistan, the United States, and Japan reporting the highest mortality rates. These statistics highlight the significant health challenge posed by RHD. However, the disease burden shows substantial regional and national variations [3, 4]. To comprehensively address this public health issue, it is imperative to analyze the epidemiological trajectory of RHD within different age groups and sociodemographic contexts. Such an analysis can highlight disparities in healthcare systems, guide the formulation of effective prevention and control strategies, and inform resource allocation for healthcare interventions [5, 6].

While prior research has evaluated the global burden of RHD in terms of morbidity, prevalence, and associated factors [7], a comprehensive investigation of age-specific mortality patterns across diverse countries and regions is still lacking. Moreover, the age-period-cohort (APC) model has yet to be applied to assess the epidemic dynamics of RHD on a global scale. Incorporating key components like net drift, which encapsulates both calendar time and cohort effects, and local drift, which denotes temporal variations in age-specific death rates [8], is crucial given the sensitivity of RHD to economic and social development [3]. Furthermore, credible evidence on the trends in major modifiable RHD risk factors across countries and regions remains scarce. This is a crucial element in devising effective control strategies.

The present study, as part of the Global Burden of Diseases, Injuries, and Risk Factors Study (GBD) partner network, attempts to bridge these critical gaps in the literature. We carried out a novel analysis of age-specific RHD death and disability burden from 1990 to 2019 in 204 countries and regions, differentiated by socio-demographic index (SDI). Leveraging the APC model, we analyzed changes in RHD mortality and the associated burden of disability-adjusted life years (DALYs) across diverse SDI levels over the past three decades. Our investigation encompasses an exploration of age, period, and cohort effects, while also evaluating local drift to determine the variation in distribution of disease, death, and disability burden among different groups. Additionally, we examine the modifiable RHD risk factors in regions with different SDI profiles. Finally, we provide projections for RHD mortality trends in each SDI region for the coming decade.

## 2. Materials and Methods

### 2.1 Data and Definitions

The data analyzed in this research originates from the 2019 GBD database and was obtained using the query tool from the Global Health Data Exchange (GHDx), which can be found at https://ghdx.healthdata.org/gbd-2019 [9]. We adhered to the methodological framework and analytical strategies used in GBD 2019 for the current analysis. The GBD 2019 offers an extensive and current collection of epidemiological information on 369 diseases and injuries, along with 87 risk factors. Importantly, it includes comparative data covering the period from 1990 to 2019 for 204 countries and regions globally. Detailed application methods for GBD 2019 were previously reported [9]. The Washington University Institutional Review Committee carefully evaluated and authorized the exemption from informed consent, based on the use of anonymized and compiled data within the GBD 2019.

A novel Comparative Risk Assessment (CRA) approach based on a causal framework and a hierarchy of risk variables is presented in GBD 2019. The 87 risk elements included in GBD 2019 are generally divided into three main categories: metabolic factors, behavioral factors, and environmental and occupational exposures. This study focused on evaluating the proportional impact of the three major risk factors associated with deaths from RHD from 1990 to 2019, while also analyzing the percentage change in both all-age and age-standardized mortality rates over this period.

Furthermore, the SDI for each country and region was incorporated into our analysis. This SDI serves as a comprehensive indicator of a country’s or region’s level of development, taking into account elements such as per capita earnings, educational attainment among adults, and the fertility patterns of younger women. The index spans from 0 to 1, with greater numbers indicating a higher socioeconomic standing. In 2019, nations and regions are organized into five separate SDI groups: high, high-middle, middle, lower-middle, and low SDI.

### 2.2 RHD Definition

In accordance with the International Classification of Diseases and Injuries 10th Revision (ICD-10) codes, data related to RHD was aligned with the GBD cause list, specifically codes I01-I09.9 [10].

### 2.3 Statistical Analysis

This research examined mortality patterns associated with RHD from 1990 to 2019 by utilizing diverse epidemiological metrics, such as the death/DALYs counts or rates across different age groups, genders, and risk factors. The 95% uncertainty intervals (UIs) for each GBD estimate are calculated using the values for the 2.5th and 97.5th percentiles obtained from 1000 posterior distribution samples [11]. For consistency, the study population was divided into five separate age brackets (0–19, 20–39, 40–59, 60–79, and over 80 years) to determine the death rate in each category.

We used APC models to examine potential patterns in death rates and DALYs by age, period, and birth cohort [12], with the goal of revealing the interaction between age-related biological parameters and the impact of societal and technical factors on disease tendencies. Numerous chronic diseases have been studied using this comprehensive methodology, which is frequently difficult to achieve with conventional epidemiological methodologies [13, 14, 15]. APC analyses were performed with R software (version 4.3.0, R Foundation for Statistical Computing, Vienna, Austria) using the “APC” package, following methods described in the existing literature [16, 17]. Analyses were based on the intrinsic estimator method, a commonly used approach to address the non-identifiability problem in APC models.

In this study, the APC model’s inputs were RHD deaths/DALYs/number and population data for each region or country from 1990 to 2019. In general, the input data, encompassed 22 age groups (from 0–4 to >80 years old with 5-year age group intervals), 6 periods cohorts [from 1990–1994 (median, 1992) to 2015–2019 (median, 2017) with 5-year intervals] and 24 birth cohorts [from 1898–1902 (median, 1900) to 2013–2017 (median, 2015) with 5-year intervals]. The 1990–1994 period and 1953–1957 cohort were set as the reference period and cohort in this study, respectively.

The fitted APC model evaluated the overall temporal trends in death rates and DALYs while taking into account the interaction of age, period, and cohort. These trends were quantified using the concept of net drift, which combines the influence of calendar time and consecutive cohort effects [8]. Concurrently, the APC model measured the annual fractional variation in death rates at a given age (i.e., the local drift of death rates, expressed as an annual percentage) to evaluate the temporal trends in death rates for particular age groups in order to investigate the evolving birth cohort effect. By contrasting age-specific rates in each period (or cohort) with a reference period (or cohort), relative risk was calculated. Significance in annual fractional variation was assessed using the Wald chi-square test.

Subsequently, leveraging GBD data spanning from 1990 to 2019, we projected RHD mortality burdens from 2020 to 2030 using a Bayesian APC (BAPC) model. To mitigate possible excessive dispersion in period effects, we used an inverse gamma distribution prior for the data. The BAPC model, known for its superior predictive performance [18, 19], was executed using the R packages “BAPC” (version 0.0.36) and “INLA” (version 22.05.07) within the R software environment [20].

All statistical tests were two-tailed, with statistical significance defined as *p* < 0.05. The data analysis was conducted using R Software version 4.3.0.

## 3. Results

### 3.1 Global, Regional and Country Trends in RHD Mortality and DALYs From 1990 to 2019

**Fig. 1.**
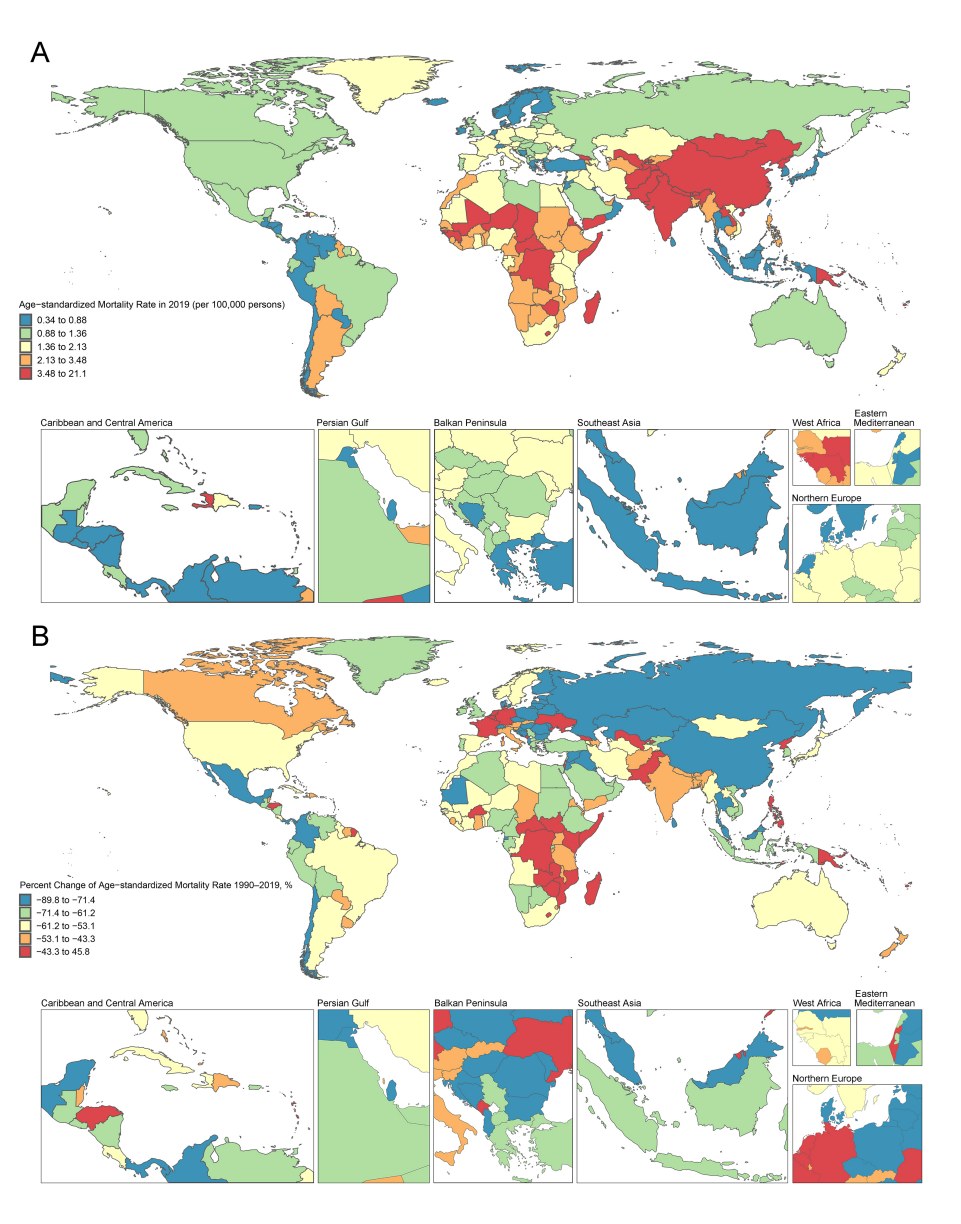
**RHD age-standardized mortality rate (per 100,000 persons) in 2019 and percent change (%) from 1990 to 2019 in 204 countries and territories**. (A) World map of age-standardized mortality rate for RHD in 2019; (B) A world map showing the percentage change in the age-standardized mortality rate for RHD from 1990 to 2019.

Over the preceding three decades, the global number of RHD-related deaths decreased from 362.2 (326.3 to 408.2) thousand in 1990 to 305.7 (95% uncertainty interval (UI): 259.2 to 340.5) thousand in 2019, constituting a 16.0% reduction. In 2019, age-standardized mortality rates ranged from 1.13 (95% UI: 0.98 to 1.24) per 100,000 in high SDI countries to 8.50 (95% UI: 6.99 to 10.2) per 100,000 in low SDI countries. Furthermore, from 1990 to 2019, the global age-standardized mortality rate showed a net drift ranging from –5.59% per year (95% confidence interval (CI): –5.84% to –5.34%) in high-middle SDI countries to –2.34% per year (95% CI: –2.42% to –2.25%) in low SDI countries (Table 1 and Fig. 1). When accounting for the age standardization rate and the net drift of DALY, the global age-standardized DALY rates in 2019 varied from 22.8 (95% UI: 20.9 to 24.9) per 100,000 in high SDI countries to 275.5 (95% UI: 228.0 to 324.6) per 100,000 in low SDI countries. Analogous to the mortality trends, a negative net drift in DALY was observed globally, ranging from –4.54% (95% CI: –4.67% to –4.40%) per year in high-middle SDI countries to –1.94% (95% CI: –2.06% to –1.82%) per year in low SDI countries (Table 1 and **Supplementary Fig. 1**). Sex differences provide interesting insights, and sex-specific estimates of all-age mortality, age-standardized mortality, and DALYs are summarized in **Supplementary Table 1**.

**Table 1.** **RHD mortality and DALY trends by SDI quintiles from 1990 to 2019**.

	Global		High SDI		High-middle SDI		Middle SDI		Low-middle SDI		Low SDI	
	1990	2019	1990	2019	1990	2019	1990	2019	1990	2019	1990	2019
Population												
Number, n × 1,000,000	5350 (5239, 5460)	7737 (7483, 7993)	822	1013	1150	1430	1717	2397	1130	1764	528	1128
Percentage of global, %	100.0	100.0	15.4	13.1	21.5	18.5	32.1	39.6	21.1	22.8	9.9	14.6
Deaths												
Number^⁎^, n × 1000	362.2 (326.3, 408.2)	305.7 (259.2, 340.5)	27.2 (25.4, 28.4)	24.6 (20.8, 27.2)	67.3 (62.6, 73.4)	37.0 (33.2, 40.2)	123.2 (109.8, 138.9)	83.0 (70.8, 93.8)	106.0 (89.3, 129.0)	113.5 (85.2, 135.0)	38.4 (30.0, 49.0)	47.5 (39.4, 56.0)
Percentage of global, %	100.00	100.00	7.51	8.06	18.60	12.10	34.00	27.10	29.30	37.10	10.60	15.60
Percent change in deaths 1990–2019, %	–16.0 (–30.0, –2.0)		–9.0 (–19.0, –2.0)		–45.0 (–53.0, –38.0)		–33.0 (–46.0, –18.0)		7.0 (–18.0, 33.0)		24.0 (0, 55.0)	
All-age mortality rate												
Rate, per 100,000	6.77 (6.10, 7.63)	3.95 (3.35, 4.40)	3.31 (3.09, 3.45)	2.43 (2.06, 2.68)	5.85 (5.44, 6.38)	2.58 (2.32, 2.81)	7.17 (6.40, 8.09)	3.46 (2.95, 3.91)	9.38 (7.91, 11.4)	6.43 (4.83, 7.65)	7.27 (5.67, 9.27)	4.21 (3.49, 4.96)
Percent change in rate 1990–2019, %	–42.0 (–52.0, –32.0)		–27.0 (–34.0, –20.0)		–56.0 (–62.0, –50.0)		–52.0 (–62.0, –41.0)		–31.0 (–47.0, –15.0)		–42.0 (–53.0, –27.0)	
Age-standardized mortality rate												
Rate, per 100,000	8.94 (8.04, 10.1)	3.85 (3.29, 4.29)	2.62 (2.45, 2.73)	1.13 (0.98, 1.24)	6.49 (6.00, 7.10)	1.90 (1.70, 2.07)	12.40 (10.90, 14.10)	3.72 (3.17, 4.23)	16.60 (13.90, 20.40)	8.35 (6.34, 9.95)	14.30 (10.80, 18.70)	8.50 (6.99, 10.20)
Percent change in rate 1990–2019, %	–57.0 (–65.0, –50.0)		–57.0 (–61.0, –54.0)		–71.0 (–75.0, –67.0)		–70.0 (–76.0, –63.0)		–50.0 (–62.0, –36.0)		–40.0 (–54.0, –24.0)	
Net drift of mortality^†^, % per year	–3.15 (–3.27, –3.03)		–4.17 (–4.58, –3.75)		–5.59 (–5.84, –5.34)		–4.53 (–4.69, –4.37)		–2.71 (–2.83, –2.59)		–2.34 (–2.42, –2.25)	
DALY												
Number^⁎^, n × 1000	13,168.3 (11,896.5, 14,634.7)	10,673.9 (9207.4, 12,121.6)	600.5 (575.2, 626.0)	411.4 (372.6, 448.6)	2113.9 (1963.5, 2284.1)	1021.6 (914.6, 1141.7)	4421.7 (3980.5, 4898.8)	2773.1 (2416.9, 3165.5)	4279.6 (3617.0, 5051.6)	4268.6 (3371.7, 5010.5)	1746.2 (1412.1, 2152.1)	2191.9 (1819.1, 2580.1)
Percentage of global, %	100.00	100.00	4.56	3.85	16.10	9.57	33.60	26.00	32.50	40.00	13.30	20.50
Percent change in DALY 1990–2019, %	–19.0 (–31.0, –8.00)		–31.0 (–36.0, –27.0)		–52.0 (–58.0, –46.0)		–37.0 (–47.0, –27.0)		0.0 (–19.0, 18.0)		26.0 (6.00, 50.0)	
All-age DALY rate												
Rate, per 100,000	246.1 (222.4, 273.6)	138.0 (119.0, 156.7)	73.1 (70.0, 76.2)	40.6 (36.8, 44.3)	183.8 (170.7, 198.5)	71.4 (63.9, 79.8)	257.6 (231.9, 285.4)	115.7 (100.9, 132.1)	378.8 (320.2, 447.2)	242.0 (191.1, 284.0)	330.6 (267.4, 407.5)	194.2 (161.2, 228.6)
Percent change in rate 1990–2019, %	–44.0 (–52.0, –36.0)		–44.0 (–48.0, –41.0)		–61.0 (–66.0, –57.0)		–55.0 (–62.0, –47.0)		–36.0 (–48.0, –24.0)		–41.0 (–51.0, –30.0)	
Age-standardized DALY rate												
Rate, per 100,000	283.3 (255.9, 315.3)	132.9 (115.0, 150.3)	59.8 (57.3, 62.3)	22.8 (20.9, 24.9)	189.2 (175.7, 204.8)	56.1 (49.7, 63.6)	341.1 (305.6, 380.5)	112.2 (97.7, 127.8)	513.5 (437.5, 615.9)	266.6 (207.3, 313.8)	468.6 (367.4, 583.9)	275.5 (228.0, 324.6)
Percent change in rate 1990–2019, %	–53.0 (–60.0, –46.0)		–62.0 (–64.0, –59.0)		–70.0 (–74.0, –67.0)		–67.0 (–73.0, –61.0)		–48.0 (–59.0, –38.0)		–41.0 (–52.0, –27.0)	
Net drift of DALY^†^, % per year	–2.67 (–2.78, –2.56)		–3.64 (–3.80, –3.48)		–4.54 (–4.67, –4.40)		–3.77 (–3.93, –3.62)		–2.40 (–2.51, –2.28)		–1.94 (–2.06, –1.82)	

All-age mortality = crude mortality rate. Age-standardized mortality rate is computed by direct standardization with the global standard population in GBD 2019. ^†^ Net drifts are estimates derived from the age-period-cohort model and denote overall annual percentage change in mortality, which captures the contribution of effects from calendar time and successive birth cohorts. ^⁎^ Parentheses for all GBD health estimates indicate 95% uncertainty intervals; parentheses for net drift indicate 95% confidence intervals. **Abbreviations:** DALYs, disability-adjusted life-years; GBD, Global Burden of Diseases, Injuries, and Risk Factors Study; RHD, rheumatic heart disease; SDI, socio-demographic index.

**Fig. 2.**
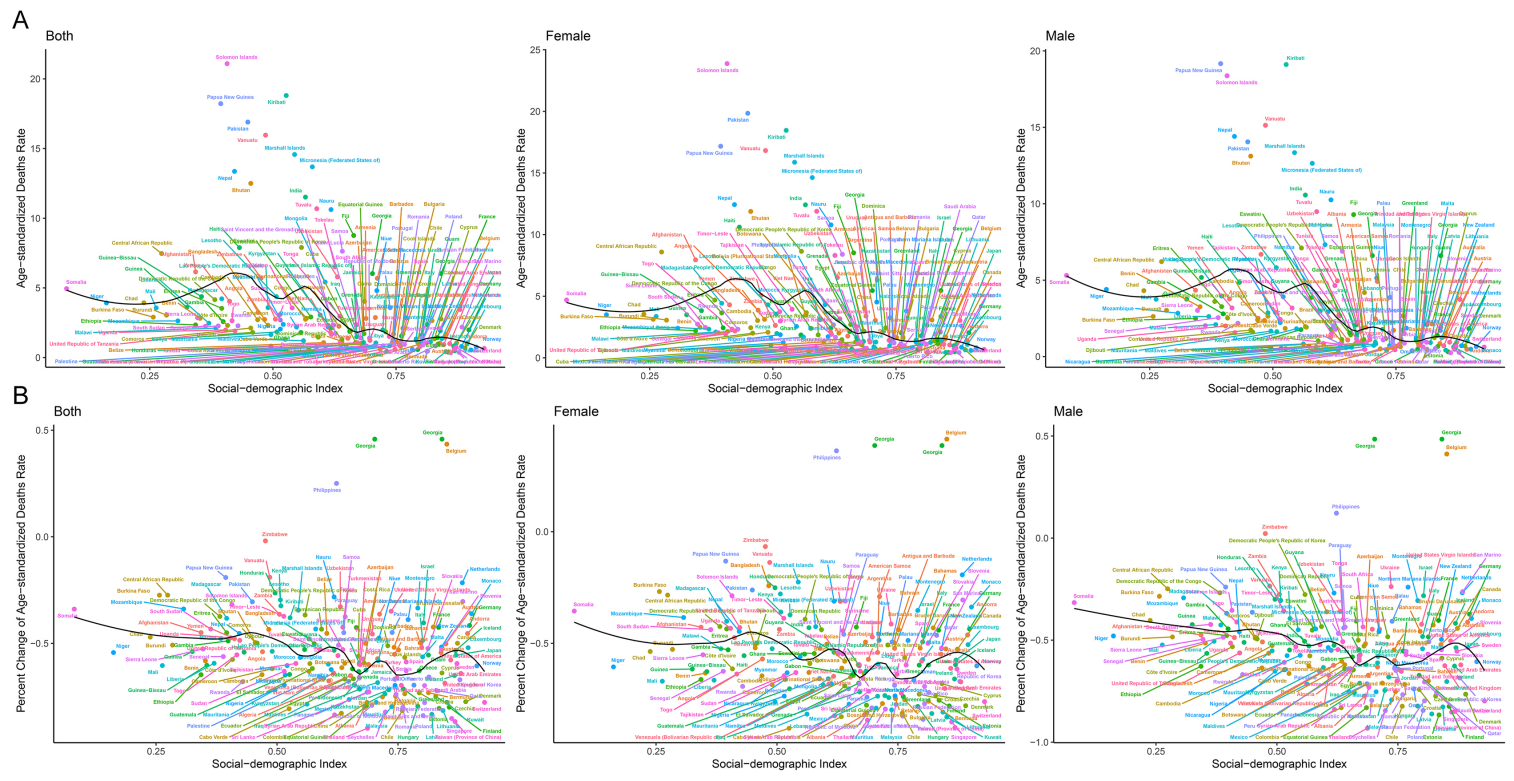
**SDI levels and age-standardized mortality rate (per 100,000 individuals) in 2019, and its percent change (%) from 1990 to 2019 for RHD in 204 countries and territories**. (A) Age-standardized mortality rate for RHD in 2019; (B) Percent change in age-standardized mortality rate for RHD during 1990–2019.

Notable patterns emerged across the 204 countries and regions in this study. India, China, Pakistan, Bangladesh, and Japan collectively accounted for the majority (75.4%) of global deaths. Only five countries displayed either an ascending trend (net drift ≥0) or a relatively stagnant decline (≥–0.5%) in mortality rates. Specifically, the Philippines and Zimbabwe exhibited significant upward trends, signifying a net drift of 2.46% (95% CI: 2.18% to 2.75%) per year and 1.38% (95% CI: 0.86% to 1.89%) per year, respectively. The most substantial DALY losses during 2019 were in India, China, Pakistan, Brazil, and Nigeria. Moreover, the Philippines, Zimbabwe, and Georgia demonstrated an increasing trend in DALY losses. Notably, the trend in DALY losses aligned with that observed in mortality rates. Fig. 2 and **Supplementary Fig. 2** show a comprehensive overview of age-standardized mortality and DALYs distribution and trends for all 204 countries and regions, stratified by SDI levels. These findings underscore the inverse relationship between SDI levels and RHD mortality and DALYs. Countries exhibiting higher SDI levels display lower rates and more substantial reductions in both RHD mortality and DALYs. Clear disparities exist in RHD-related mortality and DALYs across various countries and sexes, contributing to a progressive widening of the gap in RHD burden between countries characterized by high and low SDI levels, as well as between sexes.

### 3.2 Temporal Trends in RHD Mortality and DALYs Across Different Age Groups From 1990 to 2019

**Fig. 3.**
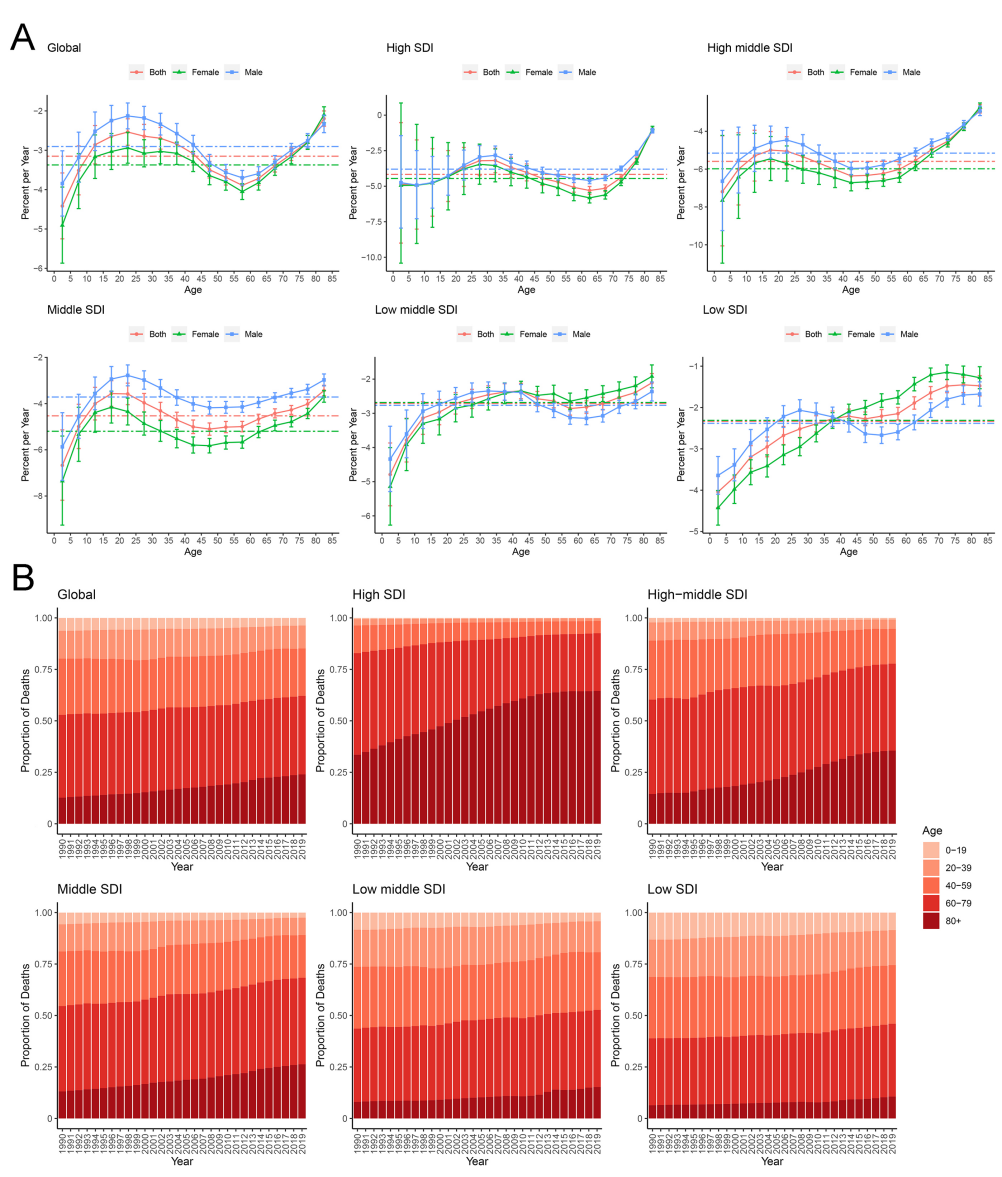
**Local drift in RHD mortality, and age distribution of RHD deaths and by SDI quintiles during 1990–2019**. (A) Local drifts of RHD mortality for age groups 1990–2019, computed from the age–period–cohort model; (B) Temporal change in the relative proportion of RHD deaths across age groups during 1990–2019.

In the past 30 years, global RHD mortality has exhibited consistent declining trends in all age groups, as shown by the local drift in mortality estimated from the APC model (Fig. 3A). The most prominent reductions occurred within the age groups of <5 years (local drift: –4.42%, 95% CI: –5.25% to –3.58%) and 55–60 years (local drift: –3.89%, 95% CI: –4.07% to –3.71%), while the smallest decrease was observed in those aged >80 years (local drift: –2.20%, 95% CI: –2.39% to –2.00%). Of note, females consistently experienced more substantial declines in mortality compared to males, regardless of age.

A gradual shift towards older age groups (>80 years) was observed for global RHD-related mortality, which serves as an indirect marker of improved survival within this demographic. This shift was particularly evident in high and high-middle SDI countries (Fig. 3B). With the exception of Saudi Arabia, Qatar, and the United Arab Emirates, high SDI regions experienced falls in mortality rates for populations <80 years, whereas low SDI countries did not experience improvements in mortality. In low and low-medium SDI countries, where deaths in <60-year-olds comprise around half of all RHD deaths, the potential for future increases in mortality is noteworthy. It is important to highlight that across all SDI levels, the proportion of deaths among older females (>80 years) surpasses that of males, with this disparity being more pronounced in high SDI countries and territories (**Supplementary Fig. 3**). Similar trends are apparent with DALY losses (**Supplementary Fig. 4**).

### 3.3 Age, Period, and Cohort Effects on RHD Mortality and DALYs

**Fig. 4.**
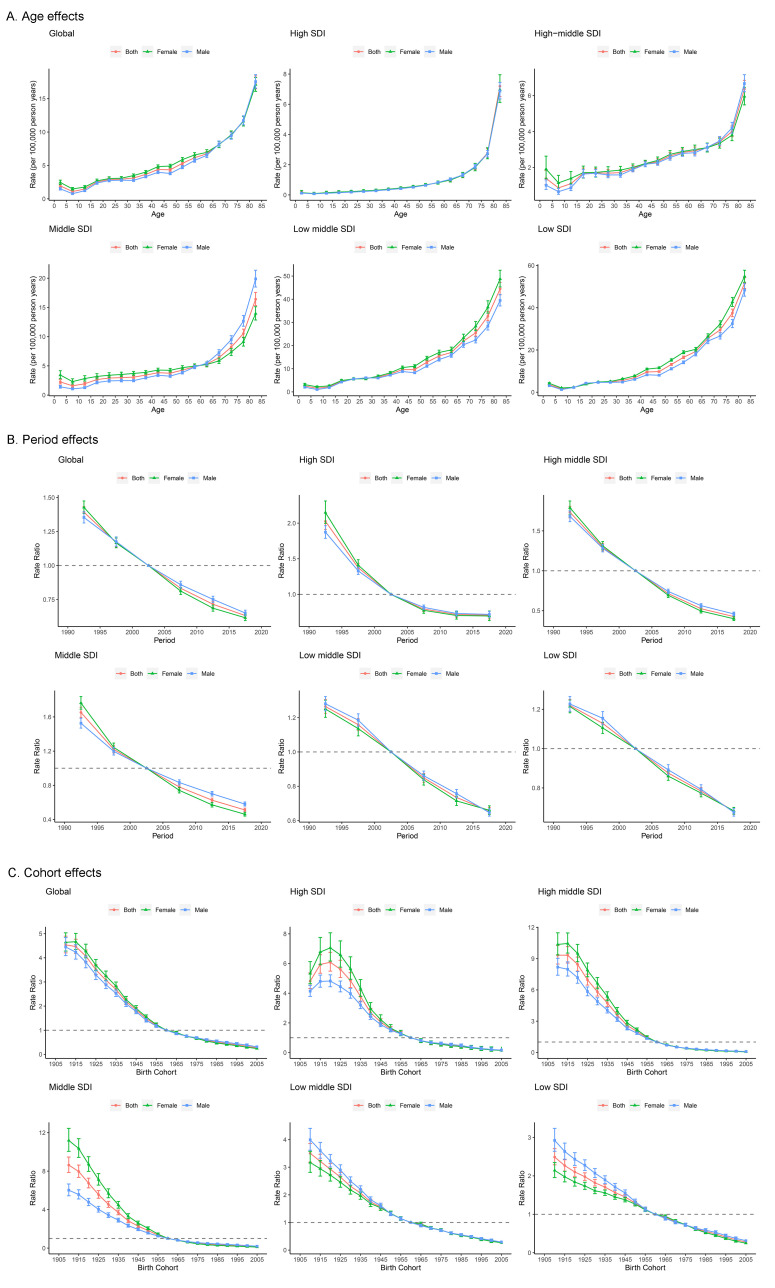
**Parameter estimates of age, period, and cohort effects on RHD mortality by SDI quintiles**. (A) Age effects; (B) Period effects; (C) Cohort effects.

Age effects represent the age-related natural progression of RHD-associated mortality. Across both global and different SDI quintiles, the risk of RHD mortality increases with advancing age. Regions with high SDI manifest the lowest RHD mortality across all age groups. Noteworthy sex disparities in age effects remain nonsignificant across all SDI levels (Fig. 4A).

Period and cohort effects examine the progress of RHD outcomes across different time frames and birth cohorts, respectively. Global period effects show a decreasing mortality risk across all SDI levels, albeit with a more pronounced decline in high SDI countries. However, this reduction has flattened out over the past decade. Similarly, no significant period effects were observed for sex-based differences across all SDI levels (Fig. 4B). On a global scale, the mortality risk from RHD is decreasing among younger birth cohorts. Relevant to this, countries with high SDI experienced a transient surge in the risk of birth cohort effects between 1910 and 1920. In contrast, within older birth cohorts, females show an elevated risk of RHD-related mortality compared to males. This sex disparity gradually decreases in younger birth cohorts (Fig. 4C). The impact of age, period, and cohort effects on trends in DALYs and local drift changes closely mirror the aforementioned patterns (**Supplementary Fig. 5**). Summaries of the APC model analysis encompassing 204 countries and territories are presented in **Supplementary Tables 2,3** in the **Supplementary Material**.

A selection of exemplar countries is presented in **Supplementary Figs. 6,7**. Notably, the United States and Japan represent high SDI nations in North America and Asia, respectively, with both demonstrating favorable outcomes over the past three decades. In these nations, RHD-related deaths have undergone a characteristic shift towards the elderly population, with no apparent gender difference. Overall, the trends observed in period and birth cohorts evoke optimism. For instance, the United States witnessed a substantial reduction in local drift among individuals aged >75 years. The mortality trends in Japan are quite remarkable, with <2% of RHD-related mortality in 2019 occurring in individuals aged <60 years, marking an 85.7% decline from 1990. Of significance, individuals aged >80 years accounted for 80.8% of deaths in Japan, a significantly higher proportion than in the United States (55.0%). China, which is characterized by middle SDI and a substantial population, experienced a comparatively gradual shift over the past three decades in the distribution of deaths from RHD towards older age groups. Notably, the elevated risk of age effects persists in the younger age group (0–5 years), but this cohort also experienced the most rapid decline in mortality (local drift: –11.1%, 95% CI: –14.5% to –7.44%). Finally, Pakistan, representing a low SDI country, is one of the few nations that experienced a shift in the distribution of deaths towards individuals aged <60 years, alongside sluggish improvements in mortality rates among those aged <30. This pattern indicates potential deficiencies in early disease prevention and treatment in Pakistan.

### 3.4 Leading Modifiable Risk Factor and Period Effects on RHD Mortality and DALYs

**Fig. 5.**
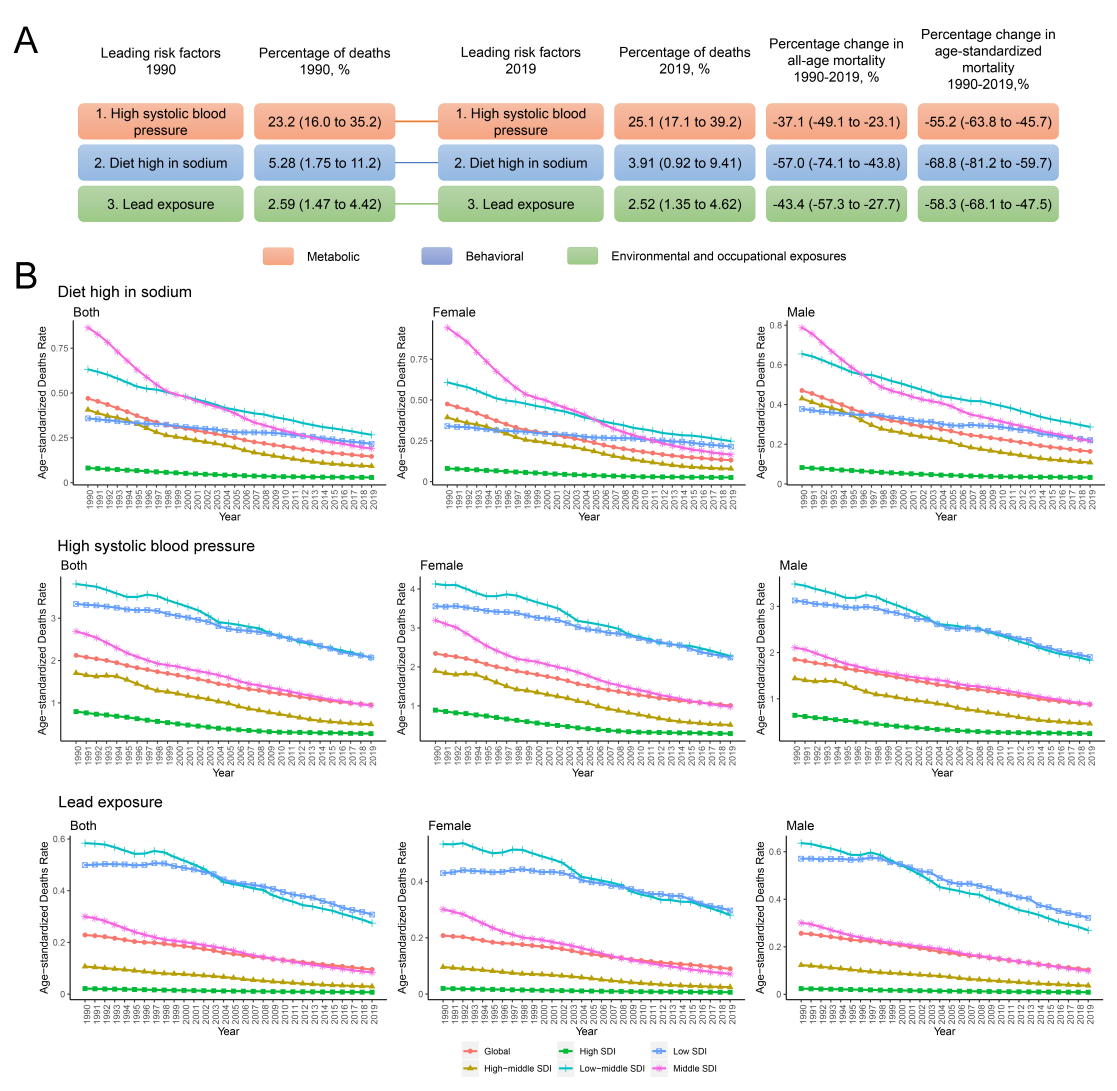
**The three leading risk factors for RHD deaths, and period effects on global age-standardized mortality rates from RHD by SDI quintiles**. (A) The three leading risk factors for global RHD deaths and percentage of total deaths (1990 and 2019), as well as the percentage change in all-age and age-standardized mortality from 1990 to 2019; (B) APC model-derived estimates of period effects on RHD mortality by SDI quintiles from 1990 to 2019.

For the purpose of investigating global trends in RHD mortality and DALYs, we ranked the top three modifiable risk factors for the years 1990 and 2019. Our investigation encompassed the period effects of three leading modifiable risk factors—high sodium diet, high systolic blood pressure (SBP), and lead exposure—on a global scale and within SDI quintiles, as well as across sexes. The results indicate that the predominant metabolic risk factor, high SBP, maintained its ranking in both 1990 and 2019. During the period from 1990 to 2019, RHD mortality associated with high SBP fell by 37.1% (95% UI: –49.1% to –23.1%) across all age groups. Age-standardized mortality also declined by 55.2% (95% UI: –63.8% to –45.7%). A similar trend was noted for the leading behavioral factor—high sodium diet—and lead exposure, which is the major environmental and occupational exposure (Fig. 5A).

On a global scale, encouraging period effects emerged concerning age-standardized mortality rates (Fig. 5B) and DALYs (**Supplementary Fig. 8**) associated with the three leading risk factors over a span of three decades and across all SDI regions. Notably, there was no significant sex difference. Regarding the high sodium diet, the most substantial improvement occurred in middle SDI regions, whereas high and low SDI regions displayed limited improvements. Moreover, the risk of RHD-related mortality attributed to a high sodium diet demonstrated the most pronounced improvement in low SDI regions. For lead exposure, the most significant improvement over the last 30 years occurred in low and low-middle SDI regions, whereas high SDI regions exhibited minimal improvement, albeit presenting with the lowest risk of lead exposure. High SBP emerged as the greatest risk factor. Similar to lead exposure, the most significant improvement for high SBP was observed in low and low-middle SDI regions. Notably, high SDI regions demonstrated substantial improvements across all risk factors. Collectively, these findings underscore the need for greater effort in managing modifiable RHD death-associated risk factors, especially in low and low-middle SDI regions, where progress has been comparatively slow.

### 3.5 Projections of Future RHD Mortality Over the Coming Decade

We next applied Bayesian APC analysis to estimate projections regarding the mortality associated with RHD over the coming decade (see **Supplementary Fig. 9**). According to the model, the global mortality rate attributed to RHD is expected to show a sustained decline over this timeframe. Specifically, the age-standardized mortality rate is anticipated to decrease to 3.06 per 100,000 population by the year 2029, representing a notable decline from 3.86 per 100,000 recorded in 2019. Furthermore, the model predicts a convergence in mortality rates between the sexes.

Nonetheless, it should be noted that in 2029, individuals in low SDI regions will continue to face mortality rates that are 7–fold higher than those in high SDI regions (6.67 vs. 0.93 per 100,000, respectively), and differences between females and males will continue to grow. This disparity highlights the persistent healthcare divide across different SDI regions. Urgent interventions are imperative, particularly within low SDI regions, to alleviate the burden of RHD and address the widening gap in mortality rate between the sexes.

## 4. Discussion

RHD continues to represent a global public health challenge, displaying significant epidemiological variations across countries and regions. This pioneering investigation systematically analyzed RHD mortality and DALYs from 1990 to 2019, employing the APC model to examine age, period, and cohort effects across different SDI levels and sexes. Our findings revealed that countries with higher SDI levels have lower age-standardized mortality/DALYs, and more rapid declines in mortality. Conversely, regions with lower SDI experience higher mortality/DALYs and slower improvements, particularly among females. Projections made with the BAPC model foresee a global decline in RHD mortality rates over the next decade. However, individuals in low SDI regions are expected to face mortality rates over 7–fold higher than their high SDI counterparts in both 2019 and 2029, with a widening sex gap. These trends underscore the urgent need to improve RHD treatment and care in low SDI regions, and the imperative to address expanding health disparities among countries.

RHD stemming from untreated streptococcal pharyngitis is a multifaceted disease influenced by factors affecting its transmission, including access to healthcare and social determinants of health [21, 22]. National-level disparities in social determinants, such as income and education, closely relate to RHD mortality burdens [23]. Moreover, RHD prevalence exhibits significant regional and national variation, and is expected to rise due to increasing rates of rheumatic fever and limited healthcare access [24]. To better understand the RHD burden, a study was conducted in 2015 with GBD data, although it did not consider the impact of SDI on affected individuals [24]. Beyond the prevention of acute rheumatic fever, social and economic determinants of health also challenge the management of chronic RHD. Despite the efficacy of lifelong treatment options, these can nevertheless strain healthcare systems [25].

Our analysis revealed a consistent decline in global RHD-related mortality over the past three decades, with a 16.0% reduction from 1990 to 2019. Nonetheless, countries with higher SDI levels had lower age-standardized mortality rates and more rapid declines in mortality, meaning the health gap with countries in low SDI regions is widening. Countries and regions with lower SDI levels tend to exhibit higher mortality and DALY loss rates, and to experience slower rates of improvement. Consequently, the disparities in health outcomes between countries appear to be expanding. We observed a negative net drift in DALY in the population as a whole, ranging from –4.54% per year in medium-high SDI countries, to –1.94% in low SDI countries. The change in trend for DALY observed in this study is consistent with the trends observed for mortality rates. Therefore, major efforts should be directed towards addressing RHD and improving health outcomes, particularly in low SDI regions. These efforts should emphasize the importance of early detection and intervention strategies, especially in resource-constrained regions. Our study highlights significant sex differences, revealing that females consistently face a greater burden of RHD mortality and DALYs. These findings concur with previous studies that highlighted significant disparities in medical and surgical care for RHD [26].

Analysis of age-specific mortality trends indicates a consistent decline in RHD-related mortality across all age groups, with the most substantial reductions noted in the under 5 and 55–60 year-old groups. This decline signifies improvements in healthcare and disease management, especially in preventing complications among children, and in the provision of health care for the elderly population. The age distribution of deaths in RHD patients was further analyzed to determine trends in patient survival. The findings indicate a reduction in mortality rates for individuals aged <80 years in high SDI regions, while no corresponding improvement was noted in low SDI countries. Therefore, measures should be taken to reduce the risk of future increases in mortality in low and medium SDI countries, where about half of all RHD deaths occur in <60-year-olds. The APC analysis sheds light on the interplay of age-associated biological factors, period effects, and cohort effects on RHD mortality. Age effects indicate an increased RHD mortality risk with advancing age, albeit with higher SDI regions experiencing lower mortality rates across all age groups. Period effects reveal a declining trend in mortality risk, but with varying rates across different SDI levels. Cohort effects indicate improved outcomes in younger birth cohorts, particularly among females, with regional variations. Furthermore, a substantial proportion of patients diagnosed with RHD succumb to this condition due to a combination of chronic RHD and severe complications such as respiratory infections, cardiac insufficiency, and acute pulmonary edema, significantly compromising their health status [26]. The BAPC model was applied to project future trends in RHD mortality rates for the period from 2019 to 2029, with the results showing a decline in mortality rates over this period. Nevertheless, urgent attention is still needed to address RHD and improve health outcomes, especially in low-SDI regions. The proportion of elderly citizens is currently increasing rapidly worldwide, leading to a pronounced shift towards a “deeply aging society”. Furthermore, in 2019, the RHD mortality rates in low-SDI regions were 7–fold higher than in high-SDI regions, and are likely to be again in 2029, indicating persistent health disparities across regions. These findings underscore the dynamic nature of RHD epidemiology and the need for sustained efforts in period-specific interventions. Furthermore, the persistence of sex disparities in older birth cohorts emphasizes the importance of sex-sensitive healthcare approaches.

The GBD database serves as a valuable tool for estimating the worldwide burden of disease, injuries, and hazards. Nevertheless, this study has several limitations. Firstly, the availability of data is restricted in certain areas, particularly in countries with medium or low incomes, potentially affecting the accuracy of estimates. Secondly, data quality varies widely across countries and regions, affecting the precision of results. Lastly, accurate determination of the cause of death can be challenging, especially in countries with low to middle SDI, where death data may be incomplete or unreliable. This can potentially lead to inaccuracies when evaluating disease burden.

## 5. Conclusions

In conclusion, this comprehensive analysis of RHD epidemiology provides valuable insights into the global death and disability patterns of this disease, as well as the risk factors and future trends. Our study highlights the need for targeted interventions, improved access to healthcare, and sex-sensitive strategies to reduce the death and disability burden of RHD, especially in low-income regions. Addressing modifiable risk factors and maintaining current efforts in high SDI regions are essential for effective RHD control and prevention.

## Data Availability

The authors confirm that this study analyzed publicly available datasets. These data can be found here: the Global Burden of Disease (GBD) study (https://vizhub.healthdata.org/gbd-results/).

## Abbreviations

APC, age-period-cohort; CRA, Comparative Risk Assessment; DALY, Disability-Adjusted Life Year; GBD, Global Burden of Diseases; GHDx, Global Health Data Exchange; ICD-10, International Classification of Diseases and Injuries 10th Revision; RHD, rheumatic heart disease; SDI, socio-demographic index; UI, uncertainty interval; CI, confidence interval.

## Supplementary Material

Supplementary material associated with this article can be found, in the online version, at https://doi.org/10.31083/RCM45318.
