# Supplementary material for: Trend Dynamics of Rheumatic Heart Disease Burden, 1990–2019: Insights From Age-Period-Cohort Modeling and Projections
Source: Rev Cardiovasc Med. 2026 Jan 20;27(1):45318. doi: 10.31083/RCM45318 (PMC12873705; doi:10.31083/RCM45318)
Supplement: Supplementary file 1 [file 2153-8174-27-1-45318-s1.zip › Supplementary Material.pdf]

Supplemental Materials

Trend Dynamics of Rheumatic Heart Disease Burden, 1990-2019:

Insights from Age-Period-Cohort Modeling and Projections

List of Content

1. **Supplemental Tables**

- **Supplementary Table 1.** Trends in sex-specific RHD mortality and DALYs across SDI quintiles during 1990-2019.
- **Supplementary Table 2.** The temporal change of deaths from RHD by SDI quintiles in 204 countries and territories during 1990-2019.
- **Supplementary Table 3.** The temporal change of DALYs from RHD by SDI quintiles in 204 countries and territories during 1990-2019.

2. **Supplemental Figures**

- **Supplementary Fig. 1.** Age-standardized DALYs (per 100,000 persons) in 2019 and percent change (%) of age-standardized DALY during 1990-2019 for RHD in 204 countries and territories.
- **Supplementary Fig. 2.** The relationship between SDI levels and the age-standardized DALYs (per 100,000 persons) in 2019 and percent change (%) of age-standardized DALY during 1990-2019 for RHD in 204 countries and territories.
- **Supplementary Fig. 3.** The temporal change in the sex-specific age distribution of deaths from RHD by SDI quintiles during 1990-2019.
- **Supplementary Fig. 4.** The temporal change in the sex-specific age distribution of DALYs from RHD by SDI quintiles during 1990-2019.
- **Supplementary Fig. 5.** Local drifts, age, period, and cohort effects on DALYs from RHD by SDI quintiles.
- **Supplementary Fig. 6.** Age-period-cohort effects of deaths from RHD on exemplar countries.
- **Supplementary Fig. 7.** Age-period-cohort effects of DALYs from RHD on exemplar countries.
- **Supplementary Fig. 8.** The leading risk factor and its period effects on global DALYs from RHD by SDI quintiles.
- **Supplementary Fig. 9.** The age-standardized mortality rate of RHD by SDI quintiles over the upcoming decade predicted by the Bayesian age-period-cohort model.

Supplementary Table 1. Trends in sex-specific RHD mortality and DALYs across SDI quintiles during 1990-2019.

	Global		High SDI		High-middle SDI		Middle SDI		Low-middle SDI		Low SDI	
	1990	2019	1990	2019	1990	2019	1990	2019	1990	2019	1990	2019
Male												
Deaths												
Number*, n×1,000	152.1 (125.1, 184.9)	131.7 (113.5, 159.9)	8.82 (8.43, 9.22)	8.67 (7.77, 9.36)	25.6 (23.3, 28.4)	14.7 (12.8, 16.8)	47.3 (40.0, 54.4)	37.1 (30.5, 45.1)	51.3 (37.7, 71.3)	49.4 (37.8, 67.7)	19.0 (12.0, 29.3)	21.7 (15.8, 30.0)
Percentage of global, %	-0.13 (-0.31, 0.08)		-1.69 (-0.10, 0.05)		-42.7 (-0.53, -0.30)		-21.6 (-0.42, 0.08)		-3.67 (-0.29, 0.26)		14.7 (-0.10, 0.54)	
Percent change of deaths 1990–2019, %	100	100	5.80	6.58	16.8	11.2	31.3	28.2	33.7	37.5	12.5	16.5
All-age mortality rate												
Rate, per 100,000	5.65 (4.64, 6.87)	3.39 (2.92, 4.12)	2.18 (2.08, 2.28)	1.71 (1.54, 1.85)	4.48 (4.07, 4.96)	2.06 (1.79, 2.35)	5.42 (4.58, 6.24)	3.08 (2.53, 3.74)	8.9 (6.54, 12.4)	5.58 (4.26, 7.63)	7.12 (4.49, 11.0)	3.84 (2.79, 5.30)
Percent change of rate 1990–2019, %	-39.9 (-52.0, -25.0)		-21.3 (-28.0, -16.0)		-54.1 (-62.0, -44.0)		-43.2 (-58.0, -22.0)		-37.4 (-54.0, -18.0)		-46.1 (-58.0, -28.0)	
Age-standardized mortality rate												
Rate, per 100,000	8.19 (6.70, 10.0)	3.62 (3.14, 4.37)	2.15 (2.04, 2.25)	1.01 (0.91, 1.10)	5.72 (5.18, 6.36)	1.78 (1.56, 2.03)	9.94 (8.39, 11.5)	3.56 (2.92, 4.32)	16.2 (11.7, 23.2)	7.62 (5.87, 10.6)	14.5 (8.99, 23.9)	8.05 (5.75, 11.8)
Percent change of rate 1990–2019, %	-55.8 (-65.0, -45.0)		-52.9 (-56.0, -50.0)		-68.9 (-75.0, -62.0)		-64.2 (-74.0, -51.0)		-52.8 (-67.0, -38.0)		-44.4 (-58.0, -26.0)	
Net drift of mortality†, % per year	-2.90 (-3.03, -2.78)		-3.80 (-4.12, -3.48)		-5.16 (-5.38, -4.93)		-3.71 (-3.88, -3.55)		-2.77 (-2.89, -2.64)		-2.38 (-2.49, -2.28)	
DALY												
Number*, n×1,000	5747.6 (4827.1, 6847.8)	4833.5 (4122.0, 5729.1)	220.0 (210.8, 231.5)	167.8 (154.4, 183.2)	866.6 (788.8, 955.3)	446.1 (386.7, 509.8)	1784.9 (1532.4, 2032.3)	1289.7 (1070.0, 1529.5)	2036.1 (1556.9, 2720.0)	1904.8 (1517.2, 2426.3)	837.3 (592.7, 1178.0)	1022.0 (797.6, 1286.2)

Percentage of global, %	-15.9 (-30.0, 0)		-23.7 (-29.0, -18.0)		-48.5 (-57.0, -39.0)		-27.8 (-43.0, -6.00)		-6.45 (-27.0, 18.0)		22.1 (-2.00, 57.0)	
Percent change of DALY 1990–2019, %	100	100	3.83	3.47	15.1	9.23	31.1	26.7	35.4	39.4	14.6	21.1
All-age DALY rate												
Rate, per 100,000	213.4 (179.2, 254.2)	124.5 (106.2, 147.6)	54.3 (52.1, 57.2)	33.2 (30.5, 36.2)	151.5 (137.9, 167.0)	62.4 (54.1, 71.4)	204.5 (175.6, 232.8)	107.0 (88.7, 126.8)	353.4 (270.2, 472.1)	214.9 (171.1, 273.7)	314.3 (222.5, 442.2)	180.5 (140.9, 227.2)
Percent change of rate 1990–2019, %	-41.6 (-51.0, -30.0)		-38.9 (-43.0, -34.0)		-58.8 (-66.0, -51.0)		-47.7 (-59.0, -32.0)		-39.2 (-52.0, -23.0)		-42.6 (-54.0, -26.0)	
Age-standardized DALY rate												
Rate, per 100,000	253.7 (211.8, 304.0)	123.5 (105.8, 145.9)	50.4 (48.2, 53.2)	21.3 (19.6, 23.3)	165.9 (151.1, 182.9)	52.6 (45.5, 60.6)	274.6 (235.8, 313.4)	106.9 (88.9, 126.4)	488.0 (362.2, 666.2)	241.0 (192.0, 311.3)	453.8 (303.5, 673.9)	255.5 (193.6, 342.3)
Percent change of rate 1990–2019, %	-51.3 (-60.0, -42.0)		-57.7 (-61.0, -54.0)		-68.3 (-74.0, -62.0)		-61.1 (-70.0, -49.0)		-56.0 (-62.0, -37.0)		-43.7 (-55.0, -27.0)	
Net drift of DALY†, % per year	-2.47 (-2.58, -2.36)		-3.29 (-3.39, -3.19)		-4.26 (-4.39, -4.14)		-3.10 (-3.24, -2.96)		-2.46 (-2.58, -2.34)		-2.01 (-2.14, -1.88)	
Female												
Deaths												
Number*, n×1,000	210.1 (185.7, 238.3)	173.9 (140.7, 208.1)	18.4 (17.0, 19.2)	16.0 (13.0, 18.0)	41.7 (37.5, 46.4)	22.3 (19.3, 25.0)	75.8 (65.3, 89.2)	45.8 (37.7, 54.7)	54.7 (44.9, 64.5)	64.0 (44.1, 86.9)	19.4 (14.5, 23.9)	25.8 (19.4, 33.4)
Percentage of global, %	-17.2 (-34.0, 3.00)		-13.2 (-24.0, -5.00)		-46.6 (-55.0, -37.0)		-39.6 (-53.0, -22.0)		17.1 (-14.0, 57.0)		32.6 (0, 75.0)	
Percent change of deaths 1990–2019, %	100	100	8.75	9.17	19.8	12.8	36.1	26.3	26.0	36.8	9.25	14.8
All-age mortality rate												

Rate, per 100,000	7.91 (6.99, 8.97)	4.51 (3.65, 5.40)	4.41 (4.07, 4.61)	3.14 (2.56, 3.55)	7.21 (6.49, 8.03)	3.11 (2.70, 3.49)	8.98 (7.73, 10.57)	3.85 (3.17, 4.59)	9.88 (8.12, 11.66)	7.30 (5.03, 9.91)	7.43 (5.54, 9.11)	4.58 (3.45, 5.94)
Percent change of rate 1990–2019, %	-43.0 (-55.0, -29.0)		-28.7 (-38.0, -22.0)		-56.8 (-64.0, -49.0)		-57.2 (-67.0, -45.0)		-26.1 (-46.0, -1.00)		-38.3 (-53.0, -19.0)	
Age-standardized mortality rate												
Rate, per 100,000	9.65 (8.53, 11.0)	4.06 (3.29, 4.85)	2.93 (2.73, 3.06)	1.20 (1.01, 1.33)	7.11 (6.40, 7.92)	1.98 (1.73, 2.21)	14.5 (12.4, 17.2)	3.84 (3.15, 4.57)	17.0 (14.0, 20.2)	9.03 (6.27, 12.2)	14.2 (10.2, 18.1)	8.98 (6.82, 11.4)
Percent change of rate 1990–2019, %	-58.0 (-67.0, -48.0)		-59.2 (-63.0, -56.0)		-72.2 (-76.0, -67.0)		-73.6 (-80.0, -66.0)		-46.8 (-62.0, -27.0)		-36.5 (-55.0, -12.0)	
Net drift of mortality†, % per year	-3.37 (-3.51, -3.24)		-4.46 (-5.01, -3.90)		-5.98 (-6.28, -5.69)		-5.20 (-5.39, -5.00)		-2.68 (-2.83, -2.54)		-2.31 (-2.40, -2.22)	
DALY												
Number*, n×1,000	7420.7 (6565.3, 8275.3)	5840.4 (4814.0, 6963.0)	380.5 (361.1, 397.4)	243.6 (214.7, 268.5)	1247.3 (1125.4, 1377.6)	575.6 (508.9, 649.9)	2636.8 (2296.8, 3010.1)	1483.4 (1272.2, 1734.7)	2243.5 (1827.1, 2631.9)	2363.8 (1737.8, 3106.0)	908.9 (694.0, 1103.0)	1169.9 (911.9, 1477.3)
Percentage of global, %	-21.3 (-34.0, -5.00)		-36.0 (-41.0, -31.0)		-53.9 (-60.0, -47.0)		-43.7 (-54.0, -30.0)		5.36 (-17.0, 33.0)		28.7 (4.00, 58.0)	
Percent change of DALY 1990–2019, %	100	100	5.13	4.17	16.8	9.86	35.5	25.4	30.2	40.5	12.3	20.0
All-age DALY rate												
Rate, per 100,000	279.4 (247.2, 311.6)	151.4 (124.8, 180.6)	91.2 (86.6, 95.3)	48.0 (42.3, 52.9)	215.7 (194.6, 238.2)	80.4 (71.1, 90.8)	312.4 (272.2, 356.7)	124.6 (106.9, 145.7)	405.4 (330.1, 475.5)	269.4 (198.0, 354.0)	347.3 (265.2, 421.4)	208.0 (162.1, 262.6)
Percent change of rate 1990–2019, %	-45.8 (-55.0, -35.0)		-47.4 (-52.0, -44.0)		-62.7 (-68.0, -57.0)		-61.0 (-67.0, -51.0)		-33.6 (-48.0, -16.0)		-41.0 (-51.0, -27.0)	
Age-standardized DALY rate												

Rate, per 100,000	312.7 (276.9, 349.8)	142.0 (117.5, 168.6)	67.0 (63.7, 69.9)	23.9 (21.7, 26.3)	209.8 (189.5, 231.4)	59.0 (51.6, 67.6)	406.5 (352.4, 469.9)	117.0 (100.6, 136.6)	540.3 (445.8, 630.1)	291.4 (212.7, 383.5)	483.6 (366.0, 586.9)	295.5 (230.1, 378.5)
Percent change of rate 1990–2019, %	-54.6 (-62.0, -45.0)		-64.3 (-67.0, -62.0)		-71.9 (-76.0, -67.0)		-71.2 (-77.0, -64.0)		-46.1 (-58.0, -30.0)		-38.9 (-52.0, -22.0)	
Net drift of DALY†, % per year	-2.84 (-2.96, -2.72)		-3.93 (-4.14, -3.71)		-4.76 (-4.92, -4.60)		-4.32 (-4.50, -4.15)		-2.36 (-2.49, -2.22)		-1.88 (-2.01, -1.76)	

All-age mortality=crude mortality rate.

Age-standardized mortality rate is computed by direct standardization with global standard population in GBD 2019.

†Net drifts are estimates derived from the age-period-cohort model and denotes overall annual percentage change in mortality, which captures the contribution of the effects from calendar time and successive birth cohorts

*Parentheses for all GBD health estimate indicate 95% uncertainty intervals; parentheses for net drift indicate 95% confidence intervals.

Abbreviation: DALYs, disability-adjusted life-years; GBD, Global Burden of Diseases, Injuries, and Risk Factors Study; RHD, rheumatic heart disease; SDI, Socio-demographic Index.

Supplementary Table 2. The temporal change of deaths from RHD by SDI quintiles in 204 countries and territories during 1990-2019.

	Deaths		All-age mortality		Age-standardized mortality		
	Number in 2019, n×1,000	Percent change 1990-2019, %	Rate in 2019, Rate per 100,000	Percent change 1990-2019, %	Rate in 2019, Rate per 100,000	Percent change 1990-2019, %	Net drift, % per year
High SDI							
Denmark	87.3 (68.7, 106.8)	-66.2 (-73.0, -58.0)	1.50 (1.18, 1.84)	-70.0 (-76.0, -63.0)	0.69 (0.54, 0.83)	-77.4 (-82.0, -72.0)	-4.83 (-10.6, 1.25)
Puerto Rico	31.5 (23.4, 41.3)	-33.0 (-51.0, -10.0)	0.90 (0.66, 1.17)	-31.3 (-49.0, -8.00)	0.46 (0.34, 0.60)	-65.8 (-75.0, -54.0)	-3.98 (-7.01, -0.86)
Switzerland	144.5 (112.8, 174.9)	-56.0 (-64.0, -46.0)	1.65 (1.29, 1.99)	-65.6 (-72.0, -58.0)	0.66 (0.53, 0.79)	-77.7 (-82.0, -73.0)	-6.06 (-10.7, -1.21)
Russian Federation	2849.1 (2418.8, 3304.3)	-72.0 (-76.0, -67.0)	1.94 (1.65, 2.25)	-71.2 (-75.0, -66.0)	1.24 (1.05, 1.44)	-77.8 (-81.0, -74.0)	-7.20 (-7.80, -6.59)
Germany	4603.8 (3770.0, 5513.5)	4.34 (-15.0, 26.0)	5.42 (4.44, 6.49)	-1.77 (-20.0, 19.0)	1.98 (1.66, 2.35)	-41.6 (-52.0, -30.0)	-3.74 (-4.72, -2.76)
Finland	54.7 (43.8, 67.1)	-61.2 (-69.0, -53.0)	0.99 (0.79, 1.21)	-64.9 (-72.0, -57.0)	0.39 (0.32, 0.48)	-79.9 (-84.0, -75.0)	-5.00 (-10.9, 1.25)
France	2566.1 (2004.0, 3157.8)	18.7 (-8.00, 47.0)	3.88 (3.03, 4.77)	3.53 (-20.0, 28.0)	1.44 (1.15, 1.74)	-42.5 (-54.0, -30.0)	-2.67 (-3.61, -1.73)
Czechia	292.2 (232.3, 363.9)	-59.4 (-68.0, -49.0)	2.75 (2.18, 3.42)	-67.0 (-69.0, -51.0)	1.35 (1.07, 1.68)	-74.8 (-80.0, -69.0)	-6.15 (-8.68, -3.56)
United Kingdom	1297.2 (1147.7, 1401.6)	-52.7 (-56.0, -49.0)	1.93 (1.71, 2.09)	-59.6 (-63.0, -56.0)	0.93 (0.83, 1.01)	-68.7 (-71.0, -66.0)	-4.56 (-5.70, -3.41)
San Marino	1.69 (1.15, 2.36)	67.5 (9.00, 149.0)	5.11 (3.47, 7.14)	19.2 (-22.0, 77.0)	2.19 (1.48, 3.10)	-33.0 (-55.0, 6.00)	-0.96 (-38.1, 58.4)
Brunei Darussalam	6.29 (5.32, 7.32)	39.4 (11.0, 85.0)	1.44 (1.22, 1.67)	-17.5 (-35.0, 9.00)	2.65 (2.28, 3.04)	-42.4 (-54.0, -26.0)	-2.72 (-8.67, 3.63)

Kuwait	11.2 (8.68, 14.6)	-26.6 (-45.0, -4.00)	0.25 (0.20, 0.33)	-78.0 (-78.0, -62.0)	0.43 (0.33, 0.55)	-77.6 (-83.0, -71.0)	-5.03 (-8.17, -1.78)
Ireland	62.4 (49.8, 76.6)	-32.4 (-47.0, -14.0)	1.27 (1.01, 1.56)	-54.0 (-61.0, -37.0)	0.81 (0.65, 0.99)	-65.1 (-73.0, -56.0)	-3.87 (-8.60, 1.10)
Republic of Korea	314.9 (257.7, 378.9)	60.0 (-17.0, 24.0)	0.59 (0.48, 0.71)	-16.5 (-31.0, 3.00)	0.38 (0.31, 0.46)	-67.1 (-73.0, -59.0)	-5.86 (-7.13, -4.58)
Taiwan (Province of China)	246.3 (180.7, 323.1)	-68.0 (-72.0, -48.0)	1.04 (0.77, 1.37)	-66.2 (-75.0, -55.0)	0.63 (0.46, 0.82)	-86.7 (-90.0, -82.0)	-7.68 (-9.10, -6.23)
Luxembourg	11.6 (9.14, 14.3)	73.0 (-21.0, 27.0)	1.88 (1.48, 2.31)	-37.9 (-52.0, -22.0)	1.05 (0.84, 1.29)	-52.0 (-63.0, -40.0)	-3.54 (-15.4, 10.01)
Singapore	25.6 (20.7, 31.3)	-54.6 (-64.0, -42.0)	0.45 (0.36, 0.55)	-75.6 (-81.0, -69.0)	0.34 (0.28, 0.42)	-85.3 (-88.0, -82.0)	-7.42 (-10.3, -4.44)
Slovenia	103.1 (75.5, 140.6)	-1.77 (-33.0, 38.0)	4.97 (3.64, 6.78)	-6.66 (-37.0, 31.0)	2.08 (1.54, 2.82)	-52.9 (-68.0, -34.0)	-4.07 (-9.78, 1.99)
United States of America	6001.8 (5256.5, 6595.7)	-21.9 (-27.0, -16.0)	1.83 (1.60, 2.01)	-39.6 (-44.0, -35.0)	1.00 (0.89, 1.09)	-57.8 (-60.0, -55.0)	-4.13 (-4.57, -3.70)
Australia	466.8 (388.4, 545.6)	9.02 (-9.00, 28.0)	1.90 (1.58, 2.22)	-25.2 (-37.0, -12.0)	1.03 (0.87, 1.21)	-54.3 (-61.0, -46.0)	-3.22 (-4.61, -1.80)
Iceland	3.04 (2.40, 3.67)	-1.29 (-21.0, 22.0)	0.88 (0.70, 1.06)	-27.3 (-42.0, -10.0)	0.49 (0.39, 0.58)	-53.4 (-63.0, -42.0)	-2.87 (-19.8, 17.7)
Canada	784.0 (627.8, 947.8)	17.1 (-4.00, 43.0)	2.15 (1.72, 2.60)	-12.6 (-29.0, 6.00)	1.05 (0.85, 1.25)	-50.0 (-59.0, -40.0)	-2.95 (-4.56, -1.30)
Japan	4759.7 (3530.6, 5535.1)	58.4 (28.0, 79.0)	3.72 (2.76, 4.33)	56.0 (26.0, 77.0)	0.88 (0.68, 1.00)	-55.5 (-62.0, -51.0)	-4.23 (-5.11, -3.34)
Slovakia	96.2 (74.8, 121.9)	-26.8 (-45.0, -5.00)	1.77 (1.37, 2.24)	-28.9 (-47.0, -7.00)	1.06 (0.83, 1.34)	-52.4 (-64.0, -38.0)	-3.49 (-6.50, -0.38)
New Zealand	147.4 (124.8, 170.7)	-1.47 (-18.0, 16.0)	3.28 (2.78, 3.80)	-25.1 (-37.0, -12.0)	1.92 (1.65, 2.20)	-51.7 (-59.0, -44.0)	-3.50 (-5.45, -1.52)
Estonia	22.9 (16.3, 32.0)	-76.9 (-84.0, -68.0)	1.75 (1.25, 2.44)	-72.4 (-81.0, -61.0)	0.96 (0.67, 1.33)	-85.0 (-87.0, -72.0)	-7.33 (-14.6, 0.54)

Belgium	489.1 (379.3, 598.8)	174.6 (119.0, 240.0)	4.28 (3.32, 5.24)	140.0 (91.0, 197.0)	1.66 (1.31, 2.01)	43.5 (15.0, 76.0)	-0.54 (-4.10, 3.15)
Qatar	6.13 (4.46, 8.25)	42.9 (-22.0, 146.0)	0.21 (0.16, 0.29)	-77.8 (-88.0, -62.0)	0.83 (0.64, 1.05)	-72.2 (-86.0, -53.0)	-6.51 (-10.7, -2.13)
Saudi Arabia	234.5 (156.7, 324.4)	-8.37 (-60.0, 66.0)	0.66 (0.44, 0.91)	-58.9 (-82.0, -25.0)	0.91 (0.64, 1.19)	-69.8 (-87.0, -47.0)	-4.52 (-5.21, -3.82)
Latvia	38.9 (29.4, 52.0)	-83.3 (-88.0, -77.0)	2.03 (1.54, 2.72)	-76.9 (-83.0, -69.0)	1.13 (0.84, 1.53)	-83.3 (-88.0, -77.0)	-7.31 (-12.7, -1.63)
Austria	317.8 (260.3, 374.5)	-13.0 (-24.0, 6.00)	3.56 (2.92, 4.20)	-21.8 (-34.0, -8.00)	1.45 (1.20, 1.69)	-52.0 (-58.0, -41.0)	-3.89 (-6.73, -0.98)
Andorra	1.49 (1.12, 1.95)	96.4 (29.0, 204.0)	1.80 (1.35, 2.35)	27.9 (-16.0, 98.0)	0.97 (0.72, 1.27)	-45.4 (-64.0, -17.0)	-2.38 (-33.9, 44.1)
Netherlands	301.0 (242.0, 367.5)	48.2 (20.0, 84.0)	1.75 (1.41, 2.14)	28.9 (4.00, 60.0)	0.80 (0.65, 0.97)	-21.6 (-36.0, -3.00)	-2.86 (-5.77, 0.15)
United Arab Emirates	142.9 (83.6, 231.9)	219.3 (39.0, 613.0)	1.55 (0.90, 2.51)	-35.3 (-72.0, 44.0)	3.45 (2.08, 5.28)	-64.1 (-83.0, -27.0)	-3.35 (-4.66, -2.02)
Lithuania	69.2 (52.2, 89.6)	-78.7 (-84.0, -72.0)	2.48 (1.87, 3.21)	-72.0 (-79.0, -64.0)	1.36 (1.02, 1.76)	-81.5 (-86.0, -76.0)	-6.78 (-11.5, -1.78)
Bermuda	0.51 (0.40, 0.67)	-51.0 (-63.0, -36.0)	0.80 (0.63, 1.04)	-54.5 (-66.0, -41.0)	0.45 (0.36, 0.58)	-73.1 (-80.0, -65.0)	-5.09 (-23.7, 18.0)
Cyprus	47.5 (39.7, 57.9)	-7.62 (-35.0, 19.0)	3.62 (3.02, 4.41)	-45.3 (-62.0, -29.0)	2.77 (2.33, 3.38)	-63.9 (-74.0, -54.0)	-4.61 (-12.3, 3.76)
Guam	4.23 (3.48, 5.17)	-2.62 (-25.0, 25.0)	2.48 (2.04, 3.03)	-22.0 (-40.0, 1.00)	2.37 (1.96, 2.88)	-55.5 (-65.0, -43.0)	-2.09 (-6.70, 2.76)
Norway	85.5 (72.3, 96.0)	-42.0 (-46.0, -34.0)	1.60 (1.35, 1.80)	-52.5 (-57.0, -48.0)	0.75 (0.64, 0.84)	-66.0 (-64.0, -57.0)	-4.99 (-10.7, 1.14)
Sweden	193.2 (151.1, 236.5)	-37.0 (-48.0, -23.0)	1.89 (1.48, 2.31)	-47.0 (-57.0, -35.0)	0.75 (0.59, 0.91)	-59.5 (-66.0, -51.0)	-3.74 (-7.57, 0.23)
Monaco	0.61 (0.47, 0.73)	-6.81 (-29.0, 22.0)	1.62 (1.26, 1.95)	-24.5 (-43.0, -1.00)	0.55 (0.42, 0.66)	-36.1 (-52.0, -16.0)	-1.61 (-51.9, 101.1)
High-middle SDI							
Dominica	1.52 (1.19, 1.96)	-43.0 (-54.0, -22.0)	2.22 (1.73, 2.86)	-35.6 (-51.0, -16.0)	1.84 (1.43, 2.38)	-51.1 (-63.0, -36.0)	-2.60 (-11.8, 7.61)

Croatia	102.8 (77.5, 133.2)	-59.7 (-7.00, -46.0)	2.42 (1.82, 3.14)	-53.5 (-65.0, -38.0)	1.11 (0.85, 1.43)	-73.2 (-80.0, -65.0)	-5.20 (-9.03, -1.22)
Ukraine	1041.8 (818.8, 1313.7)	-36.8 (-57.0, -9.00)	2.37 (1.86, 2.98)	-24.4 (-48.0, 9.00)	1.51 (1.19, 1.91)	-34.9 (-54.0, -6.00)	-1.37 (-2.28, -0.45)
Hungary	269.6 (213.6, 332.3)	-78.0 (-77.0, -64.0)	2.79 (2.21, 3.43)	-68.6 (-75.0, -61.0)	1.34 (1.06, 1.64)	-79.5 (-84.0, -75.0)	-6.95 (-9.09, -4.76)
Argentina	1597.8 (1291.1, 1923.9)	-19.7 (-35.0, -2.00)	3.54 (2.86, 4.26)	-41.1 (-52.0, -28.0)	2.87 (2.34, 3.46)	-57.2 (-65.0, -48.0)	-4.90 (-5.58, -4.22)
Saint Kitts and Nevis	0.64 (0.43, 0.87)	-52.5 (-69.0, -33.0)	1.07 (0.72, 1.47)	-67.0 (-79.0, -53.0)	1.01 (0.70, 1.34)	-73.9 (-82.0, -64.0)	-4.86 (-16.7, 8.67)
North Macedonia	37.2 (28.7, 47.8)	-51.9 (-67.0, -35.0)	1.73 (1.33, 2.22)	-54.9 (-69.0, -39.0)	1.26 (0.98, 1.59)	-69.7 (-79.0, -59.0)	-5.89 (-8.87, -2.80)
Kazakhstan	368.4 (276.7, 471.8)	-66.0 (-74.0, -56.0)	2.00 (1.50, 2.57)	-69.7 (-77.0, -61.0)	1.99 (1.51, 2.53)	-73.8 (-80.0, -66.0)	-6.95 (-7.70, -6.19)
Romania	426.8 (339.1, 528.0)	-73.3 (-79.0, -67.0)	2.22 (1.76, 2.74)	-67.5 (-74.0, -59.0)	1.18 (0.94, 1.47)	-81.0 (-84.0, -75.0)	-7.55 (-8.86, -6.22)
Chile	189.1 (156.9, 228.3)	-57.9 (-66.0, -49.0)	1.04 (0.86, 1.25)	-69.3 (-75.0, -63.0)	0.81 (0.67, 0.97)	-81.6 (-85.0, -78.0)	-6.68 (-7.89, -5.46)
Poland	985.2 (825.4, 1159.5)	-73.7 (-78.0, -69.0)	2.56 (2.15, 3.02)	-73.9 (-78.0, -69.0)	1.40 (1.17, 1.64)	-83.9 (-86.0, -81.0)	-8.14 (-9.09, -7.17)
Montenegro	11.7 (8.99, 14.5)	-52.0 (-34.0, 41.0)	1.88 (1.45, 2.34)	34.0 (-34.0, 42.0)	1.26 (0.97, 1.56)	-33.4 (-56.0, -7.00)	-2.86 (-10.8, 5.76)
Greece	165.9 (133.2, 201.8)	-24.5 (-40.0, -7.00)	1.60 (1.29, 1.95)	-24.1 (-39.0, -6.00)	0.57 (0.47, 0.69)	-62.8 (-70.0, -55.0)	-3.77 (-7.34, -0.07)
Malaysia	231.1 (170.5, 297.7)	-52.1 (-66.0, -36.0)	0.74 (0.54, 0.95)	-73.0 (-81.0, -64.0)	0.85 (0.62, 1.08)	-79.7 (-85.0, -73.0)	-5.41 (-6.08, -4.74)
United States Virgin Islands	1.20 (0.95, 1.47)	-22.0 (-42.0, 4.00)	1.15 (0.91, 1.41)	-25.0 (-41.0, 6.00)	0.79 (0.62, 0.99)	-51.9 (-65.0, -36.0)	-3.37 (-16.4, 11.7)

Lebanon	43.4 (20.1, 66.4)	-27.9 (-73.0, 16.0)	0.84 (0.39, 1.28)	-54.4 (-83.0, -27.0)	0.84 (0.39, 1.28)	-68.4 (-88.0, -49.0)	-4.53 (-6.23, -2.80)
Serbia	145.5 (115.3, 180.9)	-43.8 (-65.0, -19.0)	1.66 (1.32, 2.07)	-39.7 (-62.0, -13.0)	0.94 (0.75, 1.17)	-62.9 (-76.0, -47.0)	-5.74 (-8.77, -2.62)
Antigua and Barbuda	1.00 (0.83, 1.20)	-19.5 (-35.0, 0)	1.13 (0.94, 1.36)	-44.7 (-55.0, -31.0)	1.01 (0.84, 1.21)	-55.1 (-63.0, -44.0)	-3.41 (-14.1, 8.54)
Israel	197.8 (165.9, 229.6)	55.5 (30.0, 85.0)	2.12 (1.78, 2.47)	-17.1 (-31.0, -2.00)	1.63 (1.37, 1.89)	-44.0 (-50.0, -29.0)	-3.07 (-4.94, -1.16)
Bulgaria	212.9 (155.3, 285.6)	-72.7 (-81.0, -63.0)	3.07 (2.24, 4.12)	-65.9 (-76.0, -53.0)	1.71 (1.24, 2.32)	-73.7 (-81.0, -64.0)	-5.78 (-7.30, -4.23)
Italy	2734.9 (2328.6, 3045.2)	2.22 (-10.0, 11.0)	4.53 (3.86, 5.05)	-3.74 (-15.0, 4.00)	1.59 (1.38, 1.74)	-48.6 (-54.0, -45.0)	-4.31 (-5.07, -3.54)
Barbados	4.36 (3.33, 5.54)	-23.9 (-43.0, 0)	1.46 (1.12, 1.86)	-35.2 (-52.0, -15.0)	1.03 (0.79, 1.3)	-51.9 (-64.0, -36.0)	-2.95 (-10.1, 4.81)
Trinidad and Tobago	14.8 (10.5, 20.4)	-48.2 (-65.0, -28.0)	1.06 (0.75, 1.47)	-55.1 (-69.0, -37.0)	0.89 (0.63, 1.23)	-69.7 (-79.0, -58.0)	-4.72 (-7.28, -2.10)
Bahamas	3.82 (2.77, 5.06)	2.87 (-29.0, 45.0)	1.01 (0.73, 1.34)	-30.0 (-52.0, -2.00)	0.93 (0.69, 1.23)	-49.8 (-65.0, -30.0)	-2.68 (-8.68, 3.72)
Bosnia and Herzegovina	34.6 (27.2, 44.0)	-63.0 (-72.0, -51.0)	1.05 (0.83, 1.33)	-49.1 (-61.0, -33.0)	0.60 (0.48, 0.76)	-75.1 (-81.0, -67.0)	-6.29 (-10.3, -2.13)
Malta	9.99 (8.04, 12.3)	6.82 (-17.0, 35.0)	2.28 (1.83, 2.79)	-9.85 (-30.0, 14.0)	1.02 (0.83, 1.24)	-56.3 (-66.0, -45.0)	-2.20 (-13.7, 10.8)
Jordan	29.4 (23.3, 36.3)	16.2 (-24.0, 71.0)	0.25 (0.20, 0.31)	-62.3 (-75.0, -44.0)	0.45 (0.35, 0.56)	-71.8 (-82.0, -59.0)	-5.34 (-7.35, -3.29)
Belarus	258.0 (178.6, 365.0)	-70.0 (-80.0, -57.0)	2.72 (1.88, 3.84)	-66.9 (-78.0, -52.0)	1.70 (1.19, 2.40)	-74.8 (-83.0, -64.0)	-6.24 (-9.12, -3.26)
Seychelles	0.89 (0.74, 1.05)	-68.6 (-74.0, -62.0)	0.87 (0.73, 1.03)	-77.6 (-81.0, -73.0)	0.84 (0.71, 0.99)	-82.2 (-85.0, -79.0)	-5.33 (-15.5, 6.02)
Portugal	304.0 (251.6, 364.8)	-35.0 (-44.0, -16.0)	2.85 (2.36, 3.42)	-33.8 (-46.0, -20.0)	1.14 (0.96, 1.35)	-66.8 (-73.0, -60.0)	-6.20 (-8.34, -4.00)
Cook Islands	0.45 (0.35, 0.56)	-34.5 (-53.0, -9.00)	2.49 (1.96, 3.11)	-38.0 (-50.0, -4.00)	2.11 (1.62, 2.67)	-56.6 (-69.0, -39.0)	-2.85 (-17.6, 14.5)

Libya	55.7 (37.3, 77.6)	-33.5 (-63.0, 30.0)	0.83 (0.55, 1.15)	-58.2 (-77.0, -18.0)	1.05 (0.74, 1.43)	-58.1 (-76.0, -29.0)	-3.47 (-4.86, -2.07)
American Samoa	2.69 (2.16, 3.37)	9.83 (-21.0, 53.0)	4.84 (3.89, 6.07)	-4.17 (-31.0, 33.0)	5.69 (4.56, 7.13)	-36.3 (-54.0, -12.0)	-1.49 (-7.51, 4.92)
Turkey	504.7 (395.6, 622.1)	-22.3 (-52.0, 12.0)	0.62 (0.49, 0.76)	-42.9 (-65.0, -18.0)	0.59 (0.46, 0.73)	-66.2 (-79.0, -52.0)	-4.63 (-5.32, -3.95)
Bahrain	8.25 (6.48, 10.5)	82.8 (26.0, 158.0)	0.57 (0.45, 0.73)	-35.6 (-56.0, -9.00)	1.12 (0.87, 1.41)	-53.1 (-68.0, -35.0)	-4.43 (-8.61, -0.06)
Spain	2492.1 (1988.0, 3056.1)	-7.39 (-24.0, 13.0)	5.42 (4.32, 6.64)	-22.0 (-36.0, -5.00)	2.10 (1.70, 2.54)	-58.7 (-66.0, -50.0)	-5.50 (-6.64, -4.34)
Oman	7.64 (6.22, 9.67)	-38.5 (-65.0, 1.00)	0.17 (0.14, 0.21)	-73.9 (-85.0, -57.0)	0.60 (0.49, 0.72)	-69.6 (-84.0, -45.0)	-5.46 (-9.19, -1.58)
Greenland	0.87 (0.71, 1.06)	-39.2 (-54.0, -21.0)	1.56 (1.27, 1.89)	-39.8 (-54.0, -22.0)	1.54 (1.27, 1.85)	-71.3 (-78.0, -63.0)	-5.54 (-28.8, 25.2)
Niue	0.09 (0.06, 0.13)	-54.6 (-70.0, -34.0)	5.51 (3.30, 7.50)	-36.9 (-58.0, -8.00)	4.81 (2.92, 6.67)	-47.5 (-66.0, -22.0)	-2.43 (-28.4, 32.9)
Northern Mariana Islands	1.25 (1.01, 1.54)	-4.54 (-32.0, 39.0)	2.95 (2.38, 3.64)	1.98 (-28.0, 49.0)	2.84 (2.31, 3.48)	-38.9 (-55.0, -13.0)	-1.07 (-10.2, 9.02)
Palau	0.69 (0.53, 0.87)	-7.48 (-41.0, 41.0)	3.85 (2.94, 4.86)	-29.0 (-49.0, 21.0)	3.58 (2.77, 4.45)	-44.9 (-64.0, -17.0)	-1.7- (-12.9, 10.9)
Middle SDI							
Costa Rica	50.1 (36.6, 67.2)	2.70 (-27.0, 41.0)	1.06 (0.78, 1.42)	-33.8 (-53.0, -9.00)	0.97 (0.71, 1.30)	-59.2 (-71.0, -44.0)	-3.46 (-5.17, -1.70)
Iran (Islamic Republic of)	1029.6 (885.9, 1164.9)	-9.34 (-40.0, 17.0)	1.22 (1.05, 1.38)	-37.0 (-58.0, -19.0)	1.48 (1.26, 1.67)	-59.4 (-76.0, -46.0)	-4.02 (-4.37, -3.66)
Gabon	18.6 (13.1, 25.3)	-34.4 (-56.0, -1.00)	1.07 (0.75, 1.44)	-62.9 (-75.0, -44.0)	1.87 (1.35, 2.52)	-62.0 (-74.0, -45.0)	-4.27 (-6.80, -1.67)
Armenia	112.4 (89.5, 137.4)	-45.8 (-57.0, -32.0)	3.72 (2.97, 4.55)	-38.7 (-51.0, -23.0)	2.81 (2.25, 3.42)	-60.0 (-68.0, -50.0)	-4.68 (-6.86, -2.45)
Azerbaijan	181.1 (138.4, 234.3)	-23.4 (-42.0, 0)	1.76 (1.35, 2.28)	-45.3 (-59.0, -29.0)	2.06 (1.58, 2.69)	-50.0 (-64.0, -35.0)	-3.70 (-4.62, -2.77)
Grenada	2.58 (2.08, 3.18)	-43.7 (-56.0, -28.0)	2.50 (2.02, 3.08)	-53.3 (-63.0, -40.0)	2.36 (1.92, 2.88)	-63.2 (-71.0, -53.0)	-3.39 (-9.95, 3.66)

Iraq	357.7 (272.8, 460.0)	-27.5 (-57.0, 14.0)	0.85 (0.65, 1.09)	-69.7 (-82.0, -52.0)	1.53 (1.19, 1.90)	-71.5 (-83.0, -57.0)	-5.93 (-6.40, -5.45)
Tokelau	0.08 (0.06, 0.12)	-55.8 (-70.0, -35.0)	5.99 (4.29, 8.16)	-47.2 (-64.0, -22.0)	6.51 (4.66, 8.84)	-55.8 (-69.0, -34.0)	-2.72 (-28.7, 32.7)
Sri Lanka	188.4 (130.2, 253.5)	-47.4 (-64.0, -28.0)	0.86 (0.60, 1.16)	-58.5 (-71.0, -43.0)	0.83 (0.58, 1.11)	-72.8 (-82.0, -63.0)	-5.32 (-6.10, -4.53)
China	69781.7 (57365.3, 81360.9)	-47.4 (-61.0, -32.0)	4.91 (4.03, 5.72)	-56.2 (-67.0, -44.0)	4.04 (3.32, 4.69)	-77.7 (-83.0, -71.0)	-7.08 (-7.39, -6.77)
Georgia	366.8 (291.5, 445.9)	55.3 (21.0, 95.0)	10.0 (7.95, 12.2)	133.5 (82.0, 193.0)	6.03 (4.84, 7.33)	45.8 (14.0, 81.0)	0.37 (-0.49, 1.25)
Botswana	34.8 (18.5, 51.4)	-14.3 (-47.0, 35.0)	1.49 (0.79, 2.20)	-52.3 (-70.0, -25.0)	2.25 (1.15, 3.26)	-61.4 (-76.0, -41.0)	-3.71 (-5.23, -2.16)
Thailand	463.1 (341.5, 613.0)	-76.0 (-84.0, -67.0)	0.66 (0.49, 0.87)	-85.0 (-87.0, -73.0)	0.50 (0.37, 0.66)	-89.8 (-93.0, -86.0)	-9.58 (-10.1, -9.05)
Uruguay	71.5 (58.6, 83.9)	-14.4 (-30.0, 4.00)	2.08 (1.71, 2.44)	-21.8 (-36.0, -5.00)	1.23 (1.01, 1.44)	-45.6 (-55.0, -34.0)	-2.52 (-5.42, 0.47)
South Africa	649.5 (580.6, 716.2)	-28.6 (-39.0, -15.0)	1.17 (1.04, 1.29)	-52.7 (-60.0, -43.0)	1.41 (1.27, 1.54)	-57.3 (-63.0, -49.0)	-4.63 (-5.19, -4.07)
Saint Lucia	3.61 (2.82, 4.57)	-24.7 (-43.0, -2.00)	2.07 (1.61, 2.62)	-47.0 (-55.0, -23.0)	1.74 (1.37, 2.20)	-66.3 (-74.0, -56.0)	-3.87 (-10.1, 2.75)
Mexico	1036.3 (878.9, 1205.1)	-43.7 (-53.0, -34.0)	0.83 (0.70, 0.96)	-61.5 (-68.0, -55.0)	0.88 (0.75, 1.03)	-76.3 (-80.0, -72.0)	-6.25 (-6.61, -5.90)
Fiji	65.5 (50.4, 84.6)	-11.2 (-38.0, 32.0)	7.19 (5.54, 9.29)	-26.0 (-49.0, 10.0)	8.76 (6.9, 11.18)	-42.6 (-59.0, -16.0)	-1.86 (-2.97, -0.73)
Jamaica	33.0 (24.1, 43.3)	-47.1 (-62.0, -29.0)	1.17 (0.86, 1.54)	-55.5 (-68.0, -40.0)	1.08 (0.78, 1.42)	-62.5 (-73.0, -49.0)	-3.60 (-5.29, -1.89)
Indonesia	1297.6 (1108.3, 1500.3)	-49.1 (-60.0, -34.0)	0.50 (0.43, 0.58)	-63.6 (-72.0, -53.0)	0.67 (0.57, 0.76)	-69.4 (-77.0, -60.0)	-4.98 (-5.21, -4.75)

Egypt	1271.7 (773.0, 1873.5)	-47.9 (-68.0, -22.0)	1.28 (0.78, 1.89)	-77.0 (-82.0, -56.0)	1.93 (1.19, 2.80)	-66.6 (-80.0, -50.0)	-3.85 (-4.11, -3.59)
Paraguay	51.2 (36.1, 66.9)	22.7 (-18.0, 67.0)	0.74 (0.52, 0.96)	-28.4 (-52.0, -2.00)	0.87 (0.61, 1.13)	-45.0 (-63.0, -26.0)	-2.42 (-4.24, -0.56)
Saint Vincent and the Grenadines	2.20 (1.75, 2.72)	-24.5 (-42.0, -4.00)	1.95 (1.55, 2.40)	-26.6 (-43.0, -6.00)	1.74 (1.40, 2.14)	-59.0 (-62.0, -38.0)	-2.95 (-9.76, 4.38)
Algeria	432.7 (342.0, 535.9)	-22.7 (-46.0, 11.0)	1.03 (0.82, 1.28)	-53.3 (-67.0, -33.0)	1.39 (1.12, 1.69)	-65.1 (-76.0, -51.0)	-4.55 (-5.03, -4.07)
Suriname	6.17 (4.82, 7.61)	-14.0 (-30.0, 19.0)	1.07 (0.84, 1.32)	-39.9 (-53.0, -20.0)	1.03 (0.81, 1.26)	-52.9 (-63.0, -39.0)	-3.17 (-7.08, 0.90)
Panama	26.2 (18.7, 35.7)	-39.0 (-58.0, -14.0)	0.63 (0.45, 0.86)	-65.0 (-76.0, -51.0)	0.62 (0.44, 0.85)	-72.9 (-81.0, -62.0)	-5.20 (-6.90, -3.46)
Turkmenistan	111.0 (81.4, 147.5)	-25.8 (-46.0, 0)	2.18 (1.60, 2.90)	-45.9 (-60.0, -27.0)	2.35 (1.75, 3.11)	-59.8 (-70.0, -47.0)	-4.23 (-5.16, -3.29)
Peru	218.7 (148.3, 298.8)	-26.0 (-50.0, 17.0)	0.64 (0.44, 0.88)	-49.3 (-68.0, -25.0)	0.66 (0.45, 0.91)	-67.8 (-80.0, -52.0)	-4.68 (-5.46, -3.89)
Mauritius	12.6 (9.67, 16.4)	-59.9 (-71.0, -47.0)	0.99 (0.76, 1.28)	-65.4 (-75.0, -54.0)	0.82 (0.63, 1.06)	-77.7 (-84.0, -71.0)	-4.40 (-7.09, -1.63)
Albania	41.4 (29.6, 55.8)	-62.6 (-75.0, -48.0)	1.52 (1.09, 2.05)	-54.5 (-70.0, -37.0)	1.06 (0.76, 1.42)	-81.0 (-87.0, -73.0)	-5.35 (-7.19, -3.47)
Uzbekistan	1615.3 (1242.1, 2034.4)	26.9 (-2.00, 60.0)	4.80 (3.69, 6.04)	-21.1 (-39.0, 0)	5.79 (4.54, 7.15)	-32.8 (-47.0, -16.0)	-2.86 (-3.19, -2.52)
Brazil	2715.2 (2505.1, 2913.1)	-12.1 (-20.0, -4.00)	1.25 (1.16, 1.34)	-39.6 (-45.0, -34.0)	1.16 (1.07, 1.24)	-59.4 (-63.0, -55.0)	-3.73 (-3.93, -3.53)
Tonga	2.49 (1.93, 3.23)	-26.0 (-43.0, 11.0)	2.43 (1.88, 3.16)	-24.9 (-46.0, 5.00)	2.97 (2.31, 3.83)	-42.4 (-58.0, -20.0)	-1.80 (-7.69, 4.47)
Ecuador	185.1 (145.3, 236.0)	-15.7 (-35.0, 9.00)	1.05 (0.83, 1.34)	-52.0 (-63.0, -38.0)	1.31 (1.04, 1.65)	-66.7 (-75.0, -57.0)	-4.18 (-5.03, -3.32)
Samoa	12.4 (8.69, 17.0)	6.00 (-35.0, 53.0)	5.87 (4.11, 8.03)	-22.5 (-50.0, 19.0)	7.63 (5.43, 10.3)	-37.5 (-58.0, -7.00)	-1.32 (-4.09, 1.53)

Cuba	181.0 (135.7, 235.0)	-31.4 (-49.0, -9.00)	1.59 (1.19, 2.07)	-34.6 (-52.0, -13.0)	1.08 (0.82, 1.40)	-56.1 (-67.0, -42.0)	-3.57 (-4.65, -2.47)
Equatorial Guinea	8.54 (4.92, 13.2)	-53.5 (-76.0, -7.00)	0.60 (0.35, 0.93)	-85.9 (-93.0, -72.0)	1.72 (1.03, 2.61)	-79.3 (-89.0, -56.0)	-7.50 (-10.6, -4.31)
Colombia	204.6 (147.0, 275.7)	-54.1 (-67.0, -37.0)	0.43 (0.31, 0.58)	-68.7 (-78.0, -57.0)	0.38 (0.28, 0.52)	-82.8 (-88.0, -77.0)	-6.79 (-7.51, -6.06)
Tunisia	125.2 (81.6, 175.1)	-13.3 (-53.0, 35.0)	1.08 (0.70, 1.51)	-36.8 (-66.0, -2.00)	1.06 (0.70, 1.48)	-60.0 (-79.0, -38.0)	-3.94 (-4.98, -2.88)
Republic of Moldova	76.4 (61.5, 95.5)	-75.2 (-80.0, -69.0)	2.07 (1.67, 2.59)	-70.0 (-76.0, -62.0)	1.40 (1.13, 1.75)	-79.1 (-83.0, -74.0)	-6.43 (-8.82, -3.98)
Low-middle SDI							
Maldives	3.91 (3.12, 4.71)	-46.8 (-64.0, -22.0)	0.78 (0.63, 0.95)	-76.3 (-84.0, -65.0)	1.21 (0.98, 1.45)	-78.5 (-86.0, -68.0)	-7.01 (-11.4, -2.41)
Philippines	2357.5 (1818.8, 2838.6)	195.1 (90.0, 296.0)	2.10 (1.62, 2.53)	66.5 (7.00, 124.0)	2.33 (1.82, 2.79)	25.1 (-11.0, 70.0)	2.46 (2.18, 2.75)
Ghana	287.3 (222.6, 364.4)	13.0 (-20.0, 57.0)	0.91 (0.71, 1.16)	-46.2 (-62.0, -25.0)	1.80 (1.44, 2.25)	-52.9 (-67.0, -35.0)	-2.52 (-3.17, -1.86)
Myanmar	1090.7 (817.7, 1457.2)	-35.1 (-59.0, 0)	1.99 (1.50, 2.67)	-51.2 (-69.0, -25.0)	2.38 (1.84, 3.10)	-64.0 (-75.0, -39.0)	-3.84 (-4.10, -3.59)
Sudan	612.6 (385.2, 962.6)	-37.5 (-60.0, -6.00)	1.50 (0.94, 2.36)	-69.0 (-80.0, -54.0)	2.70 (1.76, 4.20)	-63.1 (-76.0, -44.0)	-3.59 (-3.97, -3.22)
Zambia	207.9 (156.0, 263.2)	18.5 (-23.0, 67.0)	1.14 (0.86, 1.44)	-48.4 (-66.0, -27.0)	2.76 (1.97, 3.50)	-36.4 (-61.0, -9.00)	-2.50 (-3.30, -1.70)
Democratic People's Republic of Korea	2372.6 (1440.2, 3471.7)	32.9 (-16.0, 114.0)	9.04 (5.49, 13.23)	6.68 (-33.0, 72.0)	7.90 (4.81, 11.54)	-36.7 (-60.0, -2.00)	-1.80 (-2.21, -1.39)
Dominican Republic	142.1 (88.9, 199.8)	6.83 (-35.0, 51.0)	1.31 (0.82, 1.84)	-29.3 (-57.0, 0)	1.46 (0.93, 2.02)	-43.3 (-66.0, -20.0)	-2.01 (-2.87, -1.14)
Guatemala	60.2 (45.6, 77.7)	-1.63 (-31.0, 32.0)	0.34 (0.26, 0.44)	-55.9 (-69.0, -41.0)	0.52 (0.39, 0.67)	-64.6 (-75.0, -53.0)	-3.71 (-5.09, -2.31)

El Salvador	36.0 (26.1, 48.1)	-34.0 (-54.0, -4.00)	0.58 (0.42, 0.77)	-41.4 (-61.0, -19.0)	0.59 (0.43, 0.78)	-61.8 (-75.0, -47.0)	-4.62 (-6.51, -2.70)
Honduras	47.1 (25.5, 65.3)	98.7 (1.00, 180.0)	0.48 (0.26, 0.67)	-4.67 (-52.0, 34.0)	0.82 (0.44, 1.13)	-26.4 (-63.0, 4.00)	-3.19 (-6.53, 0.27)
Timor-Leste	29.1 (18.7, 42.2)	38.0 (-22.0, 102.0)	2.18 (1.40, 3.16)	-23.3 (-54.0, 18.0)	3.07 (2.05, 4.38)	-37.3 (-58.0, -6.00)	-1.85 (-3.74, 0.09)
Guyana	15.2 (10.9, 20.2)	-38.2 (-57.0, -14.0)	1.97 (1.41, 2.62)	-38.3 (-57.0, -14.0)	2.13 (1.55, 2.81)	-53.3 (-67.0, -36.0)	-2.45 (-4.83, -0.02)
Namibia	33.2 (24.0, 44.4)	-29.3 (-53.0, 11.0)	1.38 (1.00, 1.85)	-58.6 (-73.0, -35.0)	2.19 (1.60, 2.85)	-62.3 (-75.0, -43.0)	-3.86 (-5.51, -2.19)
Kyrgyzstan	174.4 (136.3, 216.2)	-45.1 (-58.0, -31.0)	2.67 (2.09, 3.31)	-62.5 (-71.0, -53.0)	3.05 (2.39, 3.78)	-66.5 (-74.0, -58.0)	-4.92 (-5.79, -4.04)
Tajikistan	235.0 (187.4, 295.4)	-23.2 (-40.0, -2.00)	2.48 (1.97, 3.11)	-56.5 (-66.0, -44.0)	3.56 (2.87, 4.43)	-59.4 (-68.0, -48.0)	-4.31 (-5.01, -3.61)
India	128557.1 (96112.1, 157021.2)	24.1 (-5.00, 58.0)	9.24 (6.91, 11.3)	-23.7 (-41.0, -3.00)	11.5 (8.63, 14.0)	-47.9 (-61.0, -32.0)	-2.69 (-2.83, -2.54)
Tuvalu	1.11 (0.74, 1.61)	-33.4 (-53.0, -4.00)	9.38 (6.29, 13.7)	-47.3 (-63.0, -24.0)	10.7 (7.23, 15.3)	-52.8 (-66.0, -34.0)	-2.43 (-10.75, 6.67)
Nicaragua	28.2 (22.3, 34.7)	-30 (-49.0, -09.0)	0.43 (0.34, 0.53)	-58.2 (-69.0, -46.0)	0.63 (0.50, 0.76)	-64.8 (-75.0, -55.0)	-5.66 (-7.32, -3.97)
Viet Nam	1510.9 (1021.8, 1938.3)	-23.2 (-58.0, 21.0)	1.57 (1.06, 2.01)	-45.8 (-70.0, -15.0)	1.80 (1.22, 2.29)	-63.9 (-81.0, -43.0)	-4.14 (-4.47, -3.81)
Kenya	412.5 (248.5, 599.1)	53.3 (15.0, 104.0)	0.82 (0.49, 1.19)	-29.2 (-47.0, -6.00)	1.84 (0.98, 2.82)	-32.4 (-51.0, -14.0)	-1.50 (-2.13, -0.87)
Kiribati	16.3 (11.9, 21.4)	6.90 (-28.0, 51.0)	13.7 (10.0, 18.0)	-33.3 (-55.0, -6.00)	18.8 (13.9, 24.2)	-38.0 (-58.0, -15.0)	-1.77 (-4.10, 0.61)
Nigeria	1984.7 (1403.8, 3067.7)	-38.4 (-59.0, -11.0)	0.92 (0.65, 1.43)	-74.1 (-83.0, -63.0)	2.04 (1.48, 3.09)	-69.5 (-79.0, -56.0)	-4.59 (-4.82, -4.36)
Lesotho	61.6 (41.9, 87.1)	-14.0 (-42.0, 38.0)	2.94 (2.00, 4.16)	-22.6 (-50.0, 19.0)	4.38 (3.05, 6.09)	-29.6 (-54.0, 5.00)	-0.24 (-1.57, 1.12)
Palestine	16.3 (13.1, 19.5)	-23.0 (-51.0, 20.0)	0.33 (0.26, 0.39)	-66.7 (-80.0, -50.0)	0.74 (0.58, 0.89)	-65.7 (-80.0, -50.0)	-4.95 (-7.47, -2.35)

Morocco	650.6 (482.1, 863.8)	-18.0 (-41.0, 14.0)	1.81 (1.34, 2.40)	-42.3 (-59.0, -20.0)	2.19 (1.64, 2.87)	-56.6 (-70.0, -39.0)	-3.60 (-4.00, -3.21)
Mauritania	33.9 (24.2, 46.1)	-44.8 (-62.0, -17.0)	0.84 (0.60, 1.15)	-71.6 (-80.0, -57.0)	1.65 (1.20, 2.19)	-71.8 (-80.0, -58.0)	-4.34 (-6.16, -2.47)
Marshall Islands	6.32 (4.15, 9.32)	24.1 (-15.0, 71.0)	11.1 (7.30, 16.4)	-21.0 (-32.0, 38.0)	14.6 (9.31, 21.1)	-34.5 (-55.0, -12.0)	-1.18 (-5.04, 2.83)
Venezuela (Bolivarian Republic of)	175.7 (127.1, 238.3)	-25.5 (-47.0, 3.00)	0.63 (0.45, 0.85)	-50.0 (-65.0, -31.0)	0.61 (0.44, 0.82)	-67.6 (-77.0, -55.0)	-5.32 (-6.11, -4.52)
Mongolia	122.5 (92.3, 159.4)	-13.9 (-42.0, 32.0)	3.62 (2.72, 4.71)	-45.2 (-63.0, -16.0)	5.42 (4.25, 6.78)	-59.8 (-74.0, -38.0)	-4.25 (-5.54, -2.95)
Eswatini	20.1 (13.7, 28.2)	-11.4 (-43.0, 39.0)	1.76 (1.20, 2.47)	-37.4 (-60.0, -2.00)	2.91 (2.06, 3.99)	-56.0 (-68.0, -21.0)	-1.91 (-3.96, 0.18)
Micronesia (Federated States of)	10.7 (6.22, 16.4)	-24.2 (-56.0, 17.0)	10.5 (6.09, 16.0)	-22.6 (-55.0, 19.0)	13.7 (8.49, 20.5)	-43.3 (-65.0, -15.0)	-1.96 (-4.70, 0.86)
Lao People's Democratic Republic	204.0 (140.4, 274.5)	-5.05 (-39.0, 39.0)	2.85 (1.96, 3.83)	-44.9 (-64.0, -19.0)	3.58 (2.51, 4.72)	-53.7 (-69.0, -33.0)	-2.69 (-3.32, -2.07)
Sao Tome and Principe	4.66 (2.88, 6.63)	-9.61 (-42.0, 30.0)	2.27 (1.40, 3.23)	-46.5 (-66.0, -23.0)	4.49 (2.72, 6.34)	-41.6 (-63.0, -17.0)	-2.79 (-8.13, 2.86)
Bolivia (Plurinational State of)	227.9 (161.6, 308.9)	-9.73 (-35.0, 26.0)	1.90 (1.35, 2.57)	-51.8 (-65.0, -33.0)	2.75 (1.97, 3.65)	-63.1 (-74.0, -49.0)	-4.53 (-5.30, -3.76)
Cameroon	291.3 (192.1, 433.7)	4.62 (-34.0, 57.0)	1.00 (0.66, 1.49)	-62.7 (-76.0, -44.0)	2.32 (1.62, 3.33)	-61.9 (-75.0, -44.0)	-3.10 (-3.75, -2.44)
Congo	73.7 (51.4, 103.2)	-14.0 (-40.0, 31.0)	1.40 (0.98, 1.96)	-58.4 (-72.0, -39.0)	2.82 (2.02, 3.78)	-59.5 (-72.0, -43.0)	-4.04 (-5.24, -2.82)
Syrian Arab Republic	200.5 (138.1, 276.2)	-74.4 (-85.0, -6.00)	1.38 (0.95, 1.91)	-77.2 (-87.0, -64.0)	1.66 (1.17, 2.25)	-83.6 (-91.0, -73.0)	-9.21 (-9.75, -8.67)
Belize	3.70 (2.88, 4.62)	56.3 (15.0, 110.0)	0.90 (0.70, 1.13)	-29.2 (-48.0, -5.00)	1.07 (0.84, 1.34)	-45.3 (-59.0, -28.0)	-2.90 (-8.49, 3.04)
Cabo Verde	5.60 (4.16, 6.79)	-66.1 (-74.0, -58.0)	0.99 (0.74, 1.20)	-78.8 (-84.0, -74.0)	1.30 (0.95, 1.57)	-87.0 (-85.0, -76.0)	-6.63 (-11.2, -1.83)

Nauru	0.76 (0.48, 1.04)	-16.8 (-45.0, 22.0)	7.21 (4.51, 9.82)	-19.2 (-46.0, 18.0)	10.6 (7.23, 14.0)	-28.2 (-51.0, 4.00)	-0.79 (-10.7, 10.2)
Low SDI							
Vanuatu	33.5 (21.3, 51.1)	98.7 (33.0, 201.0)	11.4 (7.24, 17.4)	2.12 (-32.0, 55.0)	16.0 (10.1, 24.0)	-19.0 (-44.0, 19.0)	-0.78 (-2.66, 1.13)
Yemen	634.9 (402.5, 973.8)	26.0 (-25.0, 90.0)	2.02 (1.28, 3.09)	-47.5 (-67.0, -17.0)	4.04 (2.67, 6.09)	-47.8 (-68.0, -18.0)	-2.89 (-3.33, -2.44)
Uganda	344.7 (251.1, 453.5)	14.1 (-25.0, 73.0)	0.84 (0.61, 1.10)	-52.0 (-68.0, -27.0)	1.98 (1.49, 2.54)	-47.4 (-64.0, -25.0)	-3.07 (-3.70, -2.44)
Gambia	26.0 (18.2, 34.4)	29.0 (-16.0, 94.0)	1.16 (0.81, 1.53)	-43.0 (-63.0, -14.0)	2.58 (1.83, 3.38)	-48.9 (-65.0, -27.0)	-2.37 (-4.74, 0.07)
Afghanistan	1013.9 (647.3, 1495.8)	25.0 (-16.0, 79.0)	2.65 (1.69, 3.91)	-64.1 (-75.0, -47.0)	6.15 (3.61, 9.65)	-45.0 (-59.0, -21.0)	-2.81 (-3.12, -2.50)
Djibouti	10.7 (6.58, 15.6)	48.4 (-11.0, 147.0)	0.89 (0.55, 1.30)	-40.0 (-64.0, 0)	1.81 (1.18, 2.54)	-49.9 (-68.0, -22.0)	-3.07 (-6.50, 0.47)
Guinea	226.0 (165.6, 302.3)	-28.0 (-44.0, 6.00)	1.79 (1.31, 2.39)	-61.2 (-73.0, -48.0)	3.61 (2.70, 4.83)	-53.8 (-67.0, -39.0)	-2.09 (-2.85, -1.33)
Burundi	172.6 (117.6, 239.5)	-13.0 (-41.0, 25.0)	1.45 (0.99, 2.01)	-59.4 (-73.0, -42.0)	3.05 (2.09, 4.22)	-58.0 (-67.0, -30.0)	-3.48 (-4.30, -2.65)
Bangladesh	3405.2 (2452.8, 4381.1)	14.0 (-20.0, 58.0)	2.14 (1.54, 2.75)	-22.0 (-45.0, 8.00)	2.74 (1.93, 3.57)	-43.5 (-61.0, -24.0)	-2.61 (-2.82, -2.39)
Bhutan	68.7 (33.4, 138.6)	-1.57 (-48.0, 63.0)	9.11 (4.42, 18.38)	-21.0 (-58.0, 32.0)	12.5 (6.04, 25.0)	-49.7 (-76.0, -20.0)	-3.42 (-4.77, -2.06)
Eritrea	103.9 (75.2, 143.1)	17.5 (-23.0, 75.0)	1.55 (1.12, 2.13)	-47.5 (-66.0, -22.0)	3.53 (2.60, 4.71)	-45.0 (-67.0, -16.0)	-2.68 (-3.72, -1.64)
Cambodia	370.8 (234.5, 520.9)	-27.3 (-47.0, -2.00)	2.23 (1.41, 3.14)	-54.6 (-67.0, -39.0)	2.71 (1.76, 3.73)	-64.7 (-75.0, -52.0)	-4.02 (-4.45, -3.58)
Comoros	10.3 (7.15, 14.2)	-7.41 (-43.0, 108.0)	1.44 (1.00, 1.99)	-39.6 (-63.0, 36.0)	2.12 (1.49, 2.94)	-49.6 (-68.0, -6.00)	-3.70 (-7.16, -0.12)
Guinea-Bissau	35.9 (25.3, 48.5)	-31.7 (-55.0, 6.00)	1.89 (1.33, 2.55)	-63.8 (-76.0, -44.0)	4.37 (3.15, 5.81)	-63.0 (-73.0, -40.0)	-2.96 (-4.55, -1.34)

Liberia	52.5 (33.0, 83.5)	-26.8 (-54.0, 14.0)	1.10 (0.69, 1.74)	-70.0 (-81.0, -53.0)	2.47 (1.56, 3.94)	-59.3 (-74.0, -36.0)	-3.56 (-5.03, -2.06)
Madagascar	583.2 (406.0, 810.7)	11.1 (-25.0, 57.0)	2.18 (1.52, 3.04)	-53.0 (-66.0, -30.0)	4.22 (2.93, 5.65)	-38.3 (-58.0, -15.0)	-2.16 (-2.61, -1.71)
Mali	350.2 (241.9, 497.7)	-14.4 (-37.0, 14.0)	1.60 (1.10, 2.27)	-66.1 (-75.0, -55.0)	3.56 (2.53, 5.12)	-64.0 (-69.0, -50.0)	-3.47 (-4.07, -2.87)
Haiti	734.4 (425.3, 1188.3)	-5.63 (-37.0, 32.0)	5.92 (3.43, 9.58)	-51.6 (-68.0, -32.0)	7.89 (4.53, 12.8)	-54.2 (-69.0, -35.0)	-2.63 (-2.97, -2.29)
Pakistan	21136.7 (15639.5, 27308.1)	39.1 (-2.00, 86.0)	9.43 (6.98, 12.2)	-30.0 (-51.0, -6.00)	16.9 (12.8, 21.4)	-35.0 (-50.0, -8.00)	-1.49 (-1.64, -1.34)
Ethiopia	1122.9 (816.5, 1470.0)	-37.9 (-55.0, -9.00)	1.04 (0.76, 1.37)	-73.0 (-79.0, -56.0)	2.34 (1.61, 2.97)	-64.7 (-75.0, -48.0)	-5.14 (-5.43, -4.85)
Zimbabwe	575.0 (385.4, 780.1)	62.3 (4.00, 135.0)	3.83 (2.57, 5.20)	11.8 (-28.0, 62.0)	5.71 (3.79, 7.76)	-1.91 (-37.0, 37.0)	1.38 (0.86, 1.89)
Mozambique	315.6 (230.0, 405.8)	20.0 (-22.0, 71.0)	1.07 (0.78, 1.37)	-46.9 (-65.0, -24.0)	2.63 (1.92, 3.41)	-33.9 (-59.0, -1.00)	-1.42 (-2.11, -0.72)
Angola	397.2 (275.1, 534.4)	14.7 (-20.0, 66.0)	1.32 (0.91, 1.77)	-67.0 (-73.0, -43.0)	3.24 (2.23, 4.26)	-53.9 (-68.0, -32.0)	-3.47 (-3.99, -2.96)
South Sudan	103.4 (58.3, 170.2)	-17.3 (-44.0, 31.0)	1.11 (0.63, 1.83)	-47.8 (-64.0, -18.0)	2.24 (1.30, 3.53)	-43.3 (-60.0, -16.0)	-2.28 (-3.34, -1.20)
Nepal	2808.1 (1802.9, 4214.6)	12.0 (-27.0, 64.0)	9.23 (5.93, 13.9)	-28.1 (-53.0, 5.00)	13.4 (8.76, 20.5)	-45.3 (-67.0, -17.0)	-3.10 (-3.34, -2.87)
Burkina Faso	305.1 (227.1, 396.6)	53.8 (11.0, 108.0)	1.34 (1.00, 1.75)	-35.2 (-53.0, -12.0)	2.90 (2.25, 3.60)	-27.4 (-46.0, -2.00)	-1.28 (-2.13, -0.43)
Niger	370.2 (239.2, 593.1)	16.3 (-19.0, 71.0)	1.59 (1.03, 2.55)	-60.0 (-72.0, -41.0)	3.92 (2.62, 6.48)	-54.3 (-66.0, -36.0)	-2.79 (-3.43, -2.16)

Democratic Republic of the Congo	1570.1 (917.2, 2819.0)	29.3 (-18.0, 109.0)	1.79 (1.05, 3.22)	-43.1 (-64.0, -8.00)	4.16 (2.39, 7.39)	-37.4 (-60.0, -1.00)	-2.14 (-2.42, -1.85)
Malawi	184.4 (134.2, 236.4)	-13.7 (-40.0, 37.0)	1.00 (0.73, 1.28)	-55.3 (-69.0, -29.0)	2.12 (1.57, 2.68)	-47.8 (-62.0, -29.0)	-3.14 (-3.97, -2.31)
Papua New Guinea	1183.6 (686.9, 1881.7)	111.4 (58.0, 193.0)	12.0 (6.96, 19.1)	-12.4 (-34.0, 21.0)	18.2 (10.4, 30.5)	-19.1 (-38.0, 10.0)	-0.44 (-0.77, -0.10)
Central African Republic	182.2 (100.8, 294.6)	31.0 (-12.0, 87.0)	3.44 (1.90, 5.56)	-32.6 (-54.0, -3.00)	7.47 (4.14, 12.3)	-27.4 (-49.0, 3.00)	-1.49 (-2.31, -0.66)
Benin	158.4 (116.9, 212.0)	-57.0 (-29.0, 35.0)	1.25 (0.92, 1.67)	-61.9 (-73.0, -48.0)	2.81 (2.16, 3.67)	-58.2 (-69.0, -45.0)	-2.84 (-3.76, -1.91)
Rwanda	143.9 (96.2, 194.9)	-47.5 (-63.0, -25.0)	1.13 (0.76, 1.54)	-73.0 (-79.0, -57.0)	2.28 (1.50, 3.12)	-68.0 (-79.0, -53.0)	-5.94 (-6.70, -5.17)
Senegal	175.3 (121.8, 237.3)	-8.65 (-36.0, 27.0)	1.16 (0.80, 1.57)	-54.0 (-68.0, -36.0)	2.27 (1.64, 3.00)	-56.2 (-67.0, -42.0)	-2.72 (-3.59, -1.83)
Solomon Islands	90.8 (43.5, 140.1)	55.5 (02.0, 121.0)	13.9 (6.63, 21.4)	-19.3 (-47.0, 15.0)	21.1 (10.9, 31.2)	-31.8 (-54.0, -5.00)	-1.12 (-2.24, 0.01)
Togo	98.7 (65.7, 144.9)	13.3 (-24.0, 58.0)	1.25 (0.83, 1.83)	-47.6 (-65.0, -27.0)	2.64 (1.85, 3.73)	-55.4 (-68.0, -39.0)	-2.51 (-3.61, -1.39)
Sierra Leone	118.3 (75.6, 168.9)	-17.0 (-39.0, 28.0)	1.43 (0.91, 2.04)	-66.0 (-73.0, -43.0)	2.86 (1.88, 3.95)	-52.6 (-67.0, -34.0)	-1.93 (-3.03, -0.81)
United Republic of Tanzania	535.0 (408.6, 712.0)	6.47 (-25.0, 53.0)	0.94 (0.72, 1.26)	-51.4 (-66.0, -30.0)	1.91 (1.44, 2.41)	-48.6 (-62.0, -31.0)	-2.84 (-3.37, -2.30)
Somalia	432.7 (248.5, 767.6)	72.9 (14.0, 162.0)	2.13 (1.22, 3.77)	-39.3 (-60.0, -8.00)	4.95 (2.79, 8.83)	-33.9 (-55.0, -5.00)	-1.94 (-2.52, -1.35)
Chad	263.0 (197.3, 371.2)	11.8 (-16.0, 52.0)	1.60 (1.20, 2.26)	-59.0 (-69.0, -44.0)	3.93 (2.94, 5.60)	-47.1 (-60.0, -31.0)	-1.87 (-2.65, -1.09)

Abbreviation: RHD, rheumatic heart disease; SDI, Socio-demographic Index.

Supplementary Table 3. The temporal change of DALYs from RHD by SDI quintiles in 204 countries and territories during 1990-2019.

	DALY		All-age DALY		Age-standardized DALY		
	Number in 2019, n×1,000	Percent change 1990-2019, 100%	Rate in 2019, Rate per 100,000	Percent change 1990-2019, %	Rate in 2019, Rate per 100,000	Percent change 1990-2019, %	Net drift, % per year
High SDI							
Denmark	1309.2 (1065.8, 1545.0)	-71.8 (-77.0, -66.0)	22.6 (18.4, 26.6)	-75.0 (-80.0, -70.0)	11.6 (9.56, 13.6)	-81.0 (-84.0, -76.0)	-4.51 (-5.40, -3.62)
Puerto Rico	761.0 (582.2, 985.9)	-43.6 (-58.0, -26.0)	21.6 (16.5, 28.0)	-42.1 (-56.0, -24.0)	14.2 (10.7, 18.6)	-61.9 (-72.0, -50.0)	-3.69 (-4.07, -3.31)
Switzerland	2046.6 (1713.7, 2377.8)	-64.0 (-67.0, -53.0)	23.3 (19.5, 27.1)	-69.0 (-74.0, -63.0)	11.3 (9.59, 13.1)	-77.1 (-81.0, -73.0)	-5.37 (-5.87, -4.86)
Russian Federation	88897.6 (75877.7, 102250.0	-74.8 (-78.0, -71.0)	60.6 (51.7, 69.7)	-74.1 (-78.0, -70.0)	40.8 (34.8, 47.2)	-79.3 (-82.0, -76.0)	-6.76 (-7.13, -6.39)
Germany	60419.7 (51888.6, 70457.9)	-29.3 (-40.0, -16.0)	71.2 (61.1, 83.0)	-33.5 (-44.0, -21.0)	30.1 (26.1, 34.8)	-56.8 (-63.0, -49.0)	-3.57 (-3.93, -3.21)
Finland	903.5 (752.7, 1083.1)	-67.0 (-73.0, -6.00)	16.3 (13.6, 19.6)	-71.0 (-75.0, -64.0)	7.76 (6.58, 9.30)	-80.0 (-83.0, -76.0)	-4.78 (-5.48, -4.07)
France	33977.3 (28097.8, 39856.4)	-17.0 (-26.0, 5.00)	51.3 (42.4, 60.2)	-22.1 (-35.0, -8.00)	24.3 (20.5, 28.1)	-48.8 (-56.0, -40.0)	-2.54 (-2.74, -2.34)
Czechia	5606.5 (4535.2, 6841.3)	-74.0 (-76.0, -63.0)	52.7 (42.6, 64.3)	-71.4 (-77.0, -64.0)	28.3 (22.7, 34.4)	-84.0 (-84.0, -76.0)	-5.89 (-6.23, -5.56)
United Kingdom	21121.4 (19446.5, 22693.2)	-67.0 (-63.0, -58.0)	31.4 (28.9, 33.8)	-66.4 (-68.0, -64.0)	17.6 (16.3, 19.0)	-71.6 (-73.0, -70.0)	-4.30 (-4.53, -4.06)
San Marino	24.8 (16.8, 35.2)	39.4 (-10.0, 113.0)	74.8 (50.8, 106.2)	-81.0 (-36.0, 52.0)	38.7 (26.0, 56.0)	-29.8 (-55.0, 10.0)	-0.92 (-6.28, 4.75)

Brunei Darussalam	199.3 (169.1, 233.0)	18.9 (-6.00, 56.0)	45.6 (38.69, 53.3)	-29.7 (-45.0, -7.00)	58.3 (50.2, 67.0)	-48.9 (-59.0, -34.0)	-2.66 (-3.45, -1.87)
Kuwait	437.2 (339.9, 557.7)	-37.5 (-52.0, -18.0)	9.88 (7.68, 12.6)	-75.2 (-81.0, -67.0)	12.0 (9.64, 15.0)	-78.6 (-83.0, -73.0)	-4.67 (-5.13, -4.21)
Ireland	1172.7 (968.2, 1406.9)	-46.0 (-52.0, -27.0)	23.9 (19.7, 28.7)	-56.5 (-65.0, -46.0)	16.2 (13.4, 19.5)	-67.5 (-74.0, -60.0)	-3.57 (-4.12, -3.01)
Republic of Korea	6653.2 (5668.0, 7715.2)	-36.1 (-46.0, -24.0)	12.5 (10.6, 14.5)	-46.9 (-55.0, -37.0)	8.20 (6.99, 9.50)	-72.1 (-76.0, -67.0)	-5.30 (-5.51, -5.08)
Taiwan (Province of China)	7355.6 (5637.1, 9377.5)	-58.2 (-68.0, -47.0)	31.1 (23.9, 39.7)	-63.9 (-72.0, -54.0)	20.0 (15.4, 25.3)	-81.3 (-85.0, -77.0)	-6.35 (-6.51, -6.19)
Luxembourg	202.7 (166.9, 240.3)	-13.2 (-31.0, 6.00)	32.8 (27.0, 38.8)	-46.5 (-58.0, -34.0)	20.6 (17.0, 24.5)	-53.9 (-63.0, -43.0)	-3.22 (-4.66, -1.76)
Singapore	752.7 (620.4, 916.9)	-62.9 (-71.0, -53.0)	13.3 (11.0, 16.2)	-80.0 (-84.0, -75.0)	10.1 (8.46, 12.2)	-85.9 (-89.0, -82.0)	-7.06 (-7.41, -6.71)
Slovenia	1614.9 (1222.7, 2138.7)	-31.8 (-53.0, -4.00)	77.9 (59.0, 103.1)	-35.2 (-56.0, -9.00)	37.6 (28.8, 49.4)	-61.8 (-74.0, -46.0)	-3.91 (-4.60, -3.23)
United States of America	125765.5 (113044.6, 140066.0)	-29.8 (-34.0, -25.0)	38.4 (34.5, 42.7)	-45.7 (-49.0, -42.0)	23.8 (21.5, 26.5)	-59.3 (-62.0, -57.0)	-3.59 (-3.77, -3.40)
Australia	8360.0 (7193.5, 9659.5)	-15.0 (-27.0, -1.00)	34.0 (29.3, 39.3)	-41.6 (-50.0, -32.0)	22.0 (19.0, 25.3)	-57.4 (-63.0, -50.0)	-2.86 (-3.08, -2.65)
Iceland	52.3 (43.5, 61.5)	-14.3 (-31.0, 4.00)	15.2 (12.6, 17.8)	-36.9 (-49.0, -24.0)	9.90 (8.28, 11.7)	-54.5 (-63.0, -45.0)	-2.57 (-4.78, -0.31)
Canada	15411.1 (12948.6, 18242.8)	92.0 (-14.0, 18.0)	42.2 (35.5, 50.0)	-24.7 (-36.0, -12.0)	23.5 (19.8, 27.7)	-58.0 (-58.0, -43.0)	-2.42 (-2.61, -2.24)
Japan	54816.8 (44933.9, 61647.4)	-9.70 (-23.0, -1.00)	42.9 (35.2, 48.2)	-11.1 (-24.0, -2.00)	14.1 (12.3, 15.6)	-62.5 (-66.0, -60.0)	-3.85 (-4.07, -3.64)

Slovakia	2562.7 (2024.2, 3155.3)	-31.9 (-47.0, -13.0)	47.1 (37.2, 58.0)	-33.8 (-48.0, -16.0)	29.4 (23.3, 36.4)	-54.1 (-64.0, -41.0)	-3.17 (-3.52, -2.81)
New Zealand	3114.6 (2708.2, 3524.1)	-23.4 (-34.0, -11.0)	69.3 (60.2, 78.4)	-41.7 (-50.0, -33.0)	48.6 (42.5, 55.1)	-55.7 (-62.0, -49.0)	-3.34 (-3.57, -3.11)
Estonia	711.1 (543.1, 950.7)	-77.7 (-84.0, -70.0)	54.2 (41.4, 72.4)	-73.3 (-8.0, -64.0)	33.3 (25.3, 44.3)	-79.5 (-85.0, -72.0)	-6.66 (-7.39, -5.93)
Belgium	6096.2 (5041.5, 7157.7)	88.0 (54.0, 124.0)	53.4 (44.2, 62.7)	64.3 (35.0, 96.0)	24.4 (20.6, 28.4)	11.7 (-08.0, 33.0)	-0.49 (-0.91, -0.07)
Qatar	293.2 (214.5, 390.4)	42.7 (-19.0, 148.0)	10.2 (7.49, 13.6)	-77.8 (-87.0, -61.0)	18.1 (14.1, 23.3)	-76.2 (-87.0, -62.0)	-6.14 (-6.78, -5.49)
Saudi Arabia	10293.8 (6972.9, 14351.3)	-11.5 (-59.0, 60.0)	28.8 (19.5, 40.2)	-62.0 (-82.0, -28.0)	29.8 (21.3, 39.2)	-79.0 (-86.0, -49.0)	-4.36 (-4.53, -4.20)
Latvia	1261.8 (992.1, 1659.8)	-84.0 (-88.0, -79.0)	65.9 (51.8, 86.7)	-77.8 (-83.0, -71.0)	41.1 (31.7, 54.1)	-82.7 (-87.0, -77.0)	-6.78 (-7.31, -6.24)
Austria	4340.6 (3758.8, 4963.8)	-37.4 (-46.0, -28.0)	48.7 (42.2, 55.7)	-45.4 (-53.0, -37.0)	23.4 (20.5, 26.5)	-61.2 (-66.0, -55.0)	-3.80 (-4.24, -3.35)
Andorra	25.7 (19.4, 33.4)	46.3 (-4.00, 118.0)	31.0 (23.4, 40.2)	-4.70 (-38.0, 42.0)	18.6 (14.1, 24.2)	-44.5 (-63.0, -17.0)	-2.19 (-6.45, 2.27)
Netherlands	4347.9 (3668.4, 5106.0)	6.14 (-11.0, 28.0)	25.3 (21.4, 29.8)	-7.68 (-23.0, 11.0)	13.0 (11.1, 15.2)	-38.7 (-48.0, -27.0)	-2.48 (-3.02, -1.94)
United Arab Emirates	8338.7 (5702.2, 11948.5)	254.8 (97.0, 539.0)	90.2 (61.7, 129.3)	-28.1 (-60.0, 29.0)	108.7 (71.9, 156.1)	-58.1 (-78.0, -21.0)	-2.60 (-2.77, -2.42)
Lithuania	2121.1 (1662.6, 2669.1)	-83.0 (-85.0, -75.0)	75.9 (59.5, 95.5)	-74.1 (-80.0, -67.0)	46.2 (35.8, 58.1)	-81.4 (-86.0, -76.0)	-6.39 (-6.86, -5.91)
Bermuda	14.7 (11.8, 18.7)	-64.0 (-7.00, -49.0)	23.0 (18.4, 29.2)	-63.3 (-72.0, -52.0)	15.9 (12.7, 20.2)	-72.4 (-79.0, -64.0)	-4.80 (-7.27, -2.27)

Cyprus	769.3 (656.3, 917.9)	-28.0 (-44.0, 2.00)	58.6 (50.0, 70.0)	-53.1 (-67.0, -4.00)	41.4 (35.4, 48.9)	-67.0 (-76.0, -58.0)	-4.40 (-5.36, -3.43)
Guam	216.0 (178.7, 259.7)	-6.25 (-25.0, 15.0)	126.6 (104.8, 152.2)	-24.9 (-40.0, -8.00)	125.9 (104.0, 151.3)	-34.2 (-46.0, -2.00)	-1.74 (-2.39, -1.09)
Norway	1194.2 (1058.3, 1323.0)	-48.2 (-52.0, -44.0)	22.3 (19.8, 24.7)	-58.9 (-62.0, -55.0)	12.0 (10.8, 13.3)	-63.9 (-67.0, -61.0)	-4.47 (-5.15, -3.78)
Sweden	2621.3 (2185.8, 3076.3)	-47.1 (-55.0, -38.0)	25.6 (21.4, 30.1)	-55.5 (-62.0, -48.0)	12.0 (10.2, 14.0)	-62.8 (-68.0, -57.0)	-3.43 (-3.90, -2.97)
Monaco	10.3 (8.15, 12.3)	-14.6 (-34.0, 9.00)	27.3 (21.7, 32.7)	-38.0 (-46.0, -11.0)	11.7 (9.23, 14.2)	-37.2 (-52.0, -18.0)	-1.40 (-9.11, 6.97)
High-middle SDI							
Dominica	76.8 (59.4, 96.5)	-33.7 (-46.0, -19.0)	111.8 (86.5, 140.5)	-28.6 (-42.0, -13.0)	103.4 (79.1, 131.1)	-39.4 (-51.0, -26.0)	-1.87 (-2.90, -0.82)
Croatia	1951.2 (1546.5, 2466.0)	-68.2 (-75.0, -58.0)	45.9 (36.4, 58.1)	-63.4 (-72.0, -52.0)	23.4 (18.5, 29.7)	-75.8 (-82.0, -69.0)	-5.04 (-5.51, -4.58)
Ukraine	34901.3 (27573.2, 43527.7)	-34.7 (-54.0, -8.00)	79.2 (62.6, 98.8)	-21.9 (-45.0, 10.0)	54.5 (43.1, 68.1)	-29.6 (-51.0, 0)	-1.34 (-1.87, -0.80)
Hungary	5365.1 (4375.2, 6482.1)	-77.4 (-82.0, -72.0)	55.5 (45.2, 67.0)	-75.7 (-81.0, -70.0)	29.7 (24.1, 36.0)	-82.6 (-86.0, -79.0)	-6.66 (-6.96, -6.35)
Argentina	39817.5 (32337.7, 48732.1)	-24.0 (-35.0, -12.0)	88.3 (71.7, 108.0)	-44.2 (-52.0, -35.0)	78.7 (63.1, 98.1)	-52.1 (-59.0, -44.0)	-2.55 (-2.67, -2.43)
Saint Kitts and Nevis	22.6 (13.9, 31.6)	-56.3 (-74.0, -35.0)	38.0 (23.4, 53.1)	-69.7 (-82.0, -55.0)	33.4 (21.0, 46.1)	-76.7 (-86.0, -66.0)	-4.75 (-6.48, -3.00)
North Macedonia	1017.0 (786.0, 1285.0)	-57.6 (-70.0, -43.0)	47.2 (36.5, 59.7)	-63.0 (-72.0, -47.0)	33.8 (26.4, 42.5)	-71.8 (-80.0, -62.0)	-5.71 (-6.08, -5.34)
Kazakhstan	13166.4 (10030.2, 16906.1)	-69.7 (-77.0, -60.0)	71.6 (54.5, 91.9)	-73.1 (-80.0, -65.0)	68.1 (52.3, 86.6)	-76.5 (-82.0, -69.0)	-6.81 (-7.16, -6.47)

Romania	10541.8 (8464.7, 12942.1)	-85.0 (-84.0, -76.0)	54.8 (44.0, 67.3)	-76.2 (-81.0, -71.0)	33.4 (26.6, 41.0)	-83.4 (-87.0, -80.0)	-7.18 (-7.56, -6.80)
Chile	5270.9 (4394.9, 6333.3)	-64.4 (-71.0, -57.0)	29.0 (24.2, 34.8)	-74.0 (-79.0, -68.0)	23.4 (19.7, 28.0)	-82.1 (-85.0, -78.0)	-6.31 (-6.45, -6.16)
Poland	22278.0 (18922.8, 25934.6)	-80.0 (-83.0, -77.0)	58.0 (49.2, 67.5)	-81.0 (-83.0, -77.0)	34.5 (29.2, 40.1)	-86.7 (-89.0, -85.0)	-7.72 (-7.98, -7.46)
Montenegro	314.1 (249.6, 385.7)	-14.5 (-41.0, 17.0)	50.6 (40.2, 62.2)	-13.7 (-40.0, 18.0)	35.0 (27.8, 43.1)	-38.6 (-57.0, -17.0)	-2.74 (-3.67, -1.79)
Greece	2513.3 (2109.2, 2971.2)	-38.2 (-48.0, -26.0)	24.3 (20.4, 28.7)	-37.8 (-48.0, -25.0)	11.1 (9.26, 13.1)	-69.0 (-67.0, -53.0)	-3.47 (-3.88, -3.05)
Malaysia	16872.4 (12585.5, 21737.2)	-32.4 (-47.0, -16.0)	53.9 (40.2, 69.4)	-61.9 (-70.0, -53.0)	52.7 (39.6, 67.4)	-70.0 (-76.0, -61.0)	-3.53 (-3.61, -3.45)
United States Virgin Islands	37.5 (29.8, 46.8)	-41.1 (-56.0, -20.0)	36.1 (28.7, 45.0)	-40.0 (-56.0, -19.0)	27.6 (21.5, 35.3)	-54.9 (-67.0, -38.0)	-3.23 (-4.90, -1.52)
Lebanon	1287.2 (660.9, 1886.7)	-41.8 (-74.0, -8.00)	24.9 (12.8, 36.4)	-63.2 (-84.0, -42.0)	24.6 (12.7, 36.2)	-69.6 (-87.0, -51.0)	-4.37 (-4.61, -4.13)
Serbia	3063.9 (2477.6, 3738.4)	-52.3 (-69.0, -33.0)	35.0 (28.3, 42.7)	-48.8 (-66.0, -28.0)	20.2 (16.4, 24.6)	-65.4 (-77.0, -52.0)	-5.44 (-5.85, -5.02)
Antigua and Barbuda	72.2 (54.5, 94.7)	2.78 (-13.0, 19.0)	81.6 (61.6, 107.0)	-29.5 (-40.0, -19.0)	73.7 (55.3, 97.8)	-39.3 (-49.0, -29.0)	-1.88 (-3.04, -0.70)
Israel	3689.5 (3161.0, 4312.3)	19.9 (1.00, 42.0)	39.6 (34.0, 46.3)	-36.1 (-46.0, -24.0)	33.3 (28.5, 38.9)	-48.4 (-57.0, -39.0)	-2.97 (-3.20, -2.73)
Bulgaria	6176.9 (4541.8, 8229.6)	-75.4 (-82.0, -67.0)	89.1 (65.5, 118.7)	-69.2 (-78.0, -58.0)	56.4 (41.0, 75.0)	-74.4 (-82.0, -66.0)	-5.67 (-5.91, -5.43)
Italy	42875.0 (38213.7, 46965.2)	-31.6 (-38.0, -26.0)	71.1 (63.4, 77.9)	-35.6 (-41.0, -31.0)	31.3 (28.4, 34.0)	-59.4 (-63.0, -56.0)	-4.08 (-4.22, -3.95)

Barbados	251.9 (191.8, 328.0)	-16.3 (-32.0, -1.00)	84.6 (64.4, 110.1)	-28.6 (-42.0, -15.0)	75.2 (56.1, 100.4)	-35.0 (-47.0, -23.0)	-1.56 (-2.17, -0.94)
Trinidad and Tobago	1102.9 (806.3, 1459.4)	-36.0 (-50.0, -21.0)	79.5 (58.1, 105.2)	-44.5 (-57.0, -32.0)	73.9 (54.0, 98.4)	-59.0 (-62.0, -39.0)	-2.88 (-3.15, -2.60)
Bahamas	311.1 (227.9, 415.2)	14.8 (-8.00, 41.0)	82.5 (60.5, 110.1)	-21.9 (-37.0, -4.00)	75.3 (55.5, 100.9)	-31.5 (-45.0, -16.0)	-1.53 (-2.17, -0.89)
Bosnia and Herzegovina	856.5 (679.6, 1069.1)	-67.3 (-75.0, -58.0)	26.0 (20.6, 32.4)	-55.0 (-65.0, -42.0)	15.6 (12.4, 19.4)	-74.3 (-80.0, -67.0)	-5.86 (-6.33, -5.39)
Malta	169.2 (139.1, 204.5)	-17.0 (-29.0, 11.0)	38.5 (31.7, 46.6)	-24.6 (-40.0, -7.00)	19.9 (16.5, 24.0)	-56.1 (-65.0, -46.0)	-2.12 (-3.59, -0.62)
Jordan	1108.0 (899.7, 1348.5)	6.98 (-26.0, 53.0)	9.52 (7.73, 11.6)	-65.3 (-76.0, -50.0)	12.7 (10.2, 15.4)	-72.8 (-82.0, -6.00)	-5.07 (-5.35, -4.78)
Belarus	8434.0 (6024.3, 11709.2)	-72.2 (-81.0, -61.0)	88.8 (63.4, 123.2)	-69.2 (-79.0, -57.0)	58.4 (42.1, 80.6)	-76.0 (-83.0, -67.0)	-5.85 (-6.30, -5.40)
Seychelles	55.6 (43.4, 70.6)	-52.2 (-61.0, -42.0)	54.4 (42.4, 69.1)	-65.8 (-72.0, -59.0)	50.8 (39.9, 65.2)	-71.5 (-77.0, -65.0)	-3.77 (-4.91, -2.61)
Portugal	5082.2 (4358.9, 5930.6)	-55.2 (-62.0, -46.0)	47.7 (40.9, 55.7)	-57.2 (-64.0, -49.0)	23.7 (20.4, 27.7)	-73.8 (-78.0, -69.0)	-5.99 (-6.23, -5.74)
Cook Islands	15.4 (11.7, 19.7)	-48.3 (-64.0, -26.0)	85.6 (65.1, 109.3)	-45.3 (-62.0, -21.0)	77.5 (56.1, 101.7)	-56.4 (-71.0, -37.0)	-2.67 (-4.81, -0.49)
Libya	4058.1 (2923.2, 5398.6)	-31.8 (-57.0, 21.0)	60.3 (43.4, 80.2)	-57.1 (-73.0, -24.0)	61.4 (45.2, 81.5)	-53.6 (-70.0, -28.0)	-2.06 (-2.25, -1.87)
American Samoa	119.2 (95.4, 147.8)	-1.70 (-26.0, 31.0)	214.8 (171.8, 266.3)	-14.2 (-35.0, 14.0)	223.6 (179.2, 276.6)	-29.2 (-47.0, -5.00)	-1.31 (-2.17, -0.43)
Turkey	13436.2 (10743.5, 16587.9)	-35.8 (-59.0, -9.00)	16.5 (13.2, 20.4)	-52.9 (-7.00, -33.0)	15.2 (12.2, 18.7)	-68.7 (-80.0, -56.0)	-4.36 (-4.61, -4.11)

Bahrain	293.2 (230.6, 367.3)	57.7 (10.0, 122.0)	20.3 (16.0, 25.5)	-44.5 (-61.0, -22.0)	25.7 (20.6, 31.9)	-58.7 (-70.0, -44.0)	-4.24 (-4.83, -3.64)
Spain	34810.7 (29019.0, 41168.3)	-38.5 (-48.0, -27.0)	75.6 (63.1, 89.5)	-48.2 (-56.0, -39.0)	35.4 (29.9, 41.6)	-67.7 (-73.0, -62.0)	-5.29 (-5.52, -5.06)
Oman	280.4 (222.1, 366.9)	-37.4 (-62.0, -4.00)	6.12 (4.84, 8.00)	-73.5 (-84.0, -59.0)	12.9 (10.8, 15.3)	-72.2 (-84.0, -54.0)	-4.96 (-5.47, -4.45)
Greenland	23.1 (18.9, 27.6)	-42.7 (-55.0, -27.0)	41.1 (33.6, 49.1)	-43.3 (-56.0, -28.0)	35.4 (29.4, 42.2)	-68.9 (-75.0, -61.0)	-4.97 (-7.87, -1.97)
Niue	3.29 (2.06, 4.64)	-53.8 (-70.0, -30.0)	196.9 (123.3, 277.6)	-35.7 (-58.0, -2.00)	185.0 (115.6, 266.5)	-44.0 (-64.0, -13.0)	-2.36 (-6.61, 2.07)
Northern Mariana Islands	58.1 (47.5, 72.0)	-25.2 (-43.0, 2.00)	136.8 (111.8, 169.4)	-20.0 (-40.0, 8.00)	124.3 (101.7, 155.7)	-31.9 (-48.0, -8.00)	-0.86 (-2.16, 0.46)
Palau	28.3 (21.5, 35.7)	-16.0 (-46.0, 24.0)	156.9 (119.2, 198.0)	-28.2 (-53.0, 6.00)	140.0 (106.7, 174.7)	-41.6 (-62.0, -13.0)	-1.64 (-3.37, 0.11)
Middle SDI							
Costa Rica	3820.2 (2791.8, 5129.2)	19.5 (-2.00, 42.0)	81.0 (59.2, 108.8)	-23.0 (-37.0, -9.00)	75.6 (55.0, 101.8)	-38.8 (-50.0, -26.0)	-1.9 (-2.06, -1.73)
Iran (Islamic Republic of)	51152.3 (42003.3, 62953.0)	-21.4 (-40.0, -4.00)	60.7 (49.8, 74.7)	-45.4 (-58.0, -33.0)	61.3 (51.3, 74.5)	-54.6 (-67.0, -45.0)	-2.73 (-2.91, -2.54)
Gabon	1579.3 (1107.8, 2208.1)	8.73 (-17.0, 42.0)	90.3 (63.3, 126.2)	-38.4 (-53.0, -19.0)	100.8 (73.2, 136.6)	-46.8 (-60.0, -29.0)	-2.29 (-2.54, -2.04)
Armenia	4012.1 (3194.4, 4919.2)	-53.4 (-62.0, -43.0)	132.9 (105.8, 162.9)	-47.3 (-57.0, -36.0)	111.0 (87.8, 137.1)	-58.9 (-67.0, -50.0)	-3.32 (-3.52, -3.12)
Azerbaijan	10312.8 (7817.5, 13252.2)	-13.7 (-30.0, 4.00)	100.3 (76.1, 128.9)	-38.4 (-50.0, -26.0)	95.5 (72.8, 121.3)	-47.1 (-57.0, -36.0)	-2.58 (-2.73, -2.43)

Grenada	134.5 (109.5, 166.1)	-32.7 (-45.0, -17.0)	130.3 (106.1, 160.9)	-44.2 (-54.0, -31.0)	120.8 (98.1, 149.2)	-55.9 (-64.0, -46.0)	-2.56 (-3.33, -1.78)
Iraq	22779.8 (17318.0, 29517.7)	-2.83 (-34.0, 38.0)	54.1 (41.1, 70.1)	-59.4 (-72.0, -42.0)	64.6 (51.0, 80.6)	-65.6 (-77.0, -51.0)	-4.33 (-4.49, -4.17)
Tokelau	3.24 (2.27, 4.47)	-55.1 (-70.0, -32.0)	229.8 (161.0, 317.1)	-46.3 (-64.0, -18.0)	242.3 (168.6, 334.9)	-53.6 (-69.0, -28.0)	-2.71 (-7.03, 1.80)
Sri Lanka	6244.4 (4553.3, 8260.4)	-56.1 (-68.0, -41.0)	28.6 (20.8, 37.8)	-65.4 (-75.0, -53.0)	26.1 (19.2, 34.3)	-73.2 (-80.0, -64.0)	-5.02 (-5.18, -4.86)
China	1705941.7 (1424796.0, 1984608.6)	-57.6 (-67.0, -47.0)	119.9 (100.2, 139.5)	-64.7 (-72.0, -56.0)	93.7 (78.4, 108.8)	-78.3 (-83.0, -73.0)	-5.55 (-5.76, -5.35)
Georgia	8652.1 (7053.9, 10279.2)	-18.2 (-34.0, 0)	236.1 (192.5, 280.5)	22.9 (0.0, 50.0)	182.3 (150.7, 215.4)	-53.0 (-18.0, 20.0)	0.46 (0.08, 0.84)
Botswana	2611.5 (1793.2, 3558.9)	12.7 (-18.0, 54.0)	111.7 (76.7, 152.2)	-37.3 (-54.0, -15.0)	118.4 (80.2, 159.7)	-49.6 (-64.0, -28.0)	-2.64 (-2.84, -2.44)
Thailand	30259.4 (22652.5, 39762.3)	-66.2 (-75.0, -55.0)	43.2 (32.3, 56.7)	-72.6 (-80.0, -64.0)	38.9 (28.8, 52.1)	-77.8 (-84.0, -71.0)	-6.63 (-6.93, -6.33)
Uruguay	1434.0 (1222.6, 1661.5)	-29.3 (-41.0, -15.0)	41.7 (35.6, 48.4)	-35.4 (-47.0, -22.0)	29.8 (25.1, 34.6)	-47.7 (-57.0, -37.0)	-2.33 (-2.68, -1.98)
South Africa	53596.1 (40791.1, 70455.2)	-13.7 (-28.0, 1.00)	96.4 (73.4, 126.7)	-42.8 (-53.0, -33.0)	95.2 (73.8, 123.7)	-47.1 (-56.0, -38.0)	-2.69 (-3.06, -2.33)
Saint Lucia	198.6 (154.8, 254.7)	-17.4 (-33.0, 1.00)	113.8 (88.6, 145.8)	-35.0 (-47.0, -21.0)	100.5 (77.9, 128.9)	-53.7 (-63.0, -43.0)	-2.72 (-3.41, -2.04)
Mexico	51145.1 (41392.4, 64409.4)	-39.9 (-49.0, -30.0)	40.9 (33.1, 51.6)	-58.9 (-65.0, -52.0)	40.6 (33.1, 51.1)	-70.0 (-75.0, -65.0)	-4.34 (-4.44, -4.24)

Fiji	2970.6 (2329.7, 3798.2)	-17.6 (-42.0, 20.0)	326.0 (255.7, 416.8)	-31.3 (-51.0, 0)	334.7 (263.5, 425.7)	-39.4 (-57.0, -12.0)	-1.69 (-1.89, -1.50)
Jamaica	2607.6 (1933.7, 3416.3)	-34.0 (-47.0, -18.0)	92.8 (68.8, 121.5)	-44.5 (-56.0, -31.0)	87.4 (64.9, 114.0)	-47.0 (-57.0, -35.0)	-2.19 (-2.48, -1.91)
Indonesia	65016.0 (55139.9, 76512.6)	-47.8 (-58.0, -32.0)	25.1 (21.3, 29.5)	-62.7 (-70.0, -51.0)	26.1 (22.2, 30.5)	-67.0 (-73.0, -58.0)	-4.14 (-4.34, -3.95)
Egypt	75842.0 (52742.1, 103265.3)	-49.9 (-66.0, -31.0)	76.6 (53.2, 104.2)	-71.9 (-81.0, -61.0)	84.7 (59.0, 115.0)	-68.0 (-77.0, -57.0)	-2.82 (-3.12, -2.52)
Paraguay	4717.8 (3436.5, 6404.3)	45.4 (21.0, 68.0)	68.1 (49.6, 92.4)	-15.1 (-30.0, -2.00)	68.2 (49.7, 90.5)	-27.0 (-40.0, -15.0)	-1.05 (-1.22, -0.89)
Saint Vincent and the Grenadines	127.7 (99.9, 159.4)	-25.5 (-39.0, -11.0)	112.9 (88.3, 140.7)	-27.5 (-40.0, -13.0)	105.3 (82.1, 131.7)	-43.0 (-52.0, -28.0)	-2.09 (-2.93, -1.23)
Algeria	21690.7 (16989.6, 26888.1)	-25.3 (-44.0, 2.00)	51.8 (40.6, 64.3)	-54.9 (-66.0, -38.0)	54.4 (43.0, 67.1)	-61.9 (-72.0, -48.0)	-3.28 (-3.37, -3.19)
Suriname	468.1 (362.1, 590.0)	2.51 (-15.0, 27.0)	81.3 (62.9, 102.5)	-31.2 (-43.0, -14.0)	78.5 (60.7, 99.4)	-37.1 (-48.0, -23.0)	-1.96 (-2.44, -1.47)
Panama	2621.6 (1866.7, 3578.6)	-8.19 (-27.0, 11.0)	63.0 (44.9, 86.0)	-47.3 (-58.0, -36.0)	62.3 (44.4, 85.1)	-51.7 (-62.0, -41.0)	-2.92 (-3.12, -2.73)
Turkmenistan	6623.5 (5112.3, 8467.4)	-19.2 (-38.0, 3.00)	130.3 (100.6, 166.6)	-41.1 (-54.0, -25.0)	129.7 (100.9, 164.7)	-51.6 (-62.0, -38.0)	-3.46 (-3.67, -3.25)
Peru	19441.1 (13542.0, 27112.2)	8.88 (-12.0, 29.0)	57.2 (39.8, 79.8)	-34.0 (-44.0, -18.0)	56.3 (39.4, 78.3)	-42.4 (-55.0, -31.0)	-2.14 (-2.25, -2.03)
Mauritius	757.0 (578.8, 968.7)	-53.5 (-64.0, -42.0)	59.3 (45.3, 75.9)	-60.0 (-69.0, -50.0)	53.7 (40.5, 69.8)	-65.4 (-73.0, -57.0)	-3.09 (-3.40, -2.77)
Albania	1989.1 (1470.5, 2591.7)	-57.3 (-66.0, -48.0)	73.1 (54.1, 95.3)	-48.0 (-59.0, -37.0)	64.8 (46.7, 85.3)	-62.3 (-71.0, -54.0)	-3.21 (-3.38, -3.03)

Uzbekistan	77185.7 (61417.1, 96254.9)	17.0 (-6.00, 44.0)	229.2 (182.4, 285.8)	-27.2 (-42.0, -10.0)	240.3 (192.9, 297.3)	-39.4 (-51.0, -26.0)	-2.32 (-2.52, -2.12)
Brazil	184224.8 (143687.3, 238145.9)	-6.82 (-17.0, 3.00)	85.0 (66.3, 109.9)	-36.0 (-43.0, -29.0)	79.3 (61.6, 102.6)	-45.1 (-52.0, -38.0)	-2.09 (-2.19, -2.00)
Tonga	135.3 (105.4, 170.7)	-20.0 (-37.0, 5.00)	132.2 (103.0, 166.8)	-24.4 (-41.0, -1.00)	142.0 (111.4, 178.9)	-33.0 (-48.0, -11.0)	-1.43 (-2.23, -0.63)
Ecuador	11935.0 (8774.4, 15914.8)	7.34 (-11.0, 26.0)	67.9 (49.9, 90.5)	-38.8 (-49.0, -28.0)	69.6 (51.8, 91.2)	-49.9 (-59.0, -40.0)	-2.38 (-2.47, -2.29)
Samoa	576.0 (400.8, 792.5)	-31.0 (-37.0, 51.0)	272.5 (189.7, 375.0)	-22.8 (-51.0, 17.0)	300.8 (208.1, 412.4)	-31.9 (-57.0, 4.00)	-1.21 (-1.63, -0.80)
Cuba	10422.3 (7905.9, 13400.9)	-34.0 (-46.0, -21.0)	91.8 (69.6, 118.0)	-37.0 (-48.0, -24.0)	79.4 (59.5, 104.3)	-43.6 (-54.0, -33.0)	-1.94 (-2.05, -1.84)
Equatorial Guinea	1102.4 (730.5, 1618.7)	18.9 (-24.0, 79.0)	77.6 (51.5, 114.0)	-64.0 (-77.0, -46.0)	95.2 (66.2, 133.4)	-68.5 (-80.0, -49.0)	-4.4 (-4.74, -4.06)
Colombia	6220.0 (4708.6, 8125.6)	-64.0 (-73.0, -52.0)	13.0 (9.86, 17.0)	-75.5 (-82.0, -67.0)	12.1 (9.18, 15.7)	-82.5 (-87.0, -77.0)	-6.39 (-6.52, -6.26)
Tunisia	3517.3 (2402.1, 4883.8)	-46.0 (-65.0, -9.00)	30.4 (20.8, 42.2)	-56.7 (-74.0, -34.0)	28.3 (19.6, 39.1)	-66.1 (-80.0, -48.0)	-3.83 (-3.97, -3.68)
Republic of Moldova	2687.0 (2178.7, 3340.5)	-78.2 (-83.0, -73.0)	72.9 (59.1, 90.6)	-73.7 (-79.0, -67.0)	52.3 (42.4, 64.8)	-85 (-84.0, -76.0)	-6.25 (-6.51, -5.98)
Low-middle SDI							
Maldives	270.3 (202.2, 355.9)	-36.8 (-56.0, -11.0)	54.2 (40.6, 71.4)	-71.8 (-81.0, -60.0)	56.4 (43.5, 72.1)	-75.5 (-83.0, -66.0)	-5.16 (-5.74, -4.57)
Philippines	143263.7 (115350.5, 169313.5)	178.7 (77.0, 257.0)	127.8 (102.9, 151.0)	57.3 (0, 102.0)	126.9 (102.3, 150.0)	41.5 (-4.00, 81.0)	2.06 (1.82, 2.30)

Ghana	22478.1 (16396.7, 30329.8)	48.0 (16.0, 86.0)	71.3 (52.0, 96.2)	-29.5 (-45.0, -11.0)	84.5 (64.3, 109.0)	-39.6 (-53.0, -23.0)	-1.49 (-1.59, -1.38)
Myanmar	55658.6 (41475.8, 72656.0)	-37.4 (-58.0, -9.00)	101.8 (75.9, 132.9)	-53.0 (-69.0, -32.0)	103.5 (78.0, 134.2)	-57.9 (-72.0, -39.0)	-3.20 (-3.27, -3.14)
Sudan	36327.1 (24731.6, 52486.1)	-34.8 (-59.0, 1.00)	89.0 (60.6, 128.6)	-67.7 (-80.0, -50.0)	108.5 (75.0, 156.3)	-63.2 (-75.0, -47.0)	-2.90 (-3.16, -2.65)
Zambia	18595.1 (13828.8, 24791.6)	42.3 (9.00, 85.0)	102.0 (75.8, 135.9)	-38.0 (-52.0, -19.0)	126.3 (97.4, 162.7)	-33.9 (-50.0, -17.0)	-1.55 (-1.80, -1.30)
Democratic People's Republic of Korea	64793.6 (41729.6, 93720.2)	14.1 (-27.0, 82.0)	247.0 (159.1, 357.3)	-8.39 (-41.0, 46.0)	206.9 (133.7, 297.6)	-35.5 (-58.0, 0)	-1.50 (-1.62, -1.39)
Dominican Republic	9706.1 (7097.4, 12999.6)	2.51 (-24.0, 28.0)	89.2 (65.2, 119.5)	-32.2 (-49.0, -15.0)	89.2 (65.4, 118.9)	-35.3 (-52.0, -19.0)	-1.26 (-1.44, -1.08)
Guatemala	9248.3 (6212.2, 13426.5)	76.1 (44.0, 103.0)	52.0 (35.0, 75.5)	-21.1 (-35.0, -9.00)	53.6 (37.1, 76.0)	-33.8 (-46.0, -23.0)	-1.39 (-1.52, -1.25)
El Salvador	3565.6 (2451.2, 4984.1)	-8.58 (-24.0, 5.00)	57.0 (39.2, 79.7)	-23.0 (-36.0, -12.0)	56.5 (39.0, 78.8)	-34.9 (-46.0, -24.0)	-1.86 (-2.03, -1.70)
Honduras	5223.0 (3486.5, 7405.2)	115.6 (88.0, 142.0)	53.2 (35.5, 75.5)	3.41 (-10.0, 16.0)	58.6 (40.7, 80.5)	-14.8 (-30.0, -3.00)	-0.56 (-0.73, -0.38)
Timor-Leste	1583.2 (1007.8, 2153.5)	23.5 (-29.0, 85.0)	118.6 (75.5, 161.3)	-27.5 (-59.0, 9.00)	133.8 (87.5, 185.6)	-33.7 (-60.0, -3.00)	-1.55 (-1.84, -1.26)
Guyana	962.7 (732.1, 1233.2)	-34.5 (-50.0, -16.0)	124.9 (95.0, 160.0)	-34.6 (-50.0, -16.0)	122.9 (93.5, 157.2)	-43.3 (-57.0, -27.0)	-1.88 (-2.21, -1.56)
Namibia	2366.1 (1715.1, 3181.8)	-3.04 (-29.0, 36.0)	98.5 (71.4, 132.4)	-43.1 (-58.0, -20.0)	110.8 (82.0, 145.3)	-58.0 (-64.0, -31.0)	-2.65 (-2.84, -2.45)

Kyrgyzstan	9230.9 (7400.8, 11242.1)	-41.0 (-52.0, -28.0)	141.2 (113.2, 172.0)	-59.1 (-67.0, -51.0)	148.6 (120.0, 181.1)	-63.9 (-71.0, -56.0)	-3.95 (-4.09, -3.81)
Tajikistan	13084.7 (10584.6, 16314.0)	-13.6 (-30.0, 6.00)	137.8 (111.5, 171.9)	-51.0 (-60.0, -40.0)	157.1 (128.4, 194.0)	-58.8 (-66.0, -49.0)	-3.42 (-3.59, -3.24)
India	4569987.7 (3561093.8, 5466118.0)	7.82 (-14.0, 32.0)	328.6 (256.1, 393.1)	-33.7 (-47.0, -19.0)	350.7 (271.9, 420.8)	-47.1 (-58.0, -34.0)	-2.43 (-2.56, -2.30)
Tuvalu	45.0 (30.4, 66.0)	-37.2 (-57.0, -7.00)	381.8 (257.9, 559.5)	-52.0 (-66.0, -26.0)	393.7 (266.0, 573.4)	-53.5 (-68.0, -32.0)	-2.41 (-3.69, -1.11)
Nicaragua	3865.6 (2648.1, 5553.2)	13.1 (-8.00, 32.0)	59.4 (40.7, 85.3)	-32.5 (-45.0, -21.0)	60.4 (42.3, 85.2)	-43.4 (-55.0, -33.0)	-2.53 (-2.71, -2.35)
Viet Nam	46205.4 (33404.6, 59187.9)	-26.8 (-54.0, 11.0)	47.9 (34.7, 61.4)	-48.4 (-68.0, -22.0)	47.3 (34.6, 59.5)	-64.2 (-78.0, -46.0)	-3.83 (-3.88, -3.78)
Kenya	42277.1 (29827.4, 59682.6)	106.0 (65.0, 144.0)	84.2 (59.4, 118.8)	-7.38 (-24.0, 13.0)	96.1 (66.2, 132.5)	-19.3 (-33.0, -3.00)	-0.66 (-0.86, -0.46)
Kiribati	784.8 (575.5, 1039.7)	6.15 (-28.0, 51.0)	661.6 (485.2, 876.5)	-33.7 (-55.0, -6.00)	733.8 (543.6, 967.3)	-38.6 (-58.0, -14.0)	-1.73 (-2.11, -1.34)
Nigeria	165383.3 (118837.0, 232823.0)	6.67 (-26.0, 43.0)	77.0 (55.3, 108.4)	-55.2 (-69.0, -40.0)	96.3 (70.9, 133.4)	-56.7 (-69.0, -41.0)	-2.86 (-3.02, -2.69)
Lesotho	3449.6 (2542.6, 4568.8)	-31.0 (-30.0, 37.0)	164.9 (121.6, 218.4)	-13.9 (-39.0, 18.0)	184.6 (136.6, 246.5)	-23.5 (-48.0, 8.00)	-0.22 (-0.39, -0.05)
Palestine	1718.1 (1205.6, 2397.1)	47.8 (8.00, 94.0)	34.7 (24.3, 48.4)	-38.3 (-55.0, -19.0)	41.6 (31.0, 54.9)	-49.4 (-64.0, -33.0)	-2.55 (-2.82, -2.29)
Morocco	29461.9 (22495.3, 37588.0)	-24.4 (-43.0, -1.00)	82.0 (62.6, 104.6)	-46.8 (-60.0, -30.0)	83.8 (64.7, 106.1)	-54.8 (-66.0, -40.0)	-2.73 (-2.84, -2.62)

Mauritania	2615.8 (1854.3, 3611.1)	-7.99 (-31.0, 19.0)	65.2 (46.2, 90.0)	-52.6 (-64.0, -39.0)	79.9 (59.5, 105.5)	-58.6 (-69.0, -46.0)	-2.58 (-2.77, -2.38)
Marshall Islands	307.0 (208.8, 440.8)	22.1 (-15.0, 69.0)	540.1 (367.3, 775.5)	-1.74 (-32.0, 36.0)	572.7 (382.8, 822.1)	-28.0 (-50.0, -3.00)	-1.16 (-1.79, -0.52)
Venezuela (Bolivarian Republic of)	6112.3 (4439.7, 8311.4)	-41.3 (-58.0, -19.0)	21.8 (15.8, 29.6)	-66.0 (-72.0, -46.0)	20.8 (15.2, 28.1)	-69.5 (-78.0, -58.0)	-5.10 (-5.27, -4.93)
Mongolia	5364.9 (4181.6, 6807.2)	1.23 (-28.0, 43.0)	158.4 (123.4, 200.9)	-35.6 (-54.0, -9.00)	175.5 (138.4, 221.8)	-55.6 (-69.0, -36.0)	-3.36 (-3.51, -3.22)
Eswatini	1415.2 (1027.8, 1893.3)	3.66 (-25.0, 41.0)	123.9 (90.0, 165.8)	-26.8 (-47.0, -1.00)	140.4 (103.5, 187.1)	-40.0 (-57.0, -15.0)	-1.44 (-1.71, -1.16)
Micronesia (Federated States of)	493.9 (284.3, 741.5)	-26.2 (-57.0, 13.0)	483.7 (278.4, 726.1)	-24.7 (-56.0, 16.0)	513.7 (302.1, 765.9)	-49.0 (-65.0, -10.0)	-1.89 (-2.31, -1.46)
Lao People's Democratic Republic	11669.8 (8393.0, 15422.8)	1.50 (-32.0, 49.0)	163.0 (117.3, 215.5)	-41.1 (-60.0, -14.0)	168.5 (122.1, 220.0)	-49.0 (-65.0, -27.0)	-2.43 (-2.58, -2.27)
Sao Tome and Principe	239.2 (172.7, 316.3)	8.13 (-20.0, 45.0)	116.5 (84.1, 154.0)	-36.0 (-53.0, -14.0)	153.0 (108.4, 204.7)	-36.9 (-54.0, -15.0)	-1.90 (-2.55, -1.24)
Bolivia (Plurinational State of)	10862.2 (8054.0, 14119.6)	-3.75 (-26.0, 23.0)	90.4 (67.1, 117.6)	-48.6 (-61.0, -34.0)	102.9 (77.1, 132.0)	-57.3 (-68.0, -45.0)	-3.07 (-3.18, -2.96)
Cameroon	22041.0 (15474.8, 30449.0)	65.4 (21.0, 119.0)	75.7 (53.2, 104.6)	-49.0 (-57.0, -22.0)	98.6 (71.7, 133.2)	-48.3 (-62.0, -31.0)	-2.03 (-2.14, -1.93)
Congo	5570.1 (4037.9, 7489.4)	26.5 (-4.00, 64.0)	105.8 (76.7, 142.2)	-41.3 (-56.0, -24.0)	129.1 (95.6, 170.2)	-49.7 (-63.0, -33.0)	-2.68 (-2.82, -2.54)
Syrian Arab Republic	11044.2 (8320.8, 14304.0)	-73.7 (-82.0, -62.0)	76.2 (57.4, 98.7)	-76.6 (-84.0, -66.0)	78.2 (59.6, 100.3)	-83.0 (-87.0, -72.0)	-7.45 (-7.69, -7.21)

Belize	330.2 (247.0, 429.1)	89.7 (59.0, 124.0)	80.5 (60.2, 104.6)	-14.0 (-28.0, 1.00)	80.3 (60.9, 102.3)	-27.3 (-40.0, -13.0)	-1.71 (-2.45, -0.96)
Cabo Verde	401.7 (286.7, 548.5)	-38.1 (-52.0, -21.0)	71.3 (50.9, 97.3)	-61.4 (-70.0, -51.0)	74.5 (54.9, 99.8)	-68.4 (-75.0, -60.0)	-3.70 (-4.17, -3.23)
Nauru	40.7 (25.5, 55.8)	-14.1 (-42.0, 23.0)	385.5 (242.0, 528.7)	-16.6 (-44.0, 20.0)	427.2 (277.1, 577.4)	-24.9 (-49.0, 9.00)	-0.82 (-2.63, 1.03)
Low SDI							
Vanuatu	1619.06 (1065.17, 2414.9)	98.8 (34.0, 205.0)	549.7 (361.6, 819.9)	2.16 (-31.0, 57.0)	627.2 (410.4, 941.1)	-12.7 (-42.0, 31.0)	-0.76 (-1.06, -0.46)
Yemen	34778.2 (23967.3, 49266.1)	27.0 (-22.0, 84.0)	110.4 (76.1, 156.4)	-47.4 (-66.0, -20.0)	148.1 (101.9, 212.7)	-46.8 (-65.0, -21.0)	-2.27 (-2.46, -2.07)
Uganda	41039.1 (28633.0, 58648.8)	84.2 (35.0, 144.0)	99.8 (69.6, 142.6)	-22.4 (-43.0, 3.00)	116.8 (86.2, 159.7)	-28.2 (-46.0, -7.00)	-1.66 (-1.90, -1.43)
Gambia	1802.9 (1315.9, 2423.2)	52.8 (11.0, 110.0)	80.3 (58.6, 107.9)	-32.5 (-51.0, -7.00)	106.1 (80.0, 136.1)	-37.9 (-55.0, -15.0)	-1.43 (-1.71, -1.15)
Afghanistan	50841.7 (36190.2, 71430.1)	44.7 (8.00, 103.0)	132.8 (94.6, 186.6)	-56.8 (-68.0, -40.0)	210.7 (141.0, 302.2)	-47.3 (-61.0, -27.0)	-2.50 (-2.61, -2.39)
Djibouti	1128.0 (769.5, 1600.9)	82.8 (35.0, 139.0)	93.8 (64.0, 133.1)	-26.1 (-46.0, -4.00)	104.9 (72.6, 145.1)	-34.4 (-51.0, -14.0)	-1.61 (-2.02, -1.20)
Guinea	12379.7 (9313.5, 15876.6)	1.08 (-26.0, 36.0)	97.9 (73.7, 125.6)	-55.0 (-64.0, -34.0)	131.4 (100.2, 169.6)	-46.9 (-61.0, -31.0)	-1.58 (-1.73, -1.42)
Burundi	14290.5 (10366.5, 19197.4)	16.1 (-15.0, 55.0)	119.7 (86.9, 160.9)	-45.8 (-60.0, -28.0)	144.7 (106.7, 191.1)	-44.4 (-58.0, -27.0)	-2.32 (-2.50, -2.13)
Bangladesh	163997.2 (128860.7, 202582.8)	-79.0 (-26.0, 32.0)	103.0 (80.9, 127.2)	-32.1 (-49.0, -10.0)	105.4 (82.1, 130.4)	-43.2 (-57.0, -27.0)	-2.10 (-2.30, -1.90)

Bhutan	2249.2 (1245.5, 4139.3)	-22.7 (-52.0, 32.0)	298.2 (165.1, 548.8)	-37.3 (-61.0, 7.00)	338.5 (182.4, 639.1)	-54.4 (-73.0, -27.0)	-3.16 (-3.35, -2.98)
Eritrea	8836.0 (6567.8, 12114.3)	41.6 (4.00, 88.0)	131.7 (97.9, 180.5)	-36.7 (-53.0, -16.0)	160.1 (123.4, 209.0)	-39.8 (-56.0, -20.0)	-1.64 (-1.83, -1.45)
Cambodia	19083.3 (13396.1, 26076.7)	-36.0 (-47.0, -10.0)	114.9 (80.7, 157.1)	-56.7 (-67.0, -44.0)	119.7 (84.6, 163.5)	-63.3 (-72.0, -52.0)	-3.65 (-3.76, -3.55)
Comoros	753.2 (544.7, 1018.1)	7.59 (-27.0, 103.0)	105.4 (76.3, 142.5)	-29.9 (-53.0, 32.0)	112.8 (83.1, 148.5)	-37.9 (-56.0, 8.00)	-2.02 (-2.41, -1.63)
Guinea-Bissau	2174.3 (1589.4, 2828.1)	-14.4 (-43.0, 31.0)	114.4 (83.6, 148.8)	-54.6 (-70.0, -31.0)	160.4 (119.0, 203.7)	-55.1 (-69.0, -34.0)	-2.33 (-2.54, -2.11)
Liberia	3856.2 (2729.9, 5290.2)	13.8 (-20.0, 54.0)	80.5 (57.0, 110.4)	-53.3 (-67.0, -37.0)	102.3 (72.8, 141.0)	-51.0 (-64.0, -34.0)	-2.33 (-2.69, -1.97)
Madagascar	41993.7 (30983.4, 58419.7)	26.0 (-7.00, 72.0)	157.3 (116.1, 218.9)	-43.6 (-58.0, -23.0)	186.6 (140.3, 243.0)	-39.1 (-54.0, -21.0)	-1.52 (-1.75, -1.29)
Mali	23291.0 (16638.8, 31354.6)	16.8 (-13.0, 54.0)	106.3 (75.9, 143.1)	-53.8 (-66.0, -39.0)	140.3 (103.6, 185.1)	-53.1 (-64.0, -40.0)	-2.48 (-2.62, -2.34)
Haiti	38645.3 (24267.9, 59620.1)	-5.44 (-32.0, 29.0)	311.6 (195.7, 480.7)	-51.5 (-65.0, -34.0)	341.8 (212.7, 524.1)	-53.7 (-67.0, -38.0)	-2.39 (-2.55, -2.24)
Pakistan	927937.4 (687813.9, 1173407.3)	57.4 (18.0, 104.0)	414.1 (307.0, 523.7)	-27.0 (-41.0, 3.00)	542.0 (407.1, 691.8)	-27.9 (-48.0, -6.00)	-1.40 (-1.59, -1.22)
Ethiopia	99992.1 (72904.9, 133534.7)	-9.61 (-34.0, 35.0)	92.9 (67.8, 124.1)	-56.8 (-69.0, -36.0)	112.1 (84.0, 145.9)	-57.4 (-68.0, -41.0)	-3.26 (-3.46, -3.06)
Zimbabwe	36406.9 (26568.6, 47135.5)	62.0 (14.0, 122.0)	242.5 (177.0, 314.0)	13.0 (-22.0, 53.0)	261.4 (187.9, 340.6)	2.87 (-27.0, 39.0)	1.12 (0.83, 1.42)

Mozambique	28990.3 (20450.3, 39198.6)	62.3 (26.0, 103.0)	98.2 (69.3, 132.8)	-28.2 (-44.0, -10.0)	126.0 (94.6, 163.2)	-24.8 (-42.0, -7.00)	-0.82 (-1.08, -0.55)
Angola	31416.7 (23036.1, 41772.9)	52.3 (11.0, 103.0)	104.2 (76.4, 138.6)	-47.9 (-62.0, -31.0)	143.0 (108.4, 186.3)	-47.3 (-61.0, -29.0)	-2.33 (-2.50, -2.16)
South Sudan	9290.9 (6225.3, 13491.8)	6.84 (-21.0, 46.0)	100.1 (67.1, 145.3)	-32.6 (-50.0, -8.00)	118.3 (80.0, 167.8)	-31.9 (-48.0, -9.00)	-1.26 (-1.50, -1.01)
Nepal	91251.6 (62074.7, 126784.4)	-8.10 (-35.0, 29.0)	300.0 (204.1, 416.8)	-41.0 (-58.0, -17.0)	358.6 (240.4, 506.6)	-51.9 (-67.0, -31.0)	-2.84 (-2.95, -2.74)
Burkina Faso	20916.4 (15404.2, 28701.8)	81.4 (38.0, 139.0)	92.2 (67.9, 126.5)	-23.6 (-42.0, 1.00)	115.7 (89.5, 145.4)	-22.1 (-39.0, -4.00)	-0.76 (-0.90, -0.62)
Niger	24207.1 (16693.0, 35859.1)	33.3 (-9.00, 93.0)	103.9 (71.7, 153.9)	-54.1 (-69.0, -34.0)	146.2 (102.8, 217.1)	-49.6 (-62.0, -32.0)	-2.05 (-2.26, -1.83)
Democratic Republic of the Congo	105450.3 (72529.7, 153309.2)	51.9 (13.0, 105.0)	120.3 (82.7, 174.9)	-33.1 (-50.0, -10.0)	165.4 (113.0, 252.8)	-33.7 (-51.0, -7.00)	-1.40 (-1.54, -1.25)
Malawi	18795.5 (13528.8, 25908.7)	18.5 (-14.0, 71.0)	101.92 (73.36, 140.49)	-38.6 (-55.0, -11.0)	116.1 (87.7, 151.8)	-37.8 (-52.0, -17.0)	-1.74 (-2.03, -1.44)
Papua New Guinea	59208.0 (36098.9, 90334.1)	114.4 (63.0, 193.0)	600.1 (365.9, 915.6)	-11.2 (-33.0, 21.0)	689.0 (415.1, 1081.7)	-17.4 (-37.0, 13.0)	-0.43 (-0.49, -0.36)
Central African Republic	10250.0 (6931.6, 14810.8)	44.6 (8.00, 94.0)	193.4 (130.8, 279.5)	-25.2 (-44.0, 0)	270.1 (174.0, 406.6)	-26.0 (-46.0, 0)	-1.11 (-1.22, -1.01)
Benin	11055.3 (8023.2, 14695.3)	39.2 (-1.00, 84.0)	87.3 (63.4, 116.0)	-46.7 (-62.0, -29.0)	115.7 (88.4, 148.9)	-48.6 (-61.0, -35.0)	-1.97 (-2.10, -1.84)
Rwanda	12656.3 (9019.7, 17484.9)	-23.4 (-43.0, 4.00)	99.8 (71.1, 137.8)	-56.7 (-68.0, -41.0)	114.2 (81.9, 151.3)	-59.8 (-70.0, -46.0)	-3.93 (-4.10, -3.76)

Senegal	12089.6 (8705.1, 16263.7)	15.1 (-16.0, 54.0)	79.9 (57.5, 107.5)	-42.0 (-58.0, -22.0)	100.4 (73.9, 131.0)	-45.1 (-57.0, -29.0)	-1.73 (-1.98, -1.48)
Solomon Islands	4362.1 (2174.5, 6592.8)	56.0 (6.00, 117.0)	665.3 (331.7, 1005.6)	-19.0 (-45.0, 13.0)	809.5 (404.5, 1227.9)	-31.3 (-53.0, -3.00)	-1.08 (-1.27, -0.90)
Togo	6782.5 (4897.1, 9137.3)	37.0 (2.00, 78.0)	85.6 (61.8, 115.4)	-36.7 (-53.0, -18.0)	109.6 (81.4, 144.9)	-44.9 (-58.0, -30.0)	-1.67 (-1.85, -1.48)
Sierra Leone	8147.7 (5719.7, 11070.5)	22.3 (-16.0, 72.0)	98.4 (69.0, 133.6)	-46.1 (-63.0, -24.0)	121.3 (86.9, 161.2)	-43.0 (-58.0, -23.0)	-1.35 (-1.58, -1.12)
United Republic of Tanzania	53121.3 (37596.8, 73631.2)	48.5 (14.0, 94.0)	93.6 (66.3, 129.8)	-32.2 (-48.0, -11.0)	107.8 (80.4, 143.5)	-33.6 (-47.0, -16.0)	-1.42 (-1.54, -1.31)
Somalia	31871.4 (20885.8, 47286.4)	103.3 (49.0, 183.0)	156.7 (102.7, 232.4)	-28.6 (-48.0, 0)	201.9 (129.9, 315.2)	-29.7 (-48.0, -5.00)	-1.36 (-1.58, -1.14)
Chad	16432.0 (12385.7, 21411.4)	51.5 (15.0, 98.0)	100.2 (75.5, 130.6)	-44.4 (-58.0, -27.0)	144.6 (111.7, 191.6)	-40.0 (-54.0, -24.0)	-1.40 (-1.56, -1.24)

Abbreviation: DALY, disability-adjusted life-years; GBD, Global Burden of Diseases, Injuries, and Risk Factors Study; RHD, rheumatic heart disease; SDI, Socio-demographic Index.

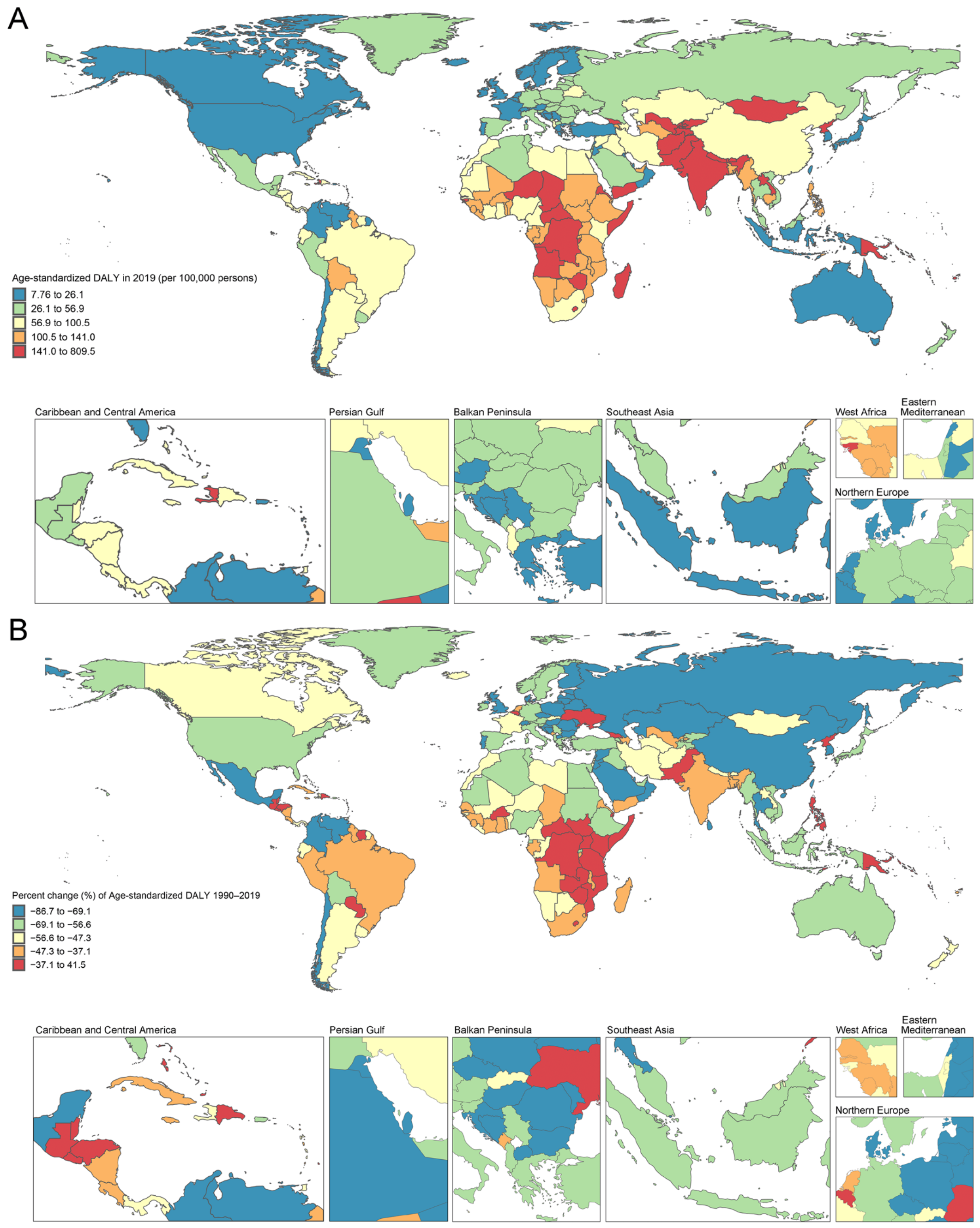

Supplementary Fig. 1. Age-standardized DALYs (per 100,000 persons) in 2019 and percent change (%) of age-standardized DALYs during 1990-2019 for RHD in 204 countries and territories.

A. World map of age-standardized DALYs for RHD in 2019; **B.** World map of percent change of age-standardized DALYs for RHD during 1990-2019.

Abbreviation: DALYs, disability-adjusted life-years; RHD, rheumatic heart disease.

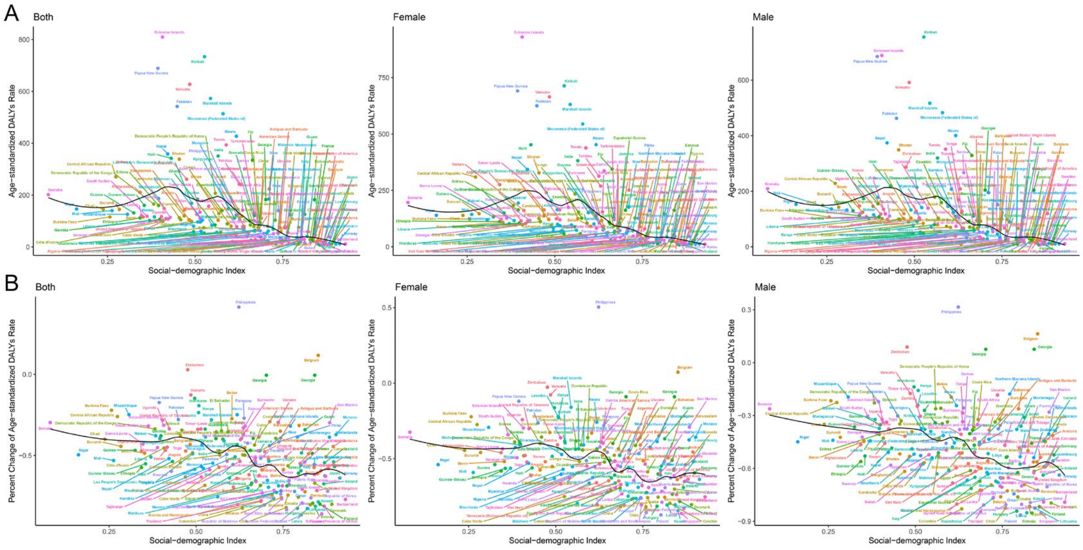

Supplementary Fig. 2. The relationship between SDI levels and the age-standardized DALYs (per 100,000 persons) in 2019 and percent change (%) of age-standardized DALYs during 1990-2019 for RHD in 204 countries and territories.

A. Age-standardized DALYs for RHD in 2019; **B.** Percent change of age-standardized DALYs for RHD during 1990-2019.

Abbreviation: DALYs, disability-adjusted life-years; RHD, rheumatic heart disease; SDI, socio-demographic index.

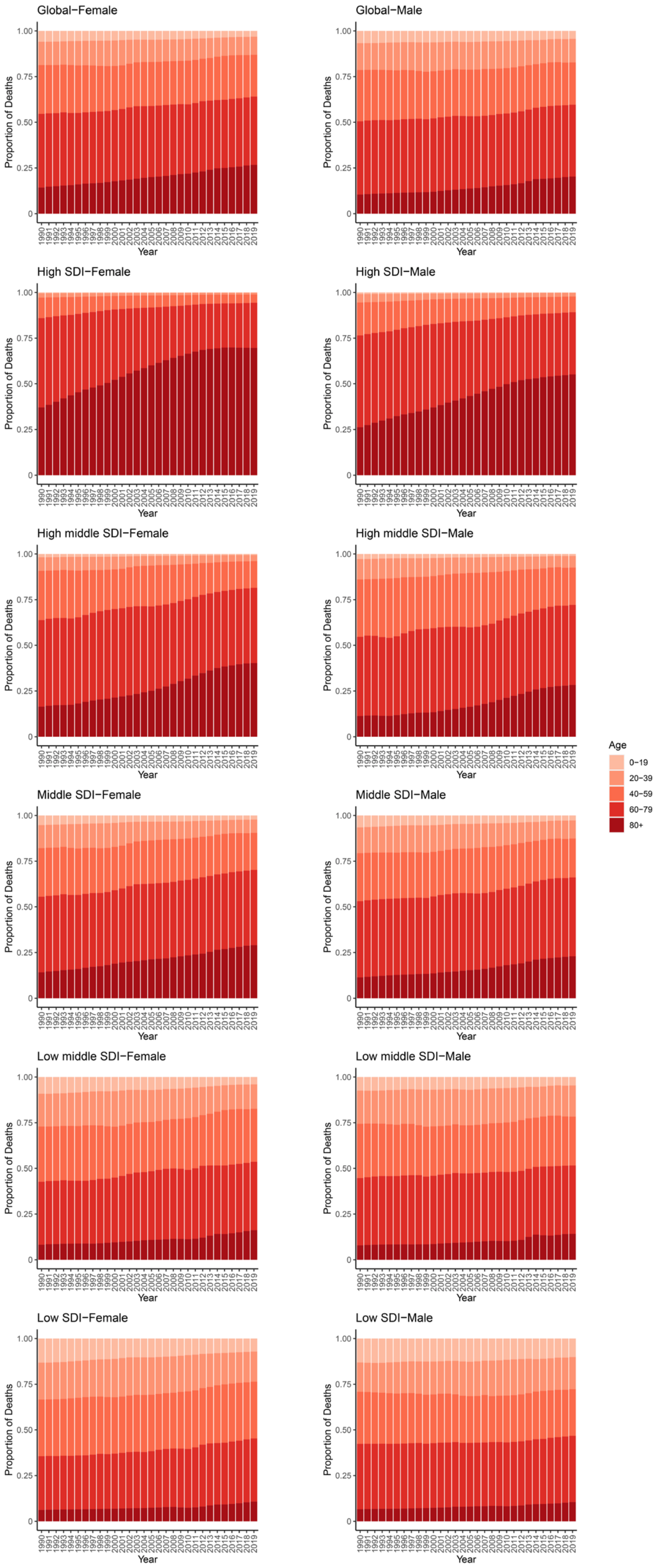

Supplementary Fig. 3. The temporal change in the sex-specific age distribution of deaths from RHD by SDI quintiles during 1990-2019.

Abbreviation: RHD, rheumatic heart disease; SDI, socio-demographic index.

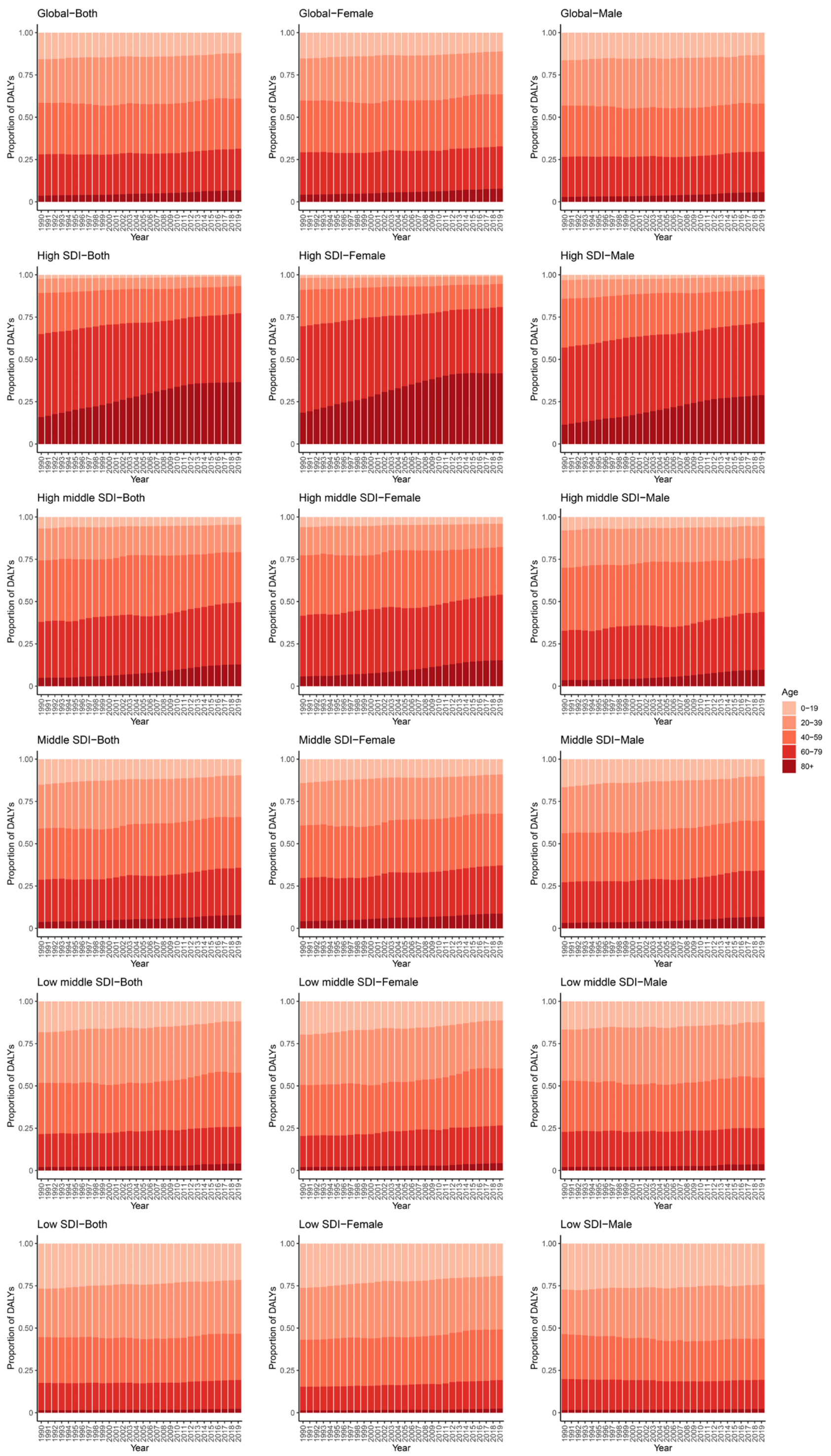

Supplementary Fig. 4. The temporal change in the sex-specific age distribution of DALYs from RHD by SDI quintiles during 1990-2019.

Abbreviation: DALYs, disability-adjusted life-years; RHD, rheumatic heart disease; SDI, socio-demographic index.

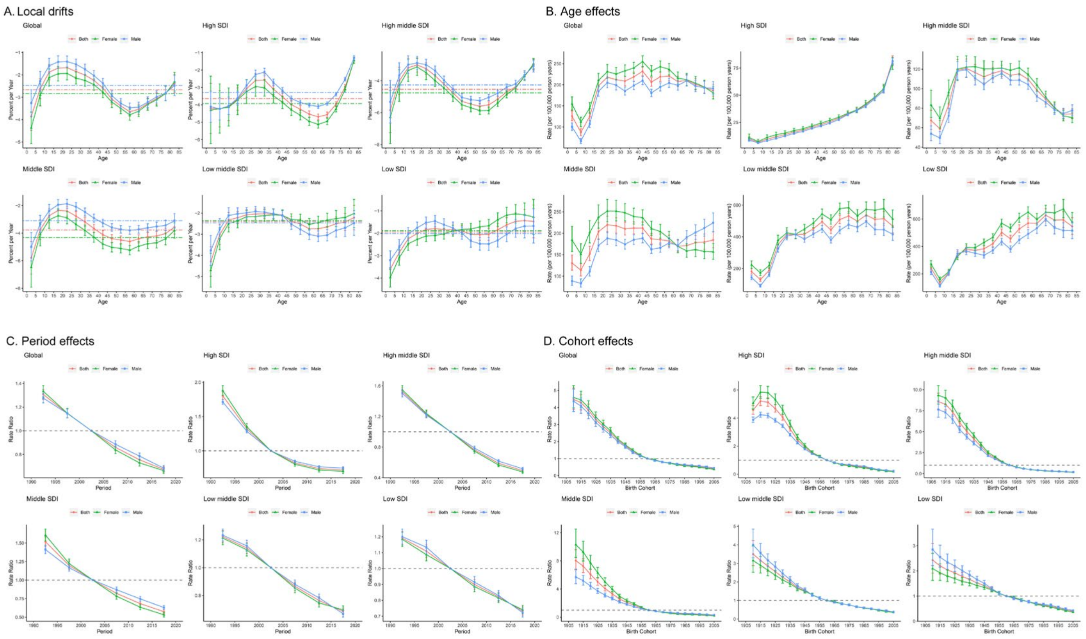

Supplementary Fig. 5. Local drifts, age, period, and cohort effects on DALYs from RHD by SDI quintiles. **A.** Local drifts; **B.** Age effects; **C.** Period effects; **D.** Cohort effects.

Abbreviation: DALYs, disability-adjusted life-years; RHD, rheumatic heart disease; SDI, socio-demographic index.

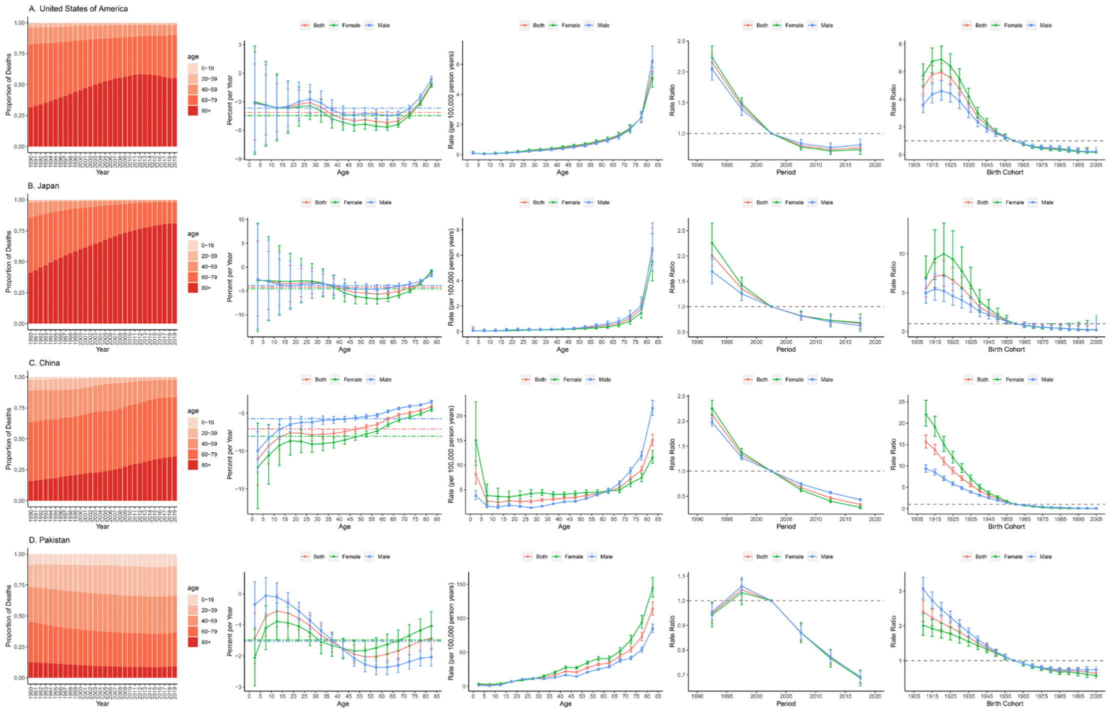

Supplementary Fig. 6. Age-period-cohort effects of deaths from RHD on exemplar countries. Each of the four horizontal graphs represents the temporal change in the relative proportion of RHD deaths from RHD, local drifts, age effects, period effects and cohort effects, respectively. **A.** The United States of America; **B.** Japan; **C.** China; **D.** Pakistan.

Abbreviation: RHD, rheumatic heart disease.

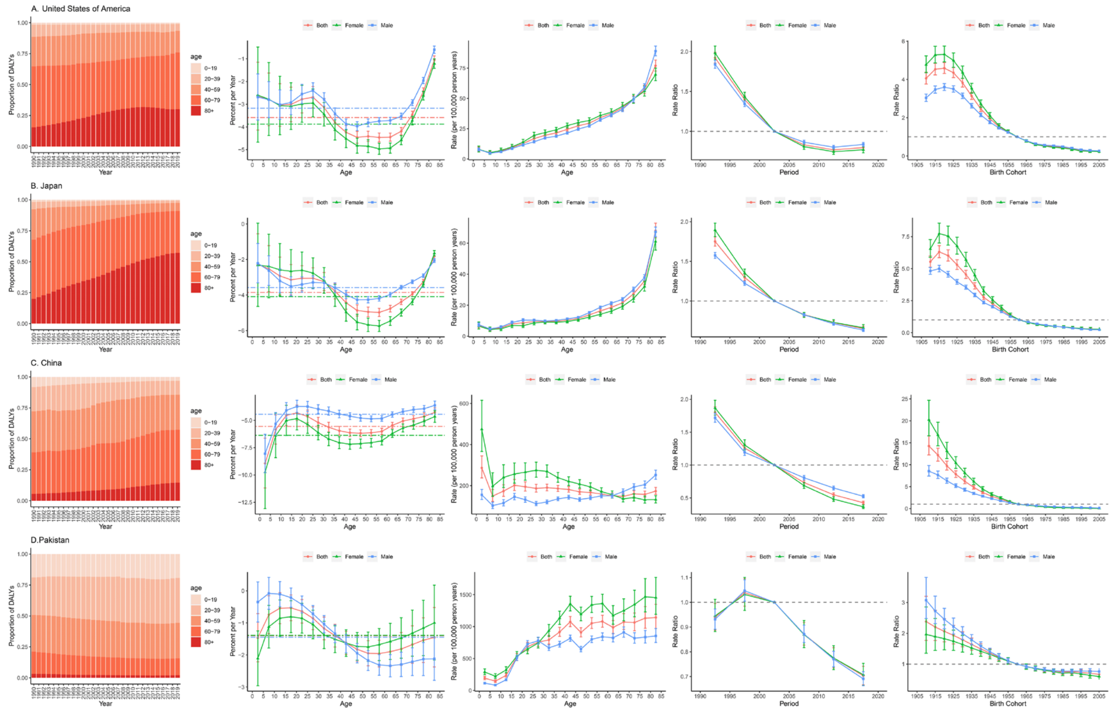

Supplementary Fig. 7. Age-period-cohort effects of DALYs from RHD on exemplar countries. Each of the four horizontal graphs represents the temporal change in the DALYs from RHD, local drifts, age effects, period effects and cohort effects, respectively.

Abbreviation: DALYs, disability-adjusted life-years; RHD, rheumatic heart disease.

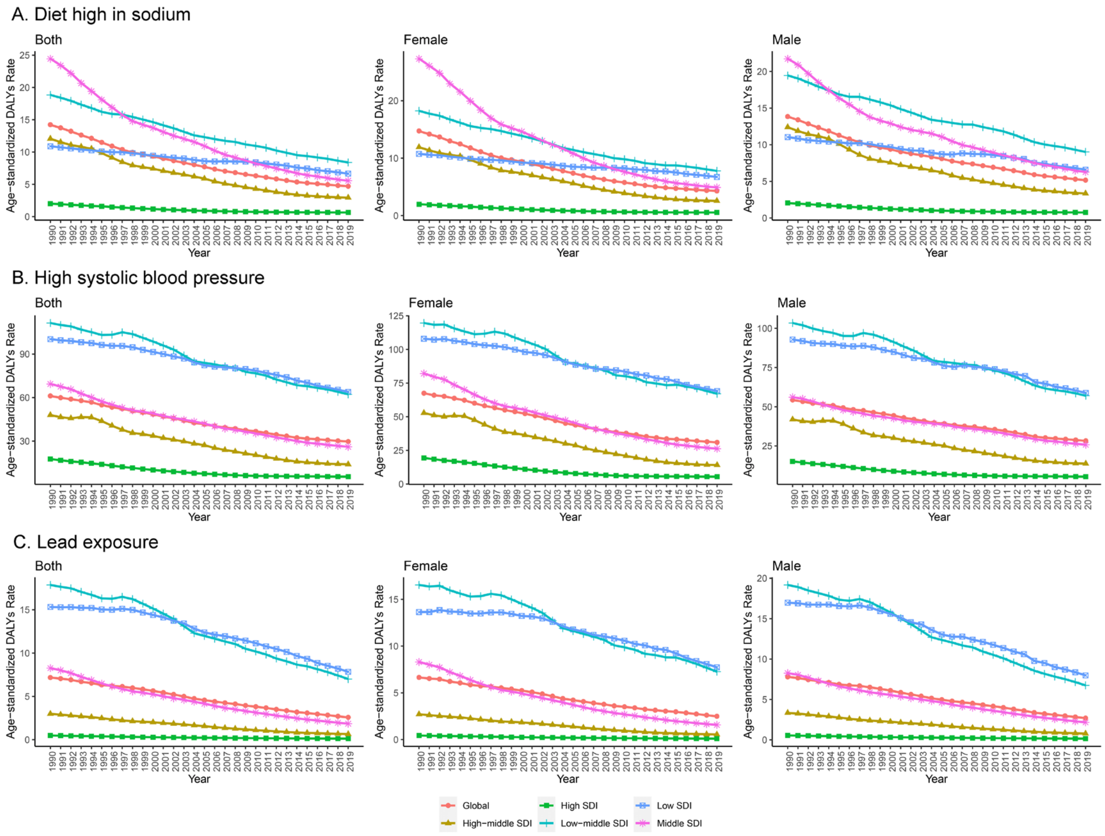

Supplementary Fig. 8. The leading risk factor and its period effects on global DALYs from RHD by SDI quintiles. **Abbreviation:** DALYs, disability-adjusted life-years; RHD, rheumatic heart disease; SDI, socio-demographic index.

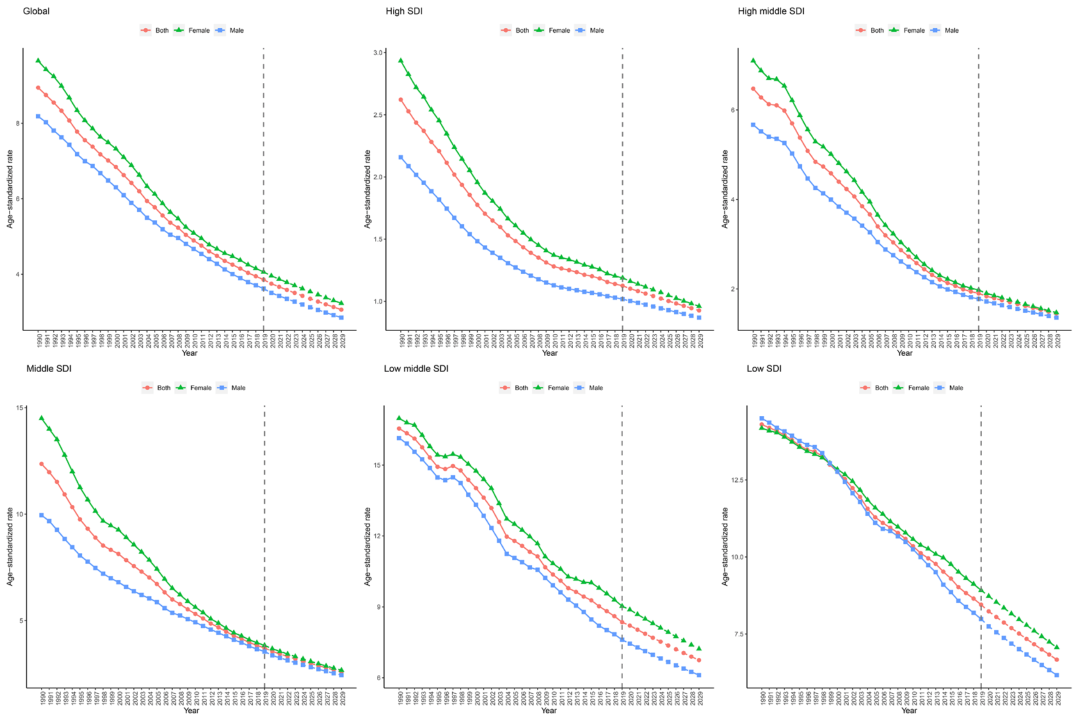

Supplementary Fig. 9. The age-standardized mortality rate of RHD by SDI quintiles over the upcoming decade predicted by the Bayesian age-period-cohort model.

Abbreviation: RHD, rheumatic heart disease; SDI, socio-demographic index.
